# Tuning the Structure–Functional Properties Within Peptide-Mimicking Antimicrobial Hydrogels

**DOI:** 10.3390/antibiotics14111118

**Published:** 2025-11-05

**Authors:** Samuel T. Attard, Vina R. Aldilla, Rajesh Kuppusamy, Renxun Chen, David StC Black, Pall Thordarson, Mark D. P. Willcox, Naresh Kumar

**Affiliations:** 1 School of Chemistry, The University of New South Wales (UNSW), Sydney, NSW 2052, Australia; vr.aldilla@gmail.com (V.R.A.); rajesh.kuppusamy@whiteley.com.au (R.K.); ren@brightenvironmental.com.au (R.C.); d.black@unsw.edu.au (D.S.B.); p.thordarson@unsw.edu.au (P.T.); 2 School of Optometry and Vision Science, The University of New South Wales (UNSW), Sydney, NSW 2052, Australia; m.willcox@unsw.edu.au

**Keywords:** antimicrobial, antimicrobial peptide-mimic, low-molecular weight hydrogel, self-assembling hydrogel

## Abstract

**Background/Objectives:** There is a growing demand for the development of novel antimicrobial agents due to their efficacy being eroded by increasing antimicrobial resistance. Antimicrobial hydrogels have been reported as a method to treat bacterial infections. **Methods:** This study explores how different structural features are important for the hydrogelation properties of amphiphilic antimicrobial peptide-mimics through rheology and AFM, as well as properties important for antimicrobial activity measured through MIC. **Results:** Eleven novel peptide-mimicking anthranilamides containing various structural features were synthesised in 4–7 steps. Of these peptide-mimics, three novel compounds formed hydrogels, and it was identified that their mechanical strength, secondary structure, and fibre morphology could be tuned by altering the aromatic cap or the amino acid side chain. In conjunction, several structural features were identified that reduce hydrogelation strength and stiffness. **Conclusions:** This work provides an insight into how the structural features of low-molecular-weight self-assembling hydrogels can translate to differing physical and potent antimicrobial properties. This work provides a rational guide to optimising physical hydrogel properties, as well as highlighting features that may reduce hydrogelation.

## 1. Introduction

Self-assembling hydrogels are defined as compounds that can supramolecularly organise themselves into three-dimensional fibrous networks that retain large volumes of water [1,2]. These materials can exhibit several potential applications, such as in drug delivery [3,4,5,6] and tissue engineering [7,8,9]. In addition, the high-moisture environment created by hydrogels has been reported to improve the rate of wound healing, highlighting the potential for its use as a wound dressing [10,11,12]. Concurrently, it also increases the risk of infection due to opportunistic bacteria, further delaying the wound healing process or causing further medical complications [13,14,15]. Therefore, hydrogels with antimicrobial properties could improve the wound healing process while reducing the rate and severity of infections in skin wounds.

Peptides can form hydrogels suitable for biomedical applications, with low toxicity, biodegradability, and non-immunogenic properties [16,17]. Peptides contain a diverse range of amino acids, including those with aromatic side chains that can facilitate π-π stacking interactions between the aromatic groups of low-molecular-weight gelators (LMWGs), promoting the formation of fibrous hydrogel networks [18,19,20,21]. However, due to the high costs of synthesis, the large-scale manufacturing of this class of material remains challenging.

Short peptide-based LMWGs have been explored due to their lower manufacturing cost and simpler synthetic route in comparison to longer peptides [22,23]. LMWGs are small molecules that are capable of generating self-assembled hydrogels following a trigger such as a temperature or pH change [24,25,26,27]. An aromatic capping group is commonly adjoined to the *N*-terminal of short peptide-based LMWGs to compensate for the number of π-π stacking interactions available in longer peptides, improving their hydrophobic properties and the stability of the fibres [28,29,30,31]. Some examples of reported aromatic caps are fluorenylmethoxycarbonyl (Fmoc) [32,33,34], naphthalene [35,36], and heterocyclic groups such as indoles [37], and benzimidazoles [38].

**Figure 1:**
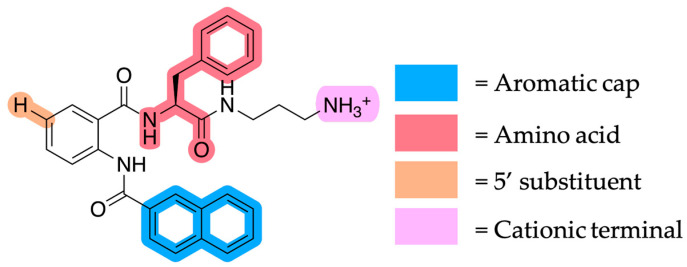
Structure of ultra-short hydrogelator **1**, highlighting the differing components of an AMP containing a naphthoyl aromatic cap [30].

Although antibacterial hydrogels have been reported, most require an antimicrobial component, such as metal ions like silver [39] or antimicrobials such as ciprofloxacin, to be either loaded or encapsulated within the gels [40,41,42]. Our previous work has highlighted the utilisation of short peptides accommodating either a glyoxylamide or anthranilamide scaffold to generate self-assembled hydrogels. These short peptides could be easily synthesised on a gram-scale using the ring-opening reactions of either isatin or isatoic anhydride in solution phase [30,31,43]. Additionally, the antibacterial activity of hydrogels made up of short cationic peptide **1** has been reported (Figure 1) [30,31,44,45]. Despite demonstrating antimicrobial activity and low toxicity, the relationship between chemical structure and hydrogelation capability, mechanical properties, physical features, and antibacterial properties remains largely unexplored.

**Figure 2:**
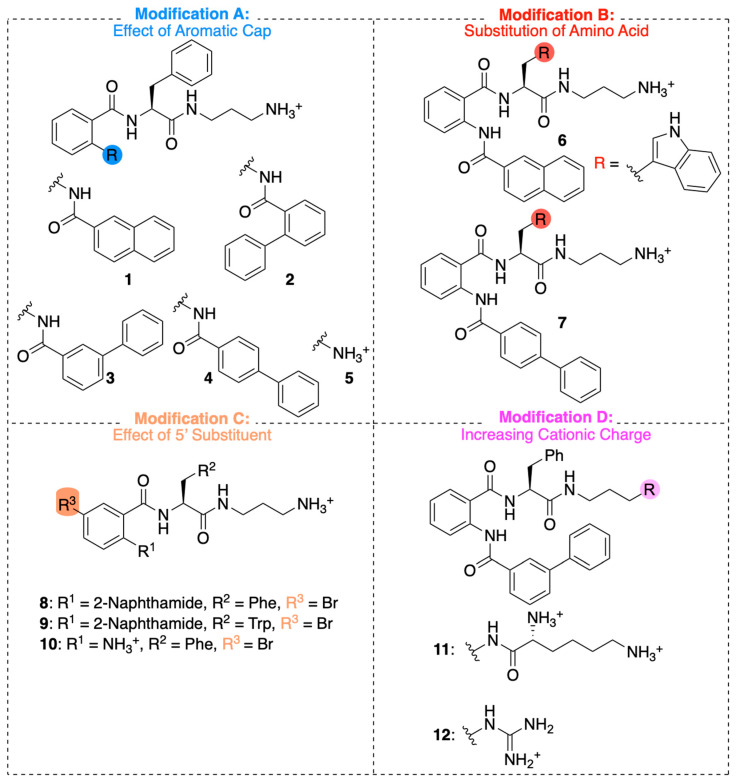
Structures of the modified peptide-mimic compounds.

This work aimed to further identify the key structural features necessary for the formation of hydrogels and establish structure–functional relationships (SFRs). In this study, we explored the influence of differences in the aromatic cap, amino acid side chains, and cationic groups on both hydrogelation processes and physical properties (Figure 2). The investigation focused on parameters including gelation time, mechanical properties, secondary structure, and fibre morphologies of the resulting hydrogels. Various techniques such as circular dichroism (CD) spectroscopy, atomic force microscopy (AFM), and rheology were employed to establish the SFR of the ultra-short cationic hydrogels reported in this study. Furthermore, it was important to understand if a structure–activity relationship between hydrogelating compounds and their antibacterial activity could be identified. Hence, hydrogelating compounds were tested for their antibacterial properties utilising a contact–activity study and their MICs were measured.

## 2. Results and Discussion

### 2.1. Synthesis of Short Cationic Peptide-Based Hydrogelators ***1***–***12***

#### 2.1.1. Modification A

**Scheme 1:**
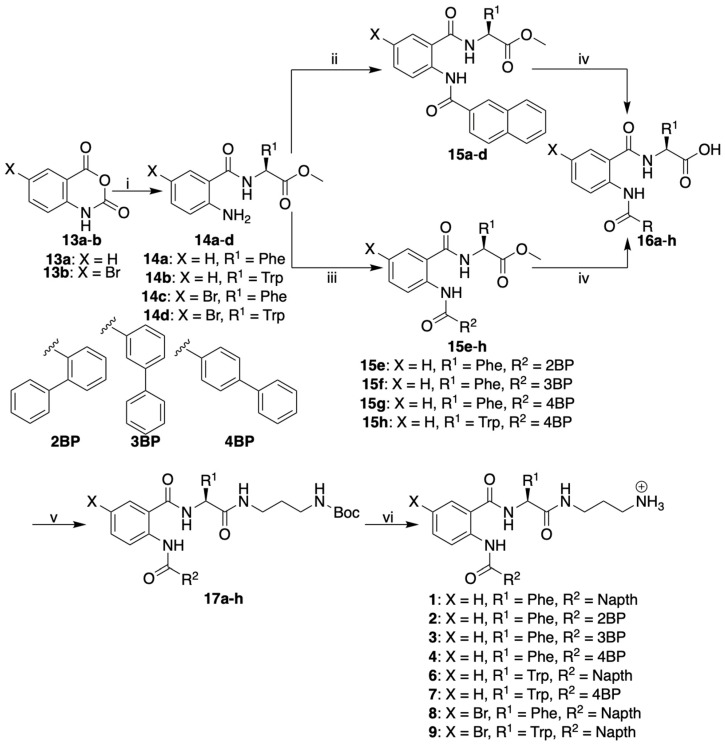
General synthetic scheme for peptide-mimics **1**–**4** and **6**–**9**. Reagents and conditions: (i) Phenylalanine methyl ester or tryptophan methyl ester (1.1 equiv), ACN, reflux, 16 h. (ii) Naphthoyl chloride (2.0 equiv), TEA (3.0 equiv), anh. THF, 16 h. (iii) 2/3/4-biphenyl carbonyl chloride (1.3 equiv), DIPEA (3.0 equiv), 4-DMAP (0.1 equiv), anh. THF, 4 h. (iv) LiOH (3.0 equiv), THF:MeOH:H_2_O (10:5:2 ratio), 18 h. (v) *N*-Boc 1,3-dipropylamine (1.1 equiv), HOBt (1.2 equiv), EDC.HCl (1.2 equiv), DIPEA (2.0 equiv), anh. DMF, 18 h. (vi) TFA (2.0 equiv), DCM, 2 h.

To measure the effect of introducing flexibility within the aromatic cap, the naphthoyl group of **1** was substituted for the 2′, 3′, and 4′-biphenyl aromatic caps, generating peptide-mimics **2**–**4**. These compounds were synthesised utilising a previously reported method [31] with good yields (55–71%) (Scheme 1). Biphenyl **4** was employed to measure the differences in hydrogelation following an increase in one rotational bond in comparison to hydrogelator **1**. Furthermore, the differences in structural isomers **2** and **3** were utilised to investigate the significance of symmetry in forming hydrogels.

**Scheme 2:**
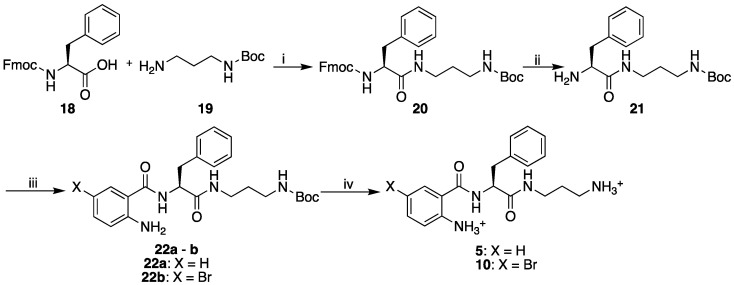
Reagents and conditions: (i) HATU (1.2 equiv), DIPEA (2 equiv), DMF, 0 °C, 1 h; (ii) piperidine (2 equiv), DMF, rt, 12 h; (iii) **22a**, isatoic anhydride **13a** (1.1 equiv) or **22b**, 5-bromo isatoic anhydride **13b** (1.1 equiv), ACN, reflux, 16 h; (iv) TFA (5 equiv), DCM, rt. 2 h.

The aromatic cap of the peptide-mimics has been reported to be important for their hydrogelation, through the formation of π-π stacking interactions [46]. To measure the impact of the aromatic cap within a hydrogelating peptide-mimic, an analogue without an aromatic cap was synthesised (Scheme 2) [29,30]. These peptides were synthesised in a facile manner, yielding 23% over four steps. Here, *N*-terminal Fmoc-protected phenylalanine **18** underwent an amide coupling reaction catalysed by HATU with *N*-Boc-1,3-diaminopropane (**19**). The Fmoc group in **20** was then removed using piperidine, followed by the reaction of primary amine **21** driving a ring-opening reaction through the nucleophilic attack on isatoic anhydrides **13a**–**b**. Following the generation of the anthranilamides **22a**–**b**, the deprotection of the Boc group generated **5** and **10**, peptide-mimicking analogues containing no aromatic cap. The role of the aromatic cap in hydrogelation was investigated (Modification A) [30,31].

#### 2.1.2. Modification B

Kuppusamy and coworkers utilised tryptophan as the amino acid, showing good activity against both *Staphylococcus aureus* (*S. aureus*) and *Escherichia coli* (*E. coli*) [43,47]. Hence, it was of interest to explore the influence of the indole group on hydrogelation on this scaffold. The compounds incorporating the substitution of a benzene ring for an indole group were synthesised with a naphthyl (**6**) and a 4′ biphenyl aromatic cap (**7**) in similar yields to the other compounds synthesised as shown in Scheme 1.

#### 2.1.3. Modification C

In addition to the antibacterial effects resulting from the incorporation of tryptophan in structurally similar compounds, the addition of a bromine substituent in the 5′-position of isatoic anhydride has been shown to improve antibacterial activity in comparison to the unsubstituted analogue [43,47]. Hence 5′-substituted bromine analogues were synthesised to measure their hydrogelating capabilities. Peptide-mimics **8**, **9**, and **10** containing a 5′-bromo group were synthesised utilising the routes shown in Scheme 1 and Scheme 2.

#### 2.1.4. Modification D

**Scheme 3:**
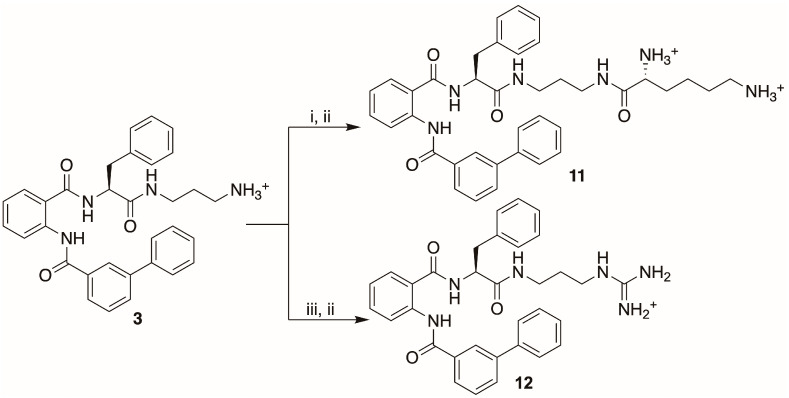
Synthetic scheme for the synthesis of lysine- and guanidine-coupled **11** and **12**, respectively. Reactions and conditions: (i) Boc-Lys(Boc)-OSu (2.0 equiv), DIPEA (4 equiv), anh. DMF, 18 h. (ii) TFA (4 equiv), DCM, 2 h. (iii) **4** (1.0 equiv), *N*,*N*’-Di-Boc-1H-pyrazole-1-carboxamidine (1.1 equiv), TEA (2 equiv), anh. DCM, 18 h.

Cationic ammonium groups have a dual function of improving the water solubility of peptide-mimics for hydrogelation, as well as increasing the selectivity and activity towards the negatively charged bacterial cell membranes over mammalian cells [48]. Guanidinium and primary amines of lysine groups have previously been utilised to improve these attributes, while reducing their toxicity [30,43,49,50,51,52]. This effect was observed for antimicrobial compounds in solution, as well as hydrogels. Hence, compound **3** was reacted with either Boc-Lys(Boc)-OSu or *N*,*N*’-Di-Boc-1H-pyrazole-1-carboxamidine to introduce a Boc-protected lysine or guanidine group (Scheme 3). Deprotection utilising TFA subsequently yielded cationic compounds **11** and **12**.

### 2.2. Hydrogelation Studies

#### 2.2.1. Identification of Hydrogelating Compounds

The hydrogelation testing was initially carried out qualitatively using the vial inversion test [30]. Here, solutions of peptide-mimics were triggered to form 1% *w*/*v* gels by mild heating, utilising two different methods: dissolving in MilliQ (MQ) water or in MQ water containing 5 molar equiv. of sodium chloride. Previously identified naphthoyl **1** [30], as well as novel biphenyls **3**, **4**, and **7** were initially identified to form self-supporting hydrogels within 1 min–3 h, measured by the vial inversion test. No hydrogels were formed without the presence of sodium chloride, suggesting that the presence of salt is essential for the formation of these peptide-mimicking hydrogels. Peptide-mimics **2**, **5**, **8**, **11**, and **12** underwent complete dissolution while heating; however, they did not re-form hydrogels upon cooling. Peptide-mimic **6**, however, bearing a bromine atom and a tryptophan as the amino acid, was too hydrophobic for complete dissolution, and hence lead to no hydrogelation (Table 1). The critical gelation concentrations (CGCs) of compounds that formed hydrogels were also measured (Table 1).

**Figure 3:**
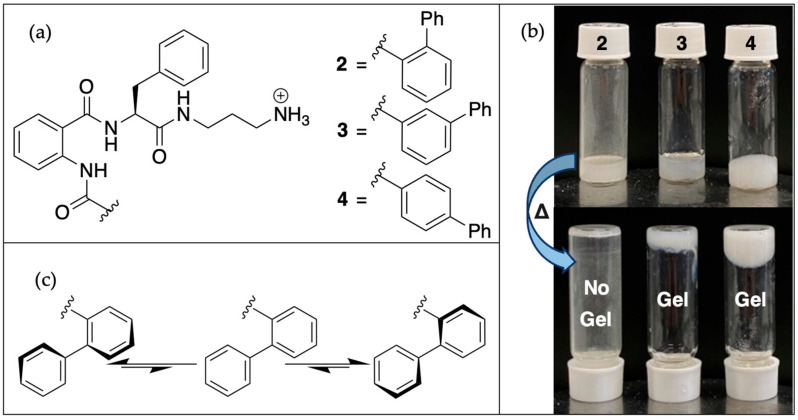
Differences in structural features of the aromatic cap and their gelation properties. (**a**) Structures of peptide-mimics **2**–**4**. (**b**) Differences in hydrogelation between 2 and 4. (**c**) Hypothesised explanation for the inability of peptidomimetic **2** containing a 2′ biphenyl aromatic cap to generate the necessary π-π stacking interactions for hydrogelation.

Newly formed hydrogels **3**, **4**, and **7** were found to have critical gelation concentrations of 0.5% *w*/*v*. Despite the structural similarity, 2′-biphenyl **2** did not form a self-supporting hydrogel (Table 1). These observations suggested that the steric crowding of the 2′-biphenyl group within peptidomimetic **2** may cause the biphenyl rings to prefer a reversible atropisomerised position, reducing the π-π stacking interactions between individual molecules, resulting in the absence of hydrogel formation (Figure 3) [53]. In comparison, increasing the symmetry and reducing the steric crowding within the aromatic cap of hydrogelators containing 3′-biphenyl (**3**) and 4′-biphenyl (**4**) groups resulted in self-supporting hydrogels. Heating compound **5**, which contained no aromatic cap, led to complete dissolution without any gelation observed. The increased solubility of compound **5** in water is thought to be due to two interconnected reasons: Firstly, the removal of the hydrophobic aromatic groups significantly lowered the LogP of **5**, exposing the free amine. Secondly, the amine can act as a base, creating a secondary cationic group resulting in a significantly lower Log P and a reduced ability of **5** to form hydrogelating fibres. Hence, the presence of the aromatic cap is thought to be essential for gel formation within cationic short peptide-based LMWGs.

The effect of amino acid side chains was investigated by substituting phenylalanine with another aromatic amino acid such as tryptophan (Modification B). In general, the introduction of tryptophan decreased the solubility of the hydrogelators, significantly affecting their gelation capability. Hydrogelator **6**, bearing a tryptophan and naphthoyl capping group, was largely insoluble in water, even after heating to a temperature exceeding 90 °C. This peptidomimetic possessed additional π-π stacking interactions, due to the indole functional group, compared with hydrogelator **1** (containing Phe). It is hypothesised that the increased π-π stacking leads to a higher solvation energy barrier required to break the intermolecular bonds of the solid-state molecule, leading to lower water solubility.

In addition, it was found that the flexibility introduced through the aromatic cap increased the solubility enough to reach a concentration critical for hydrogelation. Hydrogelator **7**, containing a 4′-biphenyl aromatic cap and tryptophan, formed an opaque gel at 0.5% *w*/*v*, although small amounts of precipitate were observed at the bottom of the vial. Interestingly, hydrogelator **7** (containing 4 BP and Trp) have a similar LogP value to hydrogelator **6** (containing Napth and Trp), with log P values of 2.43 and 2.74, respectively. This suggests that the flexibility introduced by the 4′-biphenyl aromatic cap compared with the naphthoyl group may reduce the intermolecular π-π stacking interactions of the solid enough to improve the dissolution of **7** [54].

To explore the impact of bromine substituents in the 5′-position of the isatoic anhydride ring, compounds **8**–**10** were synthesised (Modification C). Unfortunately, peptide-mimics **8**–**10** did not form self-supporting hydrogels. Upon heating, bromine-containing compound **8** underwent complete dissolution. Without the π-π stacking interactions generated by the aromatic cap, the peptide-mimics were too hydrophilic for hydrogel formation. Peptide-mimics **9** and **10** underwent complete dissolution; however, the inclusion of the bromine atom compared with structural analogues **1** and **6** lead to no hydrogel formation. It is expected that the bulky bromine atom in the 5′-position of these compounds caused a steric hindrance effect that disrupted the intermolecular π-π stacking interactions important for gelation, a trend also observed in previous studies [30]. While the reduced gelation could be due to the increased polarity of the molecule as a result of the electronegative bromine atom, previous work substituting more electronegative halogens such as fluorine and chlorine observed gel formation, but did not observe it within bromine, suggesting it is more likely to be a steric effect [30].

The incorporation of functional groups such as lysine and guanidine has been reported to typically improve antibacterial activity [36,43,49,55,56]. Hence, compounds incorporating these groups in the anthranilamide scaffold were synthesised and their gelation capacity was measured (Modification D). Unfortunately, the heating and cooling of **11** and **12** showed no hydrogel formation. The reduction in Log P by incorporating these cationic groups made the peptide-mimics too soluble in water, resulting in the π-π stacking interactions being overpowered by the hydrogen bonding and ionic interactions, leading to no observable gelation. This trend has also been observed with other peptide-mimicking compounds containing guanidinium functional groups [30]. Due to the similar Log P values of other lysine and guanidine analogues, the introduction of cationic groups to other hydrogelating compounds was not explored further.

#### 2.2.2. Hydrogel Formation and Rate of Formation

The rate of hydrogelation has varying levels of importance dependant on its respective application. For example, injectable hydrogels that form too quickly may cause a blockage in a syringe needle, while other liquid hydrogels that form gels post application would need to form quickly enough so as not to move from the applicable area while in the liquid state [57]. To investigate the effect of structural modifications on gelation kinetics, the rate of hydrogelation for each hydrogel was measured. Here, compounds were suspended in Milli-Q water containing five equiv. of NaCl, and then heated in a closed vial until complete dissolution. Upon dissolving, the vials were left at room temperature to cool down, undergoing the hydrogelation process. Hence, measuring the time between ceasing heating and the observation of gel formation via the vial inversion test was used as a proxy for the rate of hydrogelation. These compounds demonstrated that the 4-biphenyl compound **4** hydrogelates within ~5 min of heating, while the 3-biphenyl compound **3** required approximately 3 h to gel. This is in comparison to **1**, which gelates in less than 1 min. The large difference in hydrogelation rates between **3** and **1** is thought to be due to the increased flexibility and conformational positions available, as a result of the reduced symmetry of **3**. Here, additional conformational possibilities are explored when heating and cooling, requiring a greater sampling time for the 3′-biphenyl aromatic cap to find the ideal π-π stacking interactions. The reduced number of conformational positions and increased symmetry within **4** in comparison to **3** led to an increased chance of the aromatic cap organising into the correct π-π stacking conformation, providing a potential explanation for the observed relationship between the flexibility within the aromatic cap and the hydrogelation time.

The substitution of phenylalanine for tryptophan in **7** compared with **4** led to gelation within approximately 5 min. These data suggest that despite the slight insolubility observed during gelation, the rate of hydrogelation is largely not affected.

#### 2.2.3. Secondary Structure

Peptide-mimicking hydrogels have been reported to generate secondary structures that can be elucidated through circular dichroism (CD) [30,31]. Amide bonds typically absorb UV light at wavelengths approximately 240 nm–190 nm, and depending on absorbance patterns, can give insight into their secondary structural arrangement.

**Figure 4:**
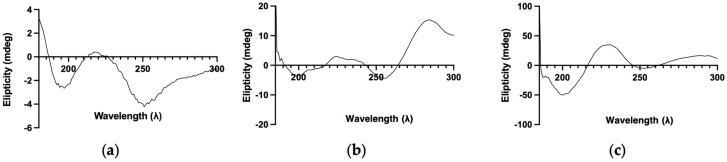
CD spectra of hydrogels **3** (**a**), **4** (**b**), and **7** (**c**).

Despite the structural similarity between **3**, **4**, and **7**, a mixture of secondary structural features was observed. For the CD spectrum of **3**, a minimum at 196 nm (π ⟶ π*) and a maximum at 217 nm (n ⟶ π*) is highly indicative of antiparallel β-sheets [58] (Figure 4) [59]. Meanwhile, the CD of **4** suggests that an α-helix may be forming, due to the two maxima at 225 nm and 233 nm, as well as a minimum at 199 nm [59].

Tryptophan peptide-mimic **7** has one minimum at 199 nm and one maximum at 228 nm (Figure 4). These features within the graph are indicative of random-coiled structures [58].

#### 2.2.4. Mechanical Properties

**Figure 5:**
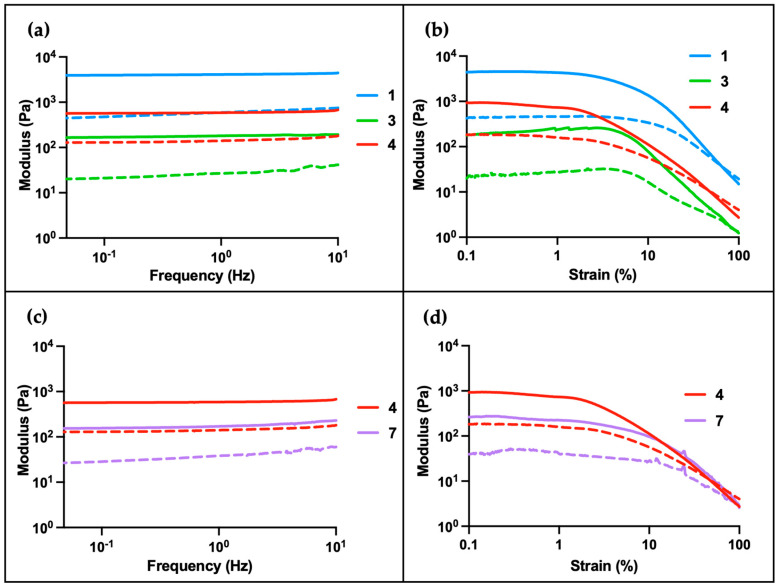
Rheological studies of hydrogelating peptide-mimics: (**a**) Frequency sweep measurement of **1**, **3**, and **4**. (**b**) Strain sweep measurement of **1**, **3**, and **4**. (**c**) Frequency sweep measurement of **4** and **7**. (**d**) Strain sweep measurement of **4** and **7**. Solid lines represent storage modulus (*G*’) and dashed lines represent loss modulus (*G*”).

The mechanical properties of gels are typically measured using rheology. Gels are characterised by their storage modulus (*G*’) being greater than their loss modulus (*G*”) (*G*’ > *G*”). The storage modulus can be used as an indicator of a hydrogel’s stiffness. The larger the *G*’ value, the stiffer the hydrogel. Hydrogels **3**, **4**, and **7** had storage moduli that were significantly larger than their loss moduli within the frequency sweep test (FST), confirming their gel-like nature and supporting the vial inversion test. However, a reduction in stiffness was observed with an increasing number of conformational positions available to the aromatic cap (Figure 5). Here, the mechanical strengths of **1**, **4**, and **3** were lowered from 3.9 kPa to 0.57 kPa and 0.17 kPa. The reduction in hydrogel stiffness may be explained by the increased rotational flexibility within the aromatic cap. The more symmetrical linear packing motifs contain fewer conformational positions, increasing the chance of generating π-π stacking interactions and thereby increasing the mechanical strength of the hydrogel. This is supported by the similar trend observed in the rate of gelation, whereby increasing flexibility within the aromatic cap leads to slower gelation. These observations also resemble features noted in previous studies utilising *meta* and *para* biphenyl groups [31].

Comparing 4′-biphenyl compounds **4** and **7** also showed a significant drop in the mechanical strength of the hydrogel (0.57 kPa vs. 0.15 kPa, respectively). The FST highlights a reduction in the mechanical strength of tryptophan **7** in comparison with phenylalanine **4**. The reduction in stiffness was initially unexpected, as **7** and **4** have the same aromatic cap and **7** also has a fused two-ring aromatic system like naphthyl, suggesting that it can form improved π-π stacking interactions. This, however, may be accounted for by the lower solubility of **7**, as small amounts of precipitate were present, lowering the concentration of the molecules present within the fibres. The insoluble particles of **7** may also hinder hydrogel fibre formation, resulting in a lower mechanical strength.

The strain sweep test (SST) measures the linear viscoelastic region (LVER), which is a good indicator of the resistance to oscillatory strain, a force that has been reported to be applied by cells [60]. Here, longer LVERs suggest an increased resistance to oscillatory strain. SSTs of **1**, **3**, and **4** highlight that increasing the number of rotational conformers within the aromatic cap lengthens the LVER. SSTs of hydrogels **1**, **3**, and **4** indicated LVERs of 1.76 (±0.01%), 3.44 (±0.01%), and 1.97 (±0.03%), respectively. Here, increasing the flexibility within the aromatic cap from naphthoyl to 4′-biphenyl highlights a slight increase in their resistance to strain. However, substitution to the 3′-biphenyl aromatic ring significantly increased the resistance to strain as shown by the lengthening in LVER (Figure 5). These data are also consistent with other LMWGs utilising *meta* and *para* biphenyl groups [31]. The increased flexibility within the 3′-biphenyl aromatic cap may transcend molecule–molecule interactions, and allow for greater flexibility within the fibre, allowing for the greater absorption of strain within the whole network.

When observing the effects of tryptophan in comparison with phenylalanine, the SSTs showed **7** to be less resistant to strain than **4**, exhibiting a shorter LVER (1.08 kPa (±0.03%)) vs. 1.97 kPa (±0.03%), respectively). This may also be a result of the reduced solubility of **7**, as well as the increased rigidity within the fused ring system. Hence, further investigation is necessary to measure the effect of the indole functional group.

These data suggest that tuning the mechanical features of LMWGs to make them stiffer or softer is possible through the substitution of groups that form π-π stacking interactions with different levels of flexibility. Here, it is possible to reduce the stiffness of a hydrogel by reducing the rigidity of the aromatic cap. Furthermore, the introduction of more asymmetric aromatic capping groups within the scaffold could generate even softer gels that are more resistant to oscillatory forces. These findings could be utilised in the future for the design of hydrogels with more specific applications.

#### 2.2.5. Structure Morphology

**Figure 6:**
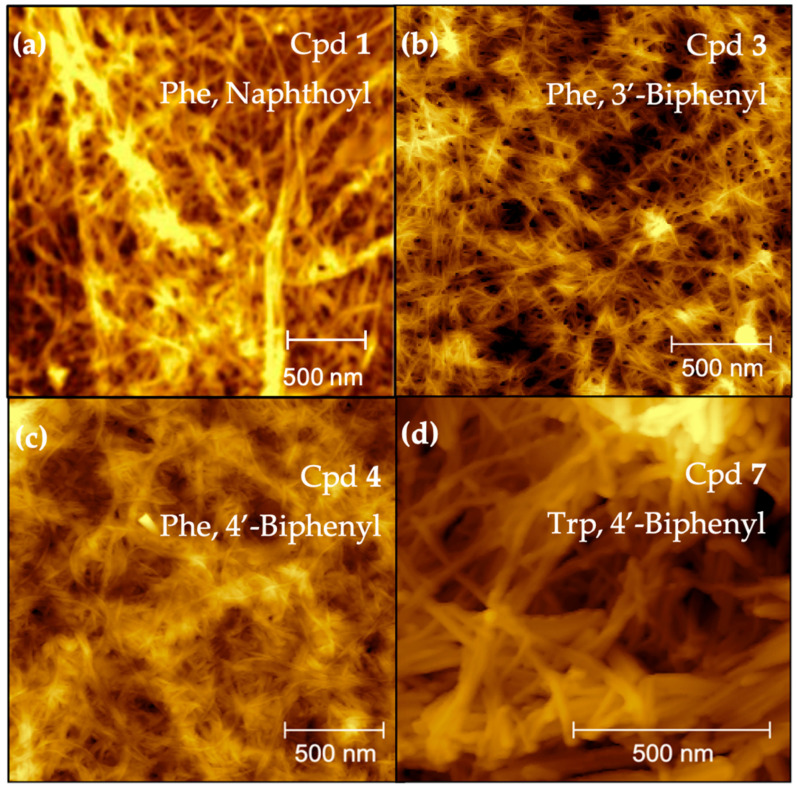
Three-dimensional fibrous network of xerogels from hydrogels **1**, **3**, **4**, and **7**. (**a**) Hydrogel **1** containing a naphthoyl group. Figure adapted with permission from [30]. (**b**) Hydrogel **3** containing a 3′-biphenyl aromatic cap. (**c**) Hydrogel **4** containing a 4′-biphenyl aromatic cap. (**d**) Hydrogel **7** with a tryptophan amino acid containing a 4′-biphenyl aromatic cap.

Differences in features such as physical strength may be observed within the fibre morphology. To understand these differences, xerogels (air-dried hydrogels) were formed and imaged using atomic force microscopy (AFM). In comparison to **1**, xerogels **3**, **4**, and **7** displayed large differences in fibre diameter and types of fibre distribution. The xerogels **3** and **4** displayed a network of nano fibres with many junction zones and differing diameters (Figure 6, Table 2). The 3′-biphenyl **3** and 4′-biphenyl hydrogel **4** both displayed long linear fibres with smaller branching groups that diverge from these linear fibres. They differ however in the way that **3** appears to clump together densely in interconnected nodes, while 4′-biphenyl **4** appears to have more homogenous and consistent branching to create the appearance of a bristle-like network (Figure 6). This more homogenous-like density of **4** with main fibres that generate smaller branching fibres may provide evidence for the larger *G*’ values observed within the rheological studies, as when force is applied to the hydrogel, it is spread more evenly throughout the fibres. In contrast, the heterogenous node-like nature of **3** creates large areas of empty space, which may be responsible for the increased length of the LVER observed in the SSTs (Figure 5). When oscillatory strain was applied to the hydrogels, the dense interconnected nodes may have been able to utilise this space for the absorption of the additional force, and this could account for their additional flexibility. Furthermore, compared with **1**, all three xerogels have significantly thinner fibres, which may explain their respective strengths and CGCs.

The xerogel of tryptophan peptide-mimic **7** was also formed for AFM imaging (Figure 6). The fibres appeared to be long and thin, occasionally bundling together. However, there was a lack of an organised pattern in comparison to images of **1**, **3**, and **4**. This lack of ordered structure may account for the reduced strength observed by rheology in comparison with the phenylalanine hydrogels (Figure 4 and Figure 6).

### 2.3. Antibacterial Activity

*S. aureus* and *E. coli* are bacteria that make up a large percentage of soft tissue infections [61,62]. Hence, synthesised hydrogels were tested against these strains of bacteria for antibacterial activity. While the mechanism of action for cationic short peptide-mimicking molecules against bacterial cells is not fully clear, it is commonly reported that the cationic charge is important for activity and selectivity towards bacterial cells over mammalian cells due to the negative phospholipid layer within the bacterial cell membrane [43,44,47,49,56].

Furthermore, supramolecular nanofibres have been shown to enhance antibacterial activity, likely as a result of increased local cationic density along the fibril surface [63,64,65,66]. Previous work by Aldilla et al. highlighted that the active compound (**1**) exerted significantly less antibacterial activity as a free compound in solution at the same concentration in comparison to **1** as a gel. Due to the structural similarity of these compounds, only compounds that formed hydrogels were tested for antibacterial activity [30].

To measure the antibacterial activity of the three novel peptide-mimicking hydrogels, they were initially exposed to a contact–activity study. Here, hydrogels **3**, **4**, and **7** were formed in sterile vials, followed by layering inoculated *S. aureus* or *E. coli* on top of the hydrogels. Following incubation at 37 °C overnight, the aqueous bacterial layer was plated and incubated. There was no growth of either bacterial strain for all hydrogels, suggesting that these hydrogels were active against both *S. aureus* and *E. coli*, while the control plate observed intensive bacterial growth.

**Figure 7:**
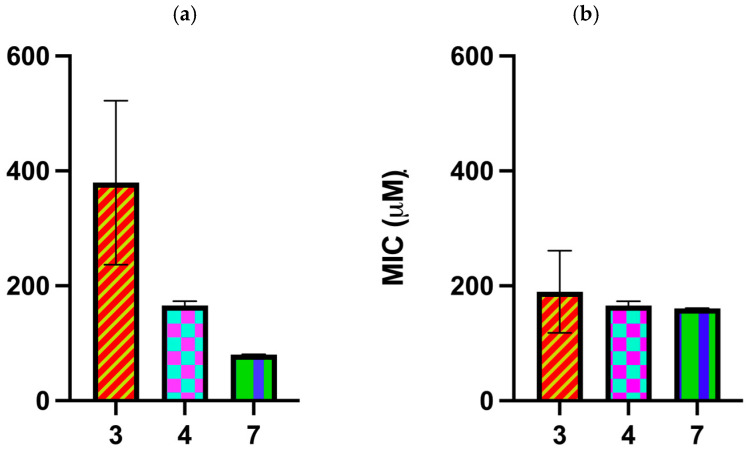
Minimum inhibitory concentrations of peptide-mimicking cationic hydrogels **3**, **4**, and **7** following release of fibres into water against (**a**) *S. aureus* 38 and (**b**) *E. coli* K12.

To further quantify the antibacterial activity, these hydrogels also underwent a minimum inhibitory concentration (MIC) study. The gels were incubated at 37 °C overnight while layered with an equal volume of water, allowing for gel fibres to diffuse into the water. The volume of aqueous solution necessary for MIC testing was then taken, and the remainder of the aqueous solution was used to measure the concentration of the hydrogel fibres against a standard curve of known concentrations using UV–vis spectrophotometry. Here, Beer’s law was used to calculate the MIC from the absorbance of the aqeous solutions. The MICs of **3**, **4**, and **7** against *S. aureus* were 189 µM, 166 µM, and 161 µM, and against *E. coli* were 379 µM, 166 µM, and 80 µM, respectively (Figure 7). These data suggest that the anthranilamide scaffold is effective at killing both types of bacteria while in the hydrogel state. Peptide-mimic **7**, despite the lack of ordered secondary structures, exhibited the best activity against both genera, indicating that the indole group of tryptophan may be important for activity as a gel or a molecule by itself, which is supported by previous studies [43,47,49]. Furthermore, hydrogel **4** had better activity in comparison to hydrogel **3** towards *E. coli*, but only a minimal difference was observed in activity against *S. aureus*. These compounds had similar levels of activity to that previously reported for **1** [30]. Overall, these findings demonstate the ability of hydrogels **3**, **4**, and **7** to exert antibacterial effects as both hydrogels or as eluted fibres in an aqueous solution.

## 3. Materials and Methods

### 3.1. General Notes—Synthesis

All chemical reagents were purchased from commercial sources (Combi-Blocks (San Diego, CA, USA), Chem Impex (Wood Dale, IL, USA), Sigma Aldrich (Saint Louis, MO, USA), Chem Supply (Bedford, Australia), and Ambeed (Buffalo Grove, IL, USA)) and used without further purification. Solvents were commercial and used as obtained. Reactions were performed using oven-dried glassware under an atmosphere of argon and in anhydrous conditions (as required). Room temperature refers to the ambient temperature. Yields refer to chromatographically and spectroscopically pure compounds, unless otherwise stated. Reactions were monitored by thin layer chromatography (TLC) plates pre-coated with Merck (Boston, MA, USA) silica gel 60 F254. Visualisation was accomplished with UV light and/or a ninhydrin staining solution in n-butanol, as required. Flash chromatography and silica pipette plugs were performed under positive air pressure using Silica (Perth, Australia) Gel 60 of 230–400 mesh (40–63 µm) and using Grace Davison (Columbia, MD, USA) LC60A 6 µm for reverse phase chromatography. Infrared spectra were recorded using a Cary 630 ATR spectrophotometer (Agilent, Santa Clara, CA, USA). Melting points were obtained using an OptiMelt (Thebarton, Australia) melting point apparatus and are uncorrected. High-resolution mass spectrometry was performed by the Bioanalytical (Melbourne, Australia) Mass Spectrometry facility, UNSW. Proton and carbon NMR spectra were recorded in the solvents specified using a Bruker (Billerica, MA, USA) DPX 300 or a Bruker Avance 400 spectrometer, as designated. Chemical shifts (∂) are quoted in parts per million (ppm), to the nearest 0.01 ppm, and internally referenced relative to the solvent nuclei. ^1^HNMR spectral data are reported as follows: [chemical shift in ppm; multiplicity in br, broad; s, singlet; d, doublet; t, triplet; q, quartet; quint, quintet; sext, sextet; sept, septet; m, multiplet; or as a combination of these (e.g., dd, dt, etc.)]; coupling constant (*J*) in hertz, integration, proton count, and assignment.

#### 3.1.1. General Procedure 1 for Synthesis of **14a**–**d** and **22a**–**b**

Commercially available L-phenylalanine methyl ester hydrochloride salt or L-tryptophan methyl ester hydrochloride salt (1.1 equiv) was dissolved in saturated sodium bicarbonate solution and extracted with ethyl acetate. The organic ethyl acetate solution was evaporated down under vacuum, resulting in the free amines. Here, the phenylalanine- or tryptophan-free amines were subsequently added to isatoic anhydride or 5′-bromo isatoic anhydride (1.0 equiv), followed by the addition of acetonitrile (minimum volume to create a free-flowing suspension). The reaction mixture was heated to reflux and stirred for 18 h. After completion, the cloudy reaction mixture was then evaporated under reduced pressure to remove the acetonitrile. The resulting white precipitate was washed with diethyl ether, filtered, and dried to give compounds **14a**–**d** or **22a**–**b** as white solids with 57–83% yields.

#### 3.1.2. General Procedure 2 for Synthesis of **15a**–**d**

Anthranilamides **14a**–**d** were suspended in anhydrous THF under an argon atmosphere, followed by the addition of triethylamine. After stirring for 10 min, naphthoyl chloride was slowly added and the mixture stirred for 16 h. Following the completion of the reaction, the cloudy reaction mixture was evaporated under reduced pressure to remove ethyl acetate. The crude mixture was then extracted with ethyl acetate and the extract washed with water (1×), hydrochloric acid (1×), sodium bicarbonate (3×), and brine (1×). The organic layer was then dried over magnesium sulphate and reduced under vacuum. The resulting crude mixture was purified using flash chromatography with hexane/ethyl acetate to produce **15a**–**d** as a white solid with a 65–89% yield.

#### 3.1.3. General Procedure 3 for Synthesis of **15e**–**h**

[1,1′-Biphenyl]-2-carboxylic acid, [1,1′-biphenyl]-3-carboxylic acid, or [1,1′-biphenyl]-4-carboxylic acid (1.3 equiv) were initially dissolved in anhydrous DCM under an argon atmosphere. The mixture was then cooled to 0 °C while stirring for 5 min, followed by the addition of oxalyl chloride (1.5 equiv). Next, a catalytic amount of dimethylformamide (0.25 equiv) was added dropwise, with a venting needle to allow the removal of the carbon dioxide and carbon monoxide gas produced. The reaction was left to stir at room temperature for 12 h. Following the complete conversion of the carboxylic acid to acyl chloride, the reaction mixture was concentrated by blowing with nitrogen gas, giving a yellow oil. The yellow oil was then dissolved in anhydrous THF, under a new argon atmosphere, followed by the addition of pyridine (3 equiv) and catalytic amounts of 4-dimethylaminopyridine (0.1 equiv). After stirring for 10 min, anthranilamide **14a**–**b** was added slowly. The reaction was stirred for 4 h. The crude mixture was then concentrated under reduced pressure and extracted with ethyl acetate. The resulting organic layer was washed with water (1×), hydrochloric acid (1×), sodium bicarbonate (3×), and brine (1×). The organic layer was then dried over magnesium sulphate and concentrated under reduced pressure. The resulting crude mixture was purified using flash chromatography with hexane/ethyl acetate to produce **15e**–**h** as white solids with a 54–65% yield.

#### 3.1.4. General Procedure 4 for Synthesis of **16a**–**h**

Methyl ester-protected intermediates **15a**–**h** were dissolved in THF:MeOH:H_2_O in a volume ratio of 10:5:2. Lithium hydroxide (3 equiv) was then added to the reaction mixture which was stirred for 18 h. The reaction mixture was diluted with Milli-Q water and then washed with diethyl ether (3×). The aqueous phase was acidified to pH 3–4 and was then extracted with ethyl acetate (2×). The resulting organic extract was dried over magnesium sulphate and concentrated under reduced pressure to produce **16a**–**h** as white solids with a 70–81% yield.

#### 3.1.5. General Procedure 5 for Synthesis of **17a**–**h**

Anthranilamides **16a**–**h** with a carboxylic acid (1 equiv) were dissolved in anhydrous DMF under an argon atmosphere and cooled down to 0 °C. HOBt (1.2 equiv) was added and the mixture stirred for 15 min. N-Boc-1,3-diaminopropane **19** (1.1 equiv) was then added, followed by EDC.HCl (1.2 equiv) and finally DIPEA (2 equiv). The cloudy reaction mixture was warmed to room temperature and stirred for 18 h. The resulting crude reaction mixture was concentrated under reduced pressure, followed by washing with 0.5 M HCl (1×), saturated sodium bicarbonate (3×), and brine (1×) and the extract was dried over magnesium sulphate and concentrated under reduced pressure. The resulting solid was then stirred in diethyl ether (0.1 g·mL^−1^) followed by decanting 3×. The remaining mixture was filtered and purified via column chromatography to give pure white solids with 50–72% yields.

#### 3.1.6. General Procedure 6 for Synthesis of **1**–**12**

Boc-protected **17a**–**h**, **22a**–**b**, **23**, and **24** were each suspended in anhydrous DCM (1 equiv) under argon atmosphere. The clear solution was then cooled down to 0 °C for 10 min, followed by the addition of TFA (3 equiv). The reaction mixture was warmed to room temperature and stirred for 2–18 h. After complete consumption of the starting material, as indicated by TLC, the reaction mixture was concentrated by blowing down with nitrogen gas, giving a brown gummy solid. The crude reaction mixture was stirred in diethyl ether for 10 min, followed by decanting or filtering, providing **1**–**12** as white or brown solids.

### 3.2. Hydrogel Characterisation

#### 3.2.1. Preparation of Hydrogels

Peptide-mimics **1**–**12** were added to a vial with an internal diameter (i.d.) of 10 mm. Subsequently, 1 M NaCl (5 equiv) was added, followed by a sufficient amount of water to make up a 1% (*w*/*v*) suspension or solution. The resulting suspensions or solutions of **1**–**12** were slowly heated until all the solid either completely dissolved or the water began to boil. The resulting suspension or solution was left at room temperature without disturbance overnight. The vials were then inverted to confirm hydrogel formation. CGC, represented as a percentage, is the minimum weight of hydrogelator required to form a self-supporting hydrogel divided by the total volume.

#### 3.2.2. Circular Dichroism (CD) Spectroscopy

Hydrogelating peptide-mimics **3**, **4**, and **7** were prepared at 1% *w*/*v* and were diluted 8× in Milli-Q water before being transferred into a 0.2 mm path length cuvette. The spectra were collected using a ChriascanPlus CD spectrometer (Applied Photophysic Leatherhead, UK) with scanning wavelengths of 180–500 nm with a bandwidth of 1 nm, 0.6 s per point, and step of 1 nm. Each experiment was performed in triplicate and the results were averaged into a single point.

#### 3.2.3. Rheology Measurements

The mechanical properties of cationic hydrogels were assessed using an Anton Paar (Graz, Austria) MCR 302 or Anton Paar MCR 302e rheometer with a 25 mm stainless parallel plate configuration. All rheological data points were obtained at 37 °C to replicate body temperature for clinical application. Initially, 1 mL of the hydrogels was prepared from **1**, **3**, **4**, and **7** in glass vials at 1% *w*/*v*. These vials were warmed using a heat gun to transform the hydrogel to the solution phase. Prior to gelation, 400 µL of the resulting solutions was cast onto the rheometer plate. The other plate was lowered to the measuring position (0.5 mm) and the hydrogel was allowed to stand for 3 h for the gel to form. Initially an SST was performed at a frequency of 1 Hz using 0.1% strain to 100% strain. Subsequently an FST was conducted at a fixed strain of 0.1%, which is within the LVER of all hydrogels, and frequency ranging from 10 to 0.01 Hz. The rheology data were shown as the average of three repeats for each data point.

#### 3.2.4. Atomic Force Microscopy (AFM)

Hydrogels **1**, **3**, **4**, and **7** were prepared as previously described, at 2× and 4× below their CGC. These anthranilamide mixtures were heated using a heat gun, and one drop of the anthranilamide solutions was cast onto a mica substrate. Using a glass slide, each droplet was carefully spread and was left to dry overnight at room temperature in a fume hood under laminar flow to ensure dryness before imaging. Imaging was performed using a Bruker Multimode 8 Atomic Force Microscope in Scanasyst Air (PeakForce Tappings) mode, which is based on tapping mode AFM. To prevent damage to soft samples, the imaging parameters were constantly optimised through the force curves that were collected. Bruker Scanasyst Air probes were used, with a spring constant of 0.4 N·m^−1^ and a tip radius of 2 nm.

### 3.3. Antibacterial Assays

#### 3.3.1. General Bacterial Preparation Procedure

A single colony of *S. aureus* 38 or *E. coli* K12 was grown overnight in Mueller Hinton Broth (MHB) at 37 °C. The resulting suspension was centrifuged and resuspended in the same volume of MHB. The optical density (OD) of the resulting bacteria culture was adjusted to 0.1 at 600 nm in MHB (10^8^ CFUs/mL) which was further diluted to 10^5^ CFUs/mL for contact–activity study, or 10^6^ CFUs/mL for MIC study.

#### 3.3.2. Bacterial Loading on Hydrogels

Bacterial solutions (1 mL) which contained 10^5^ CFUs/mL were layered on top of 1 mL of 1% *w*/*v* hydrogels **4**, **5**, and **7**, by gently dispensing the solution down the side wall to avoid physical fractures to the gel, making up a total of 2 mL in a glass sterile vial, and 1 mL of a 0.079 mol·L^−1^ solution of NaCl was used to measure any effect of 5 equiv. of NaCl used within the hydrogel solutions. Bacterial solutions (1 mL) were added to 1 mL of Milli-Q water as a negative control. After being incubated at 37 °C for 18 h, 20 µL of bacterial solution in each vial was taken and carefully transferred into nutrient agar plates and incubated for another 18 h. The following day, bacterial growth inhibition was quantified using the viable count method. The experiment was performed in triplicate.

#### 3.3.3. Hydrogel Fibril Release and MIC Assay

Hydrogels **4**, **5**, and **7** were made up at 1% *w*/*v*, with a final volume of 1 mL, as described above. Next, 1 mL of Milli-Q water was then layered on top of the hydrogel as described above and left to stand overnight at room temperature for 21 h. From the aqueous layer, released fibres underwent serial dilution in 96-well plates, making up 100 µL. Subsequently, 100 µL bacterial solutions (10^6^ CFUs/mL) were added to each well containing samples. A blank control was used containing 100 µL of bacteria solution and 100 µL of MHB, making up a total volume of 200 µL. The plate was then incubated at 37 °C for 24 h. The following day, 20 µL from each well was transferred onto agar plates followed by incubation at 37 °C for another 24 h. MIC values were determined as the lowest concentration that inhibited 90% of *S*. *aureus* and *E. coli* growth on the agar plate [67].

### 3.4. Analytical Data

The analytical data for intermediates **14a**–**d**, **15a**–**d**, **16a**–**d**, and **17a** and final compound **1** were already mentioned in previous publications [30,49].

Methyl (2-([1,1′-biphenyl]-2-carboxamido)benzoyl)-*L*-phenylalaninate (**15e**).

Synthesised according to general procedure 3 from compound **14a**. ^1^H NMR (400 MHz, DMSO) δ 3.06 (dd, *J* = 13.8, 10.1 Hz, 1H), 3.17 (dd, *J* = 13.8, 5.2 Hz, 1H), 3.62 (s, 3H), 4.62 (ddd, *J* = 10.1, 7.7, 5.2 Hz, 1H), 7.11–7.22 (m, 2H), 7.24–7.30 (m, 5H), 7.32 (dt, *J* = 6.8, 1.3 Hz, 2H), 7.34–7.38 (m, 2H), 7.44–7.53 (m, 3H), 7.53–7.63 (m, 2H), 7.67 (dd, *J* = 7.9, 1.5 Hz, 1H), 8.34 (d, *J* = 8.3 Hz, 1H), 9.08 (d, *J* = 7.7 Hz, 1H), 11.16 (s, 1H). ^13^C NMR (101 MHz, DMSO) δ 171.71, 168.19, 167.30, 139.80, 139.51, 138.75, 137.45, 136.54, 132.31, 130.84, 130.44, 129.08, 128.30, 128.27, 128.12, 127.61, 127.54, 127.36, 127.16, 126.59, 122.95, 120.31, 120.17, 54.10, 52.06, 40.15, 39.94, 39.73, 39.52, 39.31, 39.10, 38.89, 36.12. HRMS (ESI): calcd for C_30_H_26_N_2_O_4_ + H: 479.1878 found 479.1965.

Methyl (2-([1,1′-biphenyl]-3-carboxamido)benzoyl)-*L*-phenylalaninate (**15f**).

Synthesised according to general procedure 3 from compound **14a**. ^1^H NMR (400 MHz, DMSO) δ 3.11 (ddd, *J* = 15.2, 10.1, 2.1 Hz, 1H), 3.23 (dd, *J* = 13.9, 5.0 Hz, 1H), 3.60 (s, 3H), 4.71–4.83 (m, 1H), 7.08–7.14 (m, 1H), 7.17–7.27 (m, 3H), 7.26–7.32 (m, 2H), 7.40–7.46 (m, 1H), 7.52 (ddd, *J* = 7.8, 6.7, 1.4 Hz, 2H), 7.59 (ddd, *J* = 8.6, 7.3, 1.5 Hz, 1H), 7.67 (td, *J* = 7.7, 1.7 Hz, 1H), 7.73 (t, *J* = 1.1 Hz, 1H), 7.74–7.80 (m, 2H), 7.83 (ddq, *J* = 7.9, 2.1, 1.0 Hz, 1H), 7.90–7.96 (m, 1H), 8.12 (dt, *J* = 3.1, 1.8 Hz, 1H), 8.61 (ddt, *J* = 8.5, 2.3, 1.3 Hz, 1H), 9.28 (dd, *J* = 7.3, 2.0 Hz, 1H), 12.04–12.15 (m, 1H). ^13^C NMR (101 MHz, DMSO) δ 172.12, 169.31, 164.79, 141.26, 139.72, 139.54, 137.94, 135.67, 133.07, 130.78, 130.17, 129.62, 129.53, 128.89, 128.67, 128.50, 127.29, 126.98, 126.38, 125.64, 123.47, 120.82, 120.62, 54.66, 52.54, 40.61, 40.40, 40.19, 39.98, 39.77, 39.56, 39.35, 36.54. HRMS (ESI): calcd for C_30_H_26_N_2_O_4_ + H: 479.198 found 479.1966.

Methyl (2-([1,1′-biphenyl]-4-carboxamido)benzoyl)-*L*-phenylalaninate (**15g**).

Synthesised according to general procedure 3 from compound **14a**. ^1^H NMR (400 MHz, DMSO) δ 3.12 (dd, *J* = 13.8, 10.5 Hz, 1H), 3.25 (dd, *J* = 13.8, 5.0 Hz, 1H), 3.67 (s, 3H), 4.81 (ddd, *J* = 10.4, 7.8, 4.9 Hz, 1H), 7.08–7.18 (m, 1H), 7.25 (dt, *J* = 8.5, 7.1 Hz, 3H), 7.29–7.36 (m, 2H), 7.39–7.51 (m, 1H), 7.48–7.56 (m, 2H), 7.60 (ddd, *J* = 8.5, 7.4, 1.5 Hz, 1H), 7.78 (ddd, *J* = 8.1, 4.0, 1.3 Hz, 3H), 7.89 (d, *J* = 8.6 Hz, 2H), 7.94 (d, *J* = 8.5 Hz, 2H), 8.64 (dd, *J* = 8.4, 1.2 Hz, 1H), 9.29 (d, *J* = 7.9 Hz, 1H), 12.06 (s, 1H). ^13^C NMR (101 MHz, DMSO) δ 172.14, 169.30, 164.51, 144.11, 139.65, 139.36, 137.95, 133.60, 133.08, 129.56, 128.86, 128.76, 128.70, 128.07, 127.63, 127.45, 127.02, 123.37, 120.79, 120.36, 54.58, 52.64, 40.61, 40.40, 40.19, 39.98, 39.77, 39.56, 39.35, 36.59. HRMS (ESI): calcd for C_30_H_26_N_2_O_4_ + Na: 501.1798 found 501.1787.

Methyl (2-([1,1′-biphenyl]-4-carboxamido)benzoyl)-*L*-tryptophanate (**15h**).

Synthesised according to general procedure 3 from compound **14b**.

^1^H NMR (400 MHz, DMSO) δ 3.29 (dd, *J* = 14.7, 9.4 Hz, 1H), 3.67 (s, 3H), 4.79 (ddd, *J* = 9.2, 7.4, 5.2 Hz, 1H), 6.98 (ddd, *J* = 8.0, 7.0, 1.2 Hz, 1H), 7.06 (ddd, *J* = 8.1, 7.0, 1.3 Hz, 1H), 7.18–7.27 (m, 2H), 7.32 (dd, *J* = 7.9, 1.1 Hz, 1H), 7.39–7.48 (m, 1H), 7.48–7.56 (m, 2H), 7.56–7.64 (m, 2H), 7.77 (dt, *J* = 6.3, 1.3 Hz, 2H), 7.81–7.90 (m, 3H), 7.90–7.97 (m, 2H), 8.65 (dd, *J* = 8.4, 1.2 Hz, 1H), 9.25 (d, *J* = 7.5 Hz, 1H), 10.86 (d, *J* = 2.4 Hz, 1H), 12.16 (s, 1H). ^13^C NMR (101 MHz, DMSO) δ 172.51, 169.40, 164.54, 144.05, 139.76, 139.37, 136.60, 133.63, 133.07, 129.56, 129.01, 128.74, 128.06, 127.64, 127.52, 127.43, 124.21, 123.30, 121.49, 120.76, 120.31, 118.92, 118.48, 111.98, 110.30, 54.37, 52.59, 40.62, 40.41, 40.20, 39.99, 39.78, 39.57, 39.36, 26.91. HRMS (ESI): calcd for C_32_H_27_N_3_O_4_ + Na: 540.1898 found 540.1895.

(2-([1,1′-biphenyl]-2-carboxamido)benzoyl)-*L*-phenylalanine (**16e**).

Synthesised according to general procedure 4 from compound **15e**. ^1^H NMR (400 MHz, DMSO) δ 3.02–3.18 (m, 1H), 3.25 (dd, *J* = 13.9, 4.3 Hz, 1H), 4.74 (ddd, *J* = 11.9, 8.2, 4.2 Hz, 1H), 7.11 (t, *J* = 7.4 Hz, 1H), 7.23 (q, *J* = 8.1 Hz, 3H), 7.33 (d, *J* = 7.6 Hz, 2H), 7.43 (t, *J* = 7.4 Hz, 1H), 7.52 (t, *J* = 7.6 Hz, 2H), 7.58 (t, *J* = 7.9 Hz, 1H), 7.79 (t, *J* = 8.2 Hz, 3H), 7.91 (q, *J* = 8.1 Hz, 4H), 8.64 (d, *J* = 8.4 Hz, 1H), 9.15 (d, *J* = 8.2 Hz, 1H), 12.18 (s, 1H), 12.41 (s, 1H). ^13^C NMR (101 MHz, DMSO) δ 172.72, 168.12, 167.28, 139.83, 139.55, 138.76, 137.94, 136.58, 132.22, 130.46, 130.43, 129.08, 128.30, 128.25, 128.23, 127.65, 127.50, 127.37, 126.48, 122.91, 120.26, 120.21, 54.07, 40.16, 39.95, 39.74, 39.53, 39.33, 39.12, 38.91, 36.15. HRMS (ESI): calcd for C_29_H_24_N_2_O_4_ + H: 465.1778 found 465.1810.

(2-([1,1′-biphenyl]-3-carboxamido)benzoyl)-*L*-phenylalanine (**16f**).

Synthesised according to general procedure 4 from compound **15f**. ^1^H NMR (400 MHz, DMSO) δ 3.06 (dd, *J* = 13.7, 10.1 Hz, 1H), 3.28 (dd, *J* = 13.8, 4.2 Hz, 2H), 4.60–4.69 (m, 1H), 7.03 (t, *J* = 7.4 Hz, 1H), 7.14 (t, *J* = 7.5 Hz, 2H), 7.19 (td, *J* = 7.6, 1.2 Hz, 1H), 7.24–7.30 (m, 2H), 7.39–7.45 (m, 1H), 7.52 (td, *J* = 6.3, 1.4 Hz, 2H), 7.54–7.58 (m, 1H), 7.66 (t, *J* = 7.7 Hz, 1H), 7.72–7.76 (m, 2H), 7.77 (dd, *J* = 7.9, 1.6 Hz, 1H), 7.81 (dt, *J* = 7.9, 1.3 Hz, 1H), 7.91 (dt, *J* = 7.8, 1.4 Hz, 1H), 8.12 (t, *J* = 1.8 Hz, 1H), 8.61 (dd, *J* = 8.4, 1.2 Hz, 1H), 8.87 (s, 1H), 12.32 (s, 1H). ^13^C NMR (101 MHz, DMSO) δ 168.68, 164.73, 141.24, 139.72, 139.58, 139.07, 135.71, 132.73, 130.71, 130.14, 129.63, 129.57, 128.67, 128.47, 128.42, 127.29, 126.55, 126.26, 125.74, 123.37, 120.93, 120.62, 55.48, 40.58, 40.37, 40.17, 40.06, 39.96, 39.75, 39.54, 39.33, 37.19.

HRMS (ESI): calcd for C_29_H_24_N_2_O_4_ + Na: 487.1598 found 487.1629.

(2-([1,1′-biphenyl]-4-carboxamido)benzoyl)-*L*-phenylalanine (**16g**).

Synthesised according to general procedure 4 from compound **15g**. ^1^H NMR (400 MHz, DMSO) δ 3.10 (dd, *J* = 13.9, 11.0 Hz, 1H), 3.27 (dd, *J* = 13.8, 4.3 Hz, 1H), 4.76 (ddd, *J* = 11.7, 8.3, 4.2 Hz, 1H), 7.08–7.17 (m, 1H), 7.24 (dt, *J* = 8.6, 7.1 Hz, 3H), 7.34 (d, *J* = 7.1 Hz, 2H), 7.41–7.46 (m, 1H), 7.49–7.55 (m, 2H), 7.59 (ddd, *J* = 8.5, 7.4, 1.5 Hz, 1H), 7.76–7.83 (m, 3H), 7.89 (d, *J* = 8.6 Hz, 2H), 7.94 (d, *J* = 8.5 Hz, 2H), 8.66 (d, *J* = 8.3 Hz, 1H), 9.17 (d, *J* = 8.2 Hz, 1H), 12.19 (s, 1H), 12.96 (s, 1H). ^13^C NMR (101 MHz, DMSO) δ 173.16, 169.24, 164.49, 144.08, 139.74, 139.38, 138.42, 133.62, 133.00, 130.44, 129.57, 129.53, 128.84, 128.75, 128.66, 128.07, 127.65, 127.44, 127.29, 126.90, 123.30, 120.66, 120.29, 54.57, 40.60, 40.39, 40.18, 39.97, 39.77, 39.56, 39.35, 36.63. HRMS (ESI): calcd for C_29_H_24_N_2_O_4_ + Na: 487.1598 found 487.1629.

(2-([1,1′-biphenyl]-4-carboxamido)benzoyl)-*L*-tryptophan (**16h**).

Synthesised according to general procedure 4 from compound **15h**. ^1^H NMR (400 MHz, DMSO) δ 3.27 (dd, *J* = 14.7, 10.1 Hz, 1H), 4.75 (ddd, *J* = 10.1, 7.7, 4.4 Hz, 1H), 6.98 (ddd, *J* = 8.0, 6.9, 1.1 Hz, 1H), 7.05 (ddd, *J* = 8.1, 7.0, 1.3 Hz, 1H), 7.21 (td, *J* = 7.6, 1.2 Hz, 1H), 7.24 (d, *J* = 2.4 Hz, 1H), 7.31 (dt, *J* = 8.1, 1.0 Hz, 1H), 7.39–7.47 (m, 1H), 7.49–7.55 (m, 2H), 7.56–7.65 (m, 2H), 7.73–7.81 (m, 2H), 7.82–7.94 (m, 3H), 7.90–7.97 (m, 2H), 8.67 (dd, *J* = 8.4, 1.2 Hz, 1H), 9.11 (d, *J* = 7.8 Hz, 1H), 10.83 (d, *J* = 2.4 Hz, 1H), 12.30 (s, 1H). ^13^C NMR (101 MHz, DMSO) δ 169.03, 166.37, 164.51, 143.99, 143.24, 139.91, 139.61, 139.39, 136.58, 133.70, 133.47, 132.85, 129.55, 129.49, 128.90, 128.72, 128.52, 128.07, 127.81, 127.62, 127.41, 127.33, 126.90, 124.05, 124.01, 123.20, 121.35, 120.58, 120.44, 118.80, 118.76, 118.72, 111.88, 111.23, 54.65, 40.58, 40.37, 40.16, 39.95, 39.74, 39.53, 39.33, 31.15, 27.35. HRMS (ESI): calcd for C_29_H_24_N_2_O_4_ + H: 487.1598 found 487.1629.

*Tert*-butyl (*S*)-(3-(2-(2-(2-naphthamido)benzamido)-3-(1*H*-indol-3-yl)propanamido)propyl)carbamate (**17b**).

Synthesised according to general procedure 5 from compound **16b**. ^1^H NMR (400 MHz, DMSO) δ 1.33 (s, 9H), 1.51 (p, *J* = 6.8 Hz, 2H), 2.90 (q, *J* = 6.6 Hz, 2H), 3.10–3.23 (m, 2H), 3.23–3.34 (m, 2H), 4.73 (ddd, *J* = 10.1, 8.0, 4.5 Hz, 1H), 6.73 (t, *J* = 5.8 Hz, 1H), 6.95–7.00 (m, 1H), 7.00–7.06 (m, 1H), 7.20 (td, *J* = 7.6, 1.2 Hz, 1H), 7.24 (d, *J* = 2.3 Hz, 1H), 7.28 (d, *J* = 7.9 Hz, 1H), 7.55–7.63 (m, 1H), 7.63–7.67 (m, 1H), 7.69 (dd, *J* = 8.2, 6.6 Hz, 1H), 7.83 (dd, *J* = 8.0, 1.6 Hz, 1H), 7.88 (dd, *J* = 8.6, 1.8 Hz, 1H), 7.99–8.10 (m, 3H), 8.18 (t, *J* = 5.8 Hz, 1H), 8.46 (d, *J* = 1.8 Hz, 1H), 8.57–8.64 (m, 1H), 8.95 (d, *J* = 7.9 Hz, 1H), 10.79 (d, *J* = 2.5 Hz, 1H), 12.31 (s, 1H). ^13^C NMR (101 MHz, DMSO) δ 171.56, 169.17, 165.04, 164.25, 156.08, 139.62, 136.56, 134.87, 132.74, 132.66, 132.34, 129.55, 129.07, 128.57, 128.30, 128.18, 127.63, 127.52, 124.13, 123.68, 123.30, 121.39, 121.27, 120.88, 118.92, 118.69, 111.81, 110.97, 77.96, 54.99, 40.56, 40.35, 40.15, 39.94, 39.73, 39.52, 39.31, 38.42, 37.77, 36.79, 29.95, 28.66. HRMS (ESI): calcd for C_37_H_39_N_5_O_5_ + Na: 656.2898 found 656.2843.

*Tert*-butyl (*S*)-(3-(2-(2-(2-naphthamido)-5-bromobenzamido)-3-phenylpropanamido)propyl)carbamate (**17c**).

Synthesised according to general procedure 5 from compound **16c**.

^1^H NMR (400 MHz, DMSO) δ 1.33 (s, 9H), 1.51 (p, *J* = 6.8 Hz, 2H), 2.91 (q, *J* = 5.8 Hz, 2H), 2.94–3.04 (m, 1H), 3.09 (q, *J* = 6.7 Hz, 2H), 3.20 (dd, *J* = 13.8, 4.4 Hz, 1H), 4.74 (ddd, *J* = 10.9, 8.7, 4.2 Hz, 1H), 6.75 (s, 1H), 7.05 (t, *J* = 7.4 Hz, 1H), 7.20 (q, *J* = 7.5 Hz, 2H), 7.29–7.36 (m, 2H), 7.67 (pd, *J* = 6.9, 1.5 Hz, 2H), 7.73–7.82 (m, 1H), 7.85 (dd, *J* = 8.6, 1.8 Hz, 1H), 7.96 (d, *J* = 2.4 Hz, 1H), 8.00–8.05 (m, 1H), 8.09 (d, *J* = 8.3 Hz, 2H), 8.17 (t, *J* = 5.7 Hz, 1H), 8.41–8.47 (m, 1H), 8.48–8.59 (m, 1H), 9.21 (d, *J* = 8.4 Hz, 1H), 12.01 (s, 1H). HRMS (ESI): calcd for C_35_H_37_BrN_4_O_5_ + Na: 695.1798 found 695.1841.

*Tert*-butyl (*S*)-(3-(2-(2-(2-naphthamido)-5-bromobenzamido)-3-(1*H*-indol-3-yl)propanamido)propyl)carbamate (**17d**).

Synthesised according to general procedure 5 from compound **16d**.

^1^H NMR (400 MHz, DMSO) δ 1.33 (s, 9H), 1.51 (p, *J* = 6.8 Hz, 2H), 2.91 (q, *J* = 5.8 Hz, 2H), 2.94–3.04 (m, 1H), 3.09 (q, *J* = 6.7 Hz, 2H), 3.20 (dd, *J* = 13.8, 4.4 Hz, 1H), 4.74 (ddd, *J* = 10.9, 8.7, 4.2 Hz, 1H), 6.75 (s, 1H), 7.05 (t, *J* = 7.4 Hz, 1H), 7.20 (q, *J* = 7.5 Hz, 2H), 7.29–7.36 (m, 2H), 7.67 (pd, *J* = 6.9, 1.5 Hz, 2H), 7.73–7.82 (m, 1H), 7.85 (dd, *J* = 8.6, 1.8 Hz, 1H), 7.96 (d, *J* = 2.4 Hz, 1H), 8.00–8.05 (m, 1H), 8.09 (d, *J* = 8.3 Hz, 2H), 8.17 (t, *J* = 5.7 Hz, 1H), 8.41–8.47 (m, 1H), 8.48–8.59 (m, 1H), 9.21 (d, *J* = 8.4 Hz, 1H), 12.01 (s, 1H). ^13^C NMR (101 MHz, DMSO) δ 171.34, 167.82, 165.09, 162.80, 156.09, 138.91, 136.53, 135.30, 134.92, 132.64, 132.05, 131.59, 129.59, 129.12, 128.67, 128.40, 128.18, 127.64, 127.56, 124.05, 123.62, 123.01, 122.83, 121.39, 118.91, 118.72, 115.09, 111.81, 110.99, 77.96, 55.20, 40.59, 40.38, 40.17, 39.96, 39.75, 39.54, 39.33, 37.79, 36.79, 36.26, 29.95, 28.73, 28.67. HRMS (ESI): calcd for C_37_H_38_BrN_5_O_5_ + Na: 734.1998 found 734.1955.

*Tert*-butyl (*S*)-(3-(2-(2-([1,1′-biphenyl]-2-carboxamido)benzamido)-3-phenylpropanamido)propyl)carbamate (**17e**).

Synthesised according to general procedure 5 from compound **16e**. ^1^H NMR (400 MHz, DMSO) δ 1.36 (s, 9H), 1.40–1.47 (m, 2H), 2.86 (q, *J* = 6.6 Hz, 2H), 2.92–2.98 (m, 1H), 3.03 (dq, *J* = 12.9, 6.7 Hz, 2H), 3.12 (dd, *J* = 13.7, 4.3 Hz, 1H), 4.55 (s, 1H), 6.72 (s, 1H), 7.10–7.19 (m, 2H), 7.25 (t, *J* = 7.5 Hz, 3H), 7.28–7.38 (m, 6H), 7.45 (d, *J* = 7.2 Hz, 2H), 7.49 (dd, *J* = 7.3, 1.4 Hz, 1H), 7.51–7.55 (m, 1H), 7.58 (td, *J* = 7.4, 1.6 Hz, 1H), 7.61–7.67 (m, 1H), 8.04 (t, *J* = 5.8 Hz, 1H), 8.18 (d, *J* = 8.3 Hz, 1H), 8.78 (d, *J* = 8.2 Hz, 1H), 11.22 (s, 1H). ^13^C NMR (101 MHz, DMSO) δ 171.08, 168.49, 167.77, 156.07, 140.34, 140.05, 138.85, 138.74, 137.02, 132.32, 130.80, 130.76, 129.56, 128.78, 128.74, 128.71, 128.54, 127.95, 127.75, 126.76, 123.43, 122.05, 120.92, 77.97, 55.34, 40.59, 40.38, 40.17, 39.97, 39.76, 39.55, 39.34, 37.81, 37.54, 36.78, 29.85, 28.71. HRMS (ESI): calcd for C_37_H_40_N_4_O_5_ + H: 621.3078 found 621.3072.

*Tert*-butyl (*S*)-(3-(2-(2-([1,1′-biphenyl]-3-carboxamido)benzamido)-3-phenylpropanamido)propyl)carbamate (**17f**).

Synthesised according to general procedure 5 from compound **16f**. ^1^H NMR (400 MHz, DMSO) δ 1.34 (s, 9H), 1.49 (p, *J* = 7.2 Hz, 2H), 2.90 (q, *J* = 6.6 Hz, 2H), 2.98 (dd, *J* = 13.7, 10.8 Hz, 1H), 3.07 (ddt, *J* = 10.8, 6.9, 4.2 Hz, 2H), 3.19 (dd, *J* = 13.7, 4.2 Hz, 1H), 4.66–4.77 (m, 1H), 6.74 (t, *J* = 5.8 Hz, 1H), 7.07 (t, *J* = 7.4 Hz, 1H), 7.15–7.26 (m, 3H), 7.29–7.36 (m, 2H), 7.40–7.49 (m, 1H), 7.55 (dt, *J* = 13.1, 8.1 Hz, 3H), 7.66 (t, *J* = 7.7 Hz, 1H), 7.71–7.84 (m, 4H), 7.92 (dt, *J* = 7.9, 1.4 Hz, 1H), 8.09–8.17 (m, 2H), 8.54 (d, *J* = 8.3 Hz, 1H), 9.01 (d, *J* = 8.4 Hz, 1H), 12.07 (s, 1H). ^13^C NMR (101 MHz, DMSO) δ 171.05, 169.12, 164.89, 156.09, 141.24, 139.79, 139.33, 138.78, 135.74, 132.67, 130.72, 130.05, 129.62, 129.55, 129.03, 128.49, 128.45, 127.31, 126.70, 126.31, 125.81, 123.41, 121.59, 120.89, 77.97, 65.40, 55.43, 40.58, 40.37, 40.17, 39.96, 39.75, 39.54, 39.33, 37.82, 37.62, 36.82, 29.94, 28.68, 15.63. HRMS (ESI): calcd for C_37_H_40_N_4_O_5_ + H: 621.3078 found 621.3076.

*Tert*-butyl (*S*)-(3-(2-(2-([1,1′-biphenyl]-4-carboxamido)benzamido)-3-phenylpropanamido)propyl)carbamate (**17g**).

Synthesised according to general procedure 5 from compound **16g**.

^1^H NMR (400 MHz, DMSO) δ 1.32 (s, 9H), 1.53 (p, *J* = 6.8 Hz, 2H), 2.87–3.07 (m, 3H), 3.11 (q, *J* = 6.6 Hz, 2H), 3.20 (dd, *J* = 13.8, 4.1 Hz, 1H), 4.73 (ddd, *J* = 11.0, 8.3, 4.1 Hz, 1H), 6.76 (t, *J* = 5.8 Hz, 1H), 7.05–7.16 (m, 1H), 7.16–7.28 (m, 3H), 7.34 (td, *J* = 8.4, 1.4 Hz, 2H), 7.41–7.47 (m, 1H), 7.54 (dddd, *J* = 16.0, 7.8, 6.8, 1.6 Hz, 3H), 7.75–7.82 (m, 3H), 7.83–7.89 (m, 2H), 7.89–7.97 (m, 2H), 8.16 (t, *J* = 5.8 Hz, 1H), 8.59 (dt, *J* = 8.3, 1.8 Hz, 1H), 9.02 (d, *J* = 8.4 Hz, 1H), 12.12 (s, 1H). ^13^C NMR (101 MHz, DMSO) δ 171.05, 169.12, 164.89, 156.09, 141.24, 139.79, 139.33, 138.78, 135.74, 132.67, 130.72, 130.05, 129.62, 129.55, 129.03, 128.49, 128.45, 127.31, 126.70, 126.31, 125.81, 123.41, 121.59, 120.89, 77.97, 55.43, 40.58, 40.37, 40.17, 39.96, 39.75, 39.54, 39.33, 37.82, 37.62, 36.82, 29.94, 28.68, 15.63. HRMS (ESI): calcd for C_37_H_40_N_4_O_5_ + Na: 643.2898 found 643.2895.

*Tert*-butyl (*S*)-(3-(2-(2-([1,1′-biphenyl]-4-carboxamido)benzamido)-3-(1*H*-indol-3-yl)propanamido)propyl)carbamate (**17h**).

Synthesised according to general procedure 5 from compound **16h**. ^1^H NMR (400 MHz, DMSO) δ 1.32 (s, 9H), 1.52 (p, *J* = 6.8 Hz, 2H), 2.90–2.95 (m, 2H), 3.09 (d, *J* = 6.6 Hz, 1H), 3.12 (d, *J* = 6.6 Hz, 1H), 3.17 (dd, *J* = 14.6, 10.4 Hz, 1H), 3.28 (d, *J* = 4.3 Hz, 1H), 4.73 (s, 1H), 6.74 (t, *J* = 5.8 Hz, 1H), 6.94–7.08 (m, 2H), 7.19 (td, *J* = 7.6, 1.2 Hz, 1H), 7.23 (d, *J* = 2.4 Hz, 1H), 7.25–7.32 (m, 1H), 7.41–7.46 (m, 1H), 7.49–7.53 (m, 1H), 7.53–7.59 (m, 1H), 7.70 (d, *J* = 7.6 Hz, 1H), 7.73–7.79 (m, 2H), 7.79–7.87 (m, 3H), 7.88–7.94 (m, 2H), 8.17 (t, *J* = 5.8 Hz, 1H), 8.57–8.64 (m, 1H), 8.93 (d, *J* = 8.0 Hz, 1H), 10.78 (d, *J* = 2.4 Hz, 1H), 12.23 (s, 1H). ^13^C NMR (101 MHz, DMSO) δ 171.07, 169.17, 164.55, 156.09, 144.06, 139.59, 139.42, 138.83, 133.69, 132.73, 130.46, 129.78, 129.58, 129.55, 129.03, 128.73, 128.53, 128.10, 127.54, 127.46, 127.43, 126.74, 123.24, 121.08, 120.74, 77.94, 55.51, 40.61, 40.41, 40.20, 39.99, 39.78, 39.57, 39.36, 37.83, 37.60, 36.81, 32.58, 29.92, 28.66. HRMS (ESI): calcd for C_39_H_41_N_5_O_5_ + Na: 682.2998 found 682.2999.

(*S*)-3-(2-(2-([1,1′-biphenyl]-2-carboxamido)benzamido)-3-phenylpropanamido)propan-1-aminium (**2**).

Synthesised according to procedure 6 from compound **17e**.

^1^H NMR (400 MHz, DMSO) δ 1.61 (p, *J* = 7.0 Hz, 2H), 2.68 (d, *J* = 9.1 Hz, 2H), 2.95 (dd, *J* = 13.7, 10.3 Hz, 1H), 3.04–3.16 (m, 3H), 4.56 (ddd, *J* = 10.3, 8.0, 4.7 Hz, 1H), 7.13 (dd, *J* = 7.5, 1.2 Hz, 1H), 7.15–7.20 (m, 1H), 7.28 (dddd, *J* = 15.0, 7.8, 3.4, 1.8 Hz, 6H), 7.33–7.37 (m, 2H), 7.46 (tt, *J* = 7.6, 1.6 Hz, 2H), 7.50 (dd, *J* = 7.3, 1.3 Hz, 1H), 7.52–7.56 (m, 1H), 7.59 (td, *J* = 7.4, 1.7 Hz, 1H), 7.63 (dd, *J* = 7.8, 1.5 Hz, 1H), 7.68 (s, 2H), 8.17 (d, *J* = 8.4 Hz, 1H), 8.20 (d, *J* = 5.8 Hz, 1H), 8.81 (d, *J* = 8.1 Hz, 1H), 11.18 (s, 1H). ^13^C NMR (101 MHz, DMSO) δ 171.70, 168.49, 167.78, 140.28, 140.03, 138.78, 138.53, 136.99, 132.40, 130.83, 129.55, 128.82, 128.73, 128.60, 128.01, 127.80, 126.85, 123.50, 122.03, 121.01, 55.30, 40.58, 40.37, 40.16, 39.95, 39.74, 39.54, 39.33, 37.53, 37.11, 36.12, 27.72. HRMS (ESI): calcd for C_32_H_33_N_4_O_3_: 521.2578 found 521.2550.

(*S*)-3-(2-(2-([1,1′-biphenyl]-3-carboxamido)benzamido)-3-phenylpropanamido)propan-1-aminium (**3**).

Synthesised according to procedure 6 from compound **17f**.

^1^H NMR (400 MHz, DMSO) δ 1.63–1.71 (m, 2H), 2.72 (t, *J* = 7.3 Hz, 2H), 2.95–3.05 (m, 1H), 3.11–3.22 (m, 3H), 4.73 (s, 1H), 7.08 (t, *J* = 7.4 Hz, 1H), 7.18 (d, *J* = 7.7 Hz, 1H), 7.20–7.25 (m, 1H), 7.32 (d, *J* = 7.6 Hz, 2H), 7.45 (t, *J* = 7.4 Hz, 1H), 7.50–7.62 (m, 3H), 7.67 (t, *J* = 7.7 Hz, 1H), 7.71–7.77 (m, 2H), 7.80 (t, *J* = 8.0 Hz, 3H), 7.90–7.96 (m, 1H), 8.11 (d, *J* = 2.0 Hz, 1H), 8.34 (s, 1H), 8.48–8.55 (m, 1H), 9.04 (d, *J* = 8.4 Hz, 1H), 12.04 (s, 1H). ^13^C NMR (101 MHz, DMSO) δ 171.54, 169.04, 164.87, 141.22, 139.75, 139.34, 138.72, 135.73, 132.67, 130.72, 130.11, 129.64, 129.60, 129.15, 128.47, 127.31, 126.70, 126.31, 125.84, 123.43, 121.50, 120.89, 55.52, 40.58, 40.37, 40.17, 39.96, 39.75, 39.54, 39.33, 37.70, 37.02, 36.28, 27.62. HRMS (ESI): calcd for C_32_H_33_N_4_O_3_: 521.2578 found 521.2547.

(*S*)-3-(2-(2-([1,1′-biphenyl]-4-carboxamido)benzamido)-3-phenylpropanamido)propan-1-aminium (**4**).

Synthesised according to procedure 6 from compound **17h**.

^1^H NMR (400 MHz, DMSO) δ 1.69 (p, *J* = 7.0 Hz, 2H), 2.75 (p, *J* = 6.4 Hz, 2H), 2.97–3.05 (m, 1H), 3.18 (tt, *J* = 7.2, 2.9 Hz, 3H), 4.75 (ddd, *J* = 10.8, 8.3, 4.3 Hz, 1H), 7.09 (td, *J* = 7.2, 1.5 Hz, 1H), 7.22 (q, *J* = 7.9 Hz, 3H), 7.29–7.35 (m, 2H), 7.42–7.47 (m, 1H), 7.50–7.59 (m, 3H), 7.72–7.83 (m, 6H), 7.86 (d, *J* = 8.5 Hz, 2H), 7.90–7.94 (m, 2H), 8.35 (d, *J* = 7.6 Hz, 1H), 8.49–8.59 (m, 1H), 9.05 (d, *J* = 8.3 Hz, 1H), 12.06 (d, *J* = 16.0 Hz, 1H). ^13^C NMR (101 MHz, DMSO) δ 171.65, 169.13, 164.59, 144.08, 139.50, 139.38, 138.62, 133.69, 132.79, 129.64, 129.58, 129.05, 128.78, 128.56, 128.52, 128.13, 127.57, 127.46, 127.31, 126.81, 123.33, 121.11, 120.86, 55.42, 40.57, 40.36, 40.15, 39.95, 39.74, 39.53, 39.32, 37.66, 37.17, 36.24, 27.79. HRMS (ESI): calcd for C_32_H_33_N_4_O_3_: 521.2578 found 521.2546.

(*S*)-3-(2-(2-(2-naphthamido)benzamido)-3-(1*H*-indol-3-yl)propanamido)propan-1-aminium (**6**).

Synthesised according to procedure 6 from compound **17b**.

^1^H NMR (400 MHz, DMSO) δ 1.20–1.32 (m, 2H), 1.68 (p, *J* = 7.1 Hz, 2H), 2.74 (s, 3H), 3.04–3.16 (m, 2H), 3.19 (dd, *J* = 14.3, 8.5 Hz, 3H), 3.29 (dd, *J* = 14.6, 4.7 Hz, 1H), 3.53–3.60 (m, 2H), 4.75 (td, *J* = 9.0, 4.7 Hz, 1H), 6.94–7.08 (m, 2H), 7.18–7.26 (m, 2H), 7.29 (d, *J* = 7.9 Hz, 1H), 7.55–7.72 (m, 7H), 7.80–7.92 (m, 2H), 8.05 (td, *J* = 9.0, 6.1 Hz, 3H), 8.37 (t, *J* = 5.8 Hz, 1H), 8.46 (s, 1H), 8.57 (d, *J* = 8.3 Hz, 1H), 8.97 (d, *J* = 7.8 Hz, 1H), 10.78–10.83 (m, 1H), 12.25 (s, 1H). ^13^C NMR (101 MHz, DMSO) δ 172.27, 169.17, 165.10, 139.53, 136.57, 134.87, 132.81, 132.66, 132.35, 129.55, 129.11, 129.08, 128.64, 128.33, 128.19, 127.59, 124.17, 123.69, 123.41, 121.44, 121.35, 121.05, 118.86, 118.73, 111.87, 110.79, 66.36, 54.94, 54.03, 47.43, 42.29, 40.53, 40.32, 40.11, 39.90, 39.69, 39.48, 39.27, 38.42, 37.13, 36.17, 27.86, 27.80. HRMS (ESI): calcd for C_32_H_32_N_5_O_3_: 534.2578 found 534.2497.

(*S*)-3-(2-(2-([1,1′-biphenyl]-4-carboxamido)benzamido)-3-(1*H*-indol-3-yl)propanamido)propan-1-aminium (**7**).

Synthesised according to procedure 6 from compound **17h**.

^1^H NMR (400 MHz) δ 1.73 (p, *J* = 6.9 Hz, 2H), 2.79 (q, *J* = 6.6 Hz, 2H), 3.21 (q, *J* = 7.9 Hz, 3H), 3.32 (dd, *J* = 14.6, 4.4 Hz, 1H), 4.78 (ddd, *J* = 10.2, 7.9, 4.4 Hz, 1H), 7.00 (ddd, *J* = 8.0, 7.0, 1.2 Hz, 1H), 7.05 (ddd, *J* = 8.1, 7.0, 1.3 Hz, 1H), 7.20 (td, *J* = 7.6, 1.2 Hz, 1H), 7.26 (d, *J* = 2.4 Hz, 1H), 7.28–7.34 (m, 1H), 7.41–7.47 (m, 1H), 7.52 (t, *J* = 7.5 Hz, 2H), 7.58 (ddd, *J* = 8.6, 7.4, 1.5 Hz, 1H), 7.71 (d, *J* = 7.7 Hz, 1H), 7.73–7.80 (m, 2H), 7.81–7.89 (m, 5H), 7.90–7.96 (m, 2H), 8.41 (t, *J* = 5.9 Hz, 1H), 8.61 (dd, *J* = 8.4, 1.2 Hz, 1H), 9.00 (d, *J* = 8.0 Hz, 1H), 10.85 (d, *J* = 2.4 Hz, 1H), 12.25 (s, 1H). ^13^C NMR (101 MHz, DMSO) δ 172.94, 169.29, 165.68, 144.16, 139.01, 138.14, 136.44, 133.04, 132.79, 129.56, 128.79, 128.61, 128.08, 127.32, 127.16, 124.29, 124.23, 122.51, 121.80, 121.70, 121.42, 119.06, 118.61, 118.48, 115.54, 111.94, 110.15, 54.95, 39.42, 39.21, 39.11, 39.00, 38.79, 38.58, 38.37, 38.15, 37.03, 36.20, 27.76, 27.23. HRMS (ESI): calcd for C_34_H_34_N_5_O_3_: 560.2778 found 560.2658.

(*S*)-3-(2-(2-(2-naphthamido)-5-bromobenzamido)-3-phenylpropanamido)propan-1-aminium (**8**).

Synthesised according to procedure 6 from compound **17c**.

^1^H NMR (400 MHz, DMSO) δ 1.69 (p, *J* = 7.0 Hz, 2H), 2.75 (q, *J* = 6.6 Hz, 2H), 2.93–3.05 (m, 1H), 3.19 (ddd, *J* = 13.2, 10.0, 5.6 Hz, 3H), 4.76 (ddd, *J* = 10.7, 8.3, 4.6 Hz, 1H), 7.01–7.11 (m, 1H), 7.15–7.39 (m, 5H), 7.67 (dqd, *J* = 9.6, 6.9, 1.6 Hz, 2H), 7.74–7.81 (m, 4H), 7.81–7.90 (m, 1H), 7.98 (d, *J* = 2.4 Hz, 1H), 8.01–8.08 (m, 1H), 8.04–8.15 (m, 2H), 8.29 (s, 0H), 8.37 (t, *J* = 5.8 Hz, 1H), 8.44 (d, *J* = 1.8 Hz, 1H), 8.47–8.60 (m, 1H), 9.25 (d, *J* = 8.3 Hz, 1H), 11.99 (s, 1H). ^13^C NMR (101 MHz, DMSO) δ 171.47, 167.76, 165.10, 138.51, 138.47, 135.25, 134.95, 132.62, 131.96, 131.54, 129.54, 129.12, 128.73, 128.52, 128.42, 128.22, 127.65, 126.83, 123.68, 123.51, 123.09, 115.23, 55.38, 40.52, 40.31, 40.10, 39.89, 39.69, 39.48, 39.27, 37.63, 37.18, 36.27, 27.79. HRMS (ESI): calcd for C_30_H_30_BrN_4_O_3_: 573.1578 found 573.1499.

(*S*)-3-(2-(2-(2-naphthamido)-5-bromobenzamido)-3-(1*H*-indol-3-yl)propanamido)propan-1-aminium (**9**).

Synthesised according to procedure 6 from compound **17d**.

^1^H NMR (400 MHz, DMSO) δ 1.67 (p, *J* = 6.8 Hz, 2H), 2.74 (s, 2H), 3.17 (q, *J* = 8.1 Hz, 3H), 3.29 (dd, *J* = 14.6, 4.6 Hz, 1H), 4.69–4.79 (m, 1H), 6.94–7.07 (m, 2H), 7.22 (d, *J* = 2.4 Hz, 1H), 7.24–7.30 (m, 1H), 7.67 (tdd, *J* = 11.4, 5.2, 2.9 Hz, 7H), 7.79 (dd, *J* = 8.9, 2.3 Hz, 1H), 7.85 (dd, *J* = 8.7, 1.9 Hz, 1H), 7.98–8.10 (m, 4H), 8.36 (t, *J* = 5.9 Hz, 1H), 8.45 (d, *J* = 1.8 Hz, 1H), 8.54 (d, *J* = 8.9 Hz, 1H), 9.19 (d, *J* = 7.9 Hz, 1H), 10.82 (d, *J* = 2.4 Hz, 1H), 12.18 (s, 1H). ^13^C NMR (101 MHz, DMSO) δ 172.01, 167.83, 165.12, 138.84, 136.55, 135.35, 134.93, 132.64, 132.06, 131.62, 129.59, 129.13, 128.71, 128.44, 128.20, 127.61, 124.09, 123.62, 123.09, 122.97, 121.43, 118.85, 118.74, 115.17, 111.86, 110.81, 55.14, 40.60, 40.40, 40.19, 39.98, 39.77, 39.56, 39.35, 37.15, 36.20, 27.82. HRMS (ESI): calcd for C_32_H_31_BrN_5_O_3_^+^: 612.1605 found 612.1611.

*tert*-butyl (*S*)-(3-(2-((((9*H*-fluoren-9-yl)methoxy)carbonyl)amino)-3-phenylpropanamido)propyl)carbamate (**20**).

Fmoc-phenylalanine **18** (1 equiv), N-Boc-1,3-diaminopropane **19** (1.1 equiv), and HATU (1.2 equiv) were dissolved in anhydrous DMF and cooled to 0 °C. DIPEA (2 equiv) was then added dropwise, and the reaction was stirred at 0 °C for 1 h. Following completion of the reaction as observed via TLC, the reaction mixture was concentrated under reduced pressure and the residue extracted with ethyl acetate. The organic layer was washed with water (1×), 1M HCl (1×), sodium bicarbonate (3×), and brine (1×). The crude extract was then dried over magnesium sulphate, and concentrated under reduced pressure, providing white solid **20** with a 54–70% yield. ^1^H NMR (400 MHz, DMSO) δ 1.38 (s, 9H), 1.48 (p, *J* = 6.9 Hz, 2H), 2.80 (dd, *J* = 13.6, 10.2 Hz, 1H), 2.90 (q, *J* = 6.6 Hz, 2H), 2.97 (dd, *J* = 13.5, 4.5 Hz, 1H), 3.07 (dq, *J* = 13.9, 6.7 Hz, 2H), 4.11–4.22 (m, 4H), 6.76 (t, *J* = 5.8 Hz, 1H), 7.15–7.22 (m, 1H), 7.23–7.34 (m, 6H), 7.41 (td, *J* = 7.5, 3.0 Hz, 2H), 7.59–7.68 (m, 3H), 7.88 (d, *J* = 7.6 Hz, 2H), 7.97 (t, *J* = 5.7 Hz, 1H). ^13^C NMR (101 MHz, DMSO) δ 171.76, 156.23, 156.06, 144.22, 141.13, 138.65, 129.67, 128.51, 128.09, 127.52, 126.70, 125.83, 125.76, 120.55, 77.94, 66.09, 56.77, 47.03, 40.61, 40.40, 40.19, 39.99, 39.78, 39.57, 39.36, 37.92, 36.74, 29.92, 28.72. HRMS (ESI): calcd for C_32_H_37_N_3_O_5_ + Na: 566.2598 found 566.2626.

*tert*-butyl (*S*)-(3-(2-amino-3-phenylpropanamido)propyl)carbamate (**21**).

Fmoc-phenylalanine linked to the diamino propyl group **15** was dissolved in DMF and piperidine was added. The reaction mixture was left to stir at room temperature overnight. Following reaction completion observed via TLC and ninhydrin staining, the crude mixture was extracted with chloroform and washed repeatedly with water until no DMF remained. The crude reaction mixture was then concentrated and purified by column chromatography using chloroform and 10% ammonia in methanol, giving a white solid **21** with a 33–52% yield. ^1^H NMR (400 MHz, DMSO) δ 1.38 (s, 9H), 1.46 (q, *J* = 6.9 Hz, 3H), 2.63 (dd, *J* = 13.3, 8.0 Hz, 1H), 2.88 (dt, *J* = 13.0, 5.9 Hz, 3H), 3.02–3.10 (m, 2H), 3.37 (dd, *J* = 8.0, 5.2 Hz, 1H), 6.75 (q, *J* = 5.5 Hz, 1H), 7.17–7.22 (m, 3H), 7.26 (q, *J* = 6.8 Hz, 2H), 7.83 (d, *J* = 6.1 Hz, 1H), 8.31 (s, 1H). ^13^C NMR (101 MHz, DMSO) δ 174.76, 156.05, 139.25, 129.74, 129.54, 128.54, 126.53, 77.92, 56.83, 41.70, 40.61, 40.40, 40.19, 39.98, 39.77, 39.56, 39.35, 37.86, 36.42, 30.01, 28.72. HRMS (ESI): calcd for C_17_H_27_N_3_O_3_ + H: 322.2178 found 322.2125.

*tert*-butyl (*S*)-(3-(2-(2-aminobenzamido)-3-phenylpropanamido)propyl)carbamate (**22a**).

Synthesised according to procedure 1 from compound **21**.

^1^H NMR (400 MHz, DMSO) δ 1.37 (s, 9H), 1.49 (p, *J* = 6.7 Hz, 2H), 2.91 (q, *J* = 6.6 Hz, 2H), 2.98 (dd, *J* = 13.7, 10.2 Hz, 1H), 3.02–3.11 (m, 3H), 4.58 (ddd, *J* = 10.0, 8.2, 4.7 Hz, 1H), 6.27 (s, 2H), 6.49 (ddd, *J* = 8.1, 7.1, 1.2 Hz, 1H), 6.64 (dd, *J* = 8.2, 1.2 Hz, 1H), 6.76 (t, *J* = 5.8 Hz, 1H), 7.11 (ddd, *J* = 8.4, 7.1, 1.5 Hz, 1H), 7.15–7.19 (m, 1H), 7.23–7.28 (m, 2H), 7.29–7.34 (m, 2H), 7.48 (dd, *J* = 8.0, 1.6 Hz, 1H), 8.01 (t, *J* = 5.7 Hz, 1H), 8.20 (d, *J* = 8.2 Hz, 1H). ^13^C NMR (101 MHz, DMSO) δ 171.92, 169.17, 150.07, 139.00, 132.25, 129.59, 128.92, 128.52, 126.66, 116.67, 114.88, 114.68, 77.97, 55.10, 40.60, 40.39, 40.18, 39.97, 39.77, 39.56, 39.35, 36.77, 28.72. HRMS (ESI): calcd for C_24_H_32_N_4_O_4_ + H: 441.2478 found 441.2495.

*tert*-butyl (*S*)-(3-(2-(2-amino-5-bromobenzamido)-3-phenylpropanamido)propyl)carbamate (**22b**).

Synthesised according to procedure 1 from compound **21**.

^1^H NMR (400 MHz, DMSO) δ 1.37 (s, 9H), 1.49 (p, *J* = 7.0 Hz, 2H), 2.86–3.01 (m, 3H), 3.01–3.12 (m, 3H), 4.58 (ddd, *J* = 10.4, 8.2, 4.6 Hz, 1H), 6.44 (s, 2H), 6.63 (d, *J* = 8.8 Hz, 1H), 6.76 (t, *J* = 5.9 Hz, 1H), 7.13–7.22 (m, 1H), 7.21–7.27 (m, 1H), 7.23–7.30 (m, 2H), 7.27–7.35 (m, 2H), 7.70 (d, *J* = 2.4 Hz, 1H), 8.03 (t, *J* = 5.8 Hz, 1H), 8.45 (d, *J* = 8.2 Hz, 1H). ^13^C NMR (101 MHz, DMSO) δ 171.75, 167.95, 156.08, 149.29, 138.96, 134.67, 131.04, 129.53, 128.53, 126.69, 118.75, 116.13, 105.30, 77.97, 55.15, 40.59, 40.38, 40.17, 39.96, 39.75, 39.54, 39.34, 37.89, 37.62, 36.77, 29.96, 28.72. HRMS (ESI): calcd for C_24_H_31_BrN_4_O_4_ + H: 519.1578 found 519.1604.

(*S*)-2-((1-((3-ammoniopropyl)amino)-1-oxo-3-phenylpropan-2-yl)carbamoyl)benzenaminium (**5**).

Synthesised according to procedure 6 from compound **22a**.

^1^H NMR (400 MHz, DMSO) δ 1.69 (p, *J* = 7.1 Hz, 2H), 2.77 (q, *J* = 6.6 Hz, 2H), 3.00 (dd, *J* = 13.7, 9.9 Hz, 1H), 3.07 (dd, *J* = 13.7, 5.0 Hz, 1H), 3.16 (q, *J* = 6.5 Hz, 2H), 4.57 (ddd, *J* = 10.0, 8.1, 5.0 Hz, 1H), 6.47–6.53 (m, 1H), 6.66 (dd, *J* = 8.3, 1.2 Hz, 1H), 7.13 (ddd, *J* = 8.4, 7.0, 1.5 Hz, 1H), 7.15–7.21 (m, 1H), 7.27 (t, *J* = 7.6 Hz, 2H), 7.30–7.36 (m, 2H), 7.50 (dd, *J* = 8.0, 1.5 Hz, 1H), 7.77 (s, 3H), 8.20 (t, *J* = 5.9 Hz, 1H), 8.27 (d, *J* = 8.0 Hz, 1H). ^13^C NMR (101 MHz, DMSO) δ 172.50, 169.29, 150.10, 138.89, 132.34, 129.57, 128.96, 128.58, 126.74, 116.73, 114.92, 114.57, 55.26, 40.60, 40.39, 40.18, 39.97, 39.76, 39.55, 39.34, 37.62, 37.20, 36.12, 27.83. HRMS (ESI): calcd for C_19_H_24_N_4_O_2_ + H: 341.1971 found 341.1971.

(*S*)-2-((1-((3-ammoniopropyl)amino)-1-oxo-3-phenylpropan-2-yl)carbamoyl)-4-bromobenzenaminium (**10**).

Synthesised according to procedure 6 from compound **22b**.

^1^H NMR (400 MHz, DMSO) δ 1.68 (p, *J* = 7.0 Hz, 2H), 2.76 (q, *J* = 6.5 Hz, 2H), 2.94–3.11 (m, 2H), 3.15 (q, *J* = 6.5 Hz, 2H), 4.57 (dq, *J* = 9.2, 5.2 Hz, 1H), 6.45 (s, 2H), 6.64 (d, *J* = 8.8 Hz, 1H), 7.19 (t, *J* = 7.1 Hz, 1H), 7.22–7.35 (m, 5H), 7.71 (d, *J* = 2.4 Hz, 1H), 7.76 (s, 4H), 8.22 (t, *J* = 5.8 Hz, 1H), 8.50 (d, *J* = 8.0 Hz, 1H). ^13^C NMR (101 MHz, DMSO) δ 172.31, 168.06, 149.33, 138.84, 134.75, 131.07, 129.52, 128.58, 126.77, 118.79, 116.01, 105.32, 55.27, 40.59, 40.38, 40.17, 39.96, 39.75, 39.54, 39.34, 37.50, 37.19, 36.14, 27.82. HRMS (ESI): calcd for C_19_H_23_BrN_4_O_2_ + H: 419.1178 found 419.1076.

di-*tert*-butyl ((*S*)-6-((3-((*S*)-2-(2-([1,1′-biphenyl]-3-carboxamido)benzamido)-3-phenylpropanamido)propyl)amino)-6-oxohexane-1,5-diyl)dicarbamate (**23**).

Commercially available *N*_α_,*N*_ε_-Di-Boc-L-lysine hydroxysuccinimide ester was dissolved in DMF, followed by the addition of peptide-mimic **3**. Finally, DIPEA was added to the reaction mixture, and the reaction was allowed to stir overnight. Following reaction completion observed via TLC, the crude mixture was extracted with ethyl acetate and washed repeatedly with water until no DMF remained. This was followed by a wash with 0.1 M HCl solution, and then with brine. The crude reaction mixture was then concentrated and purified by column chromatography using hexane/ethyl acetate, giving white solid **23** with a 74% yield. ^1^H NMR (400 MHz, DMSO) δ 1.34–1.39 (m, 21H), 1.49 (q, *J* = 7.7 Hz, 4H), 2.74 (s, 1H), 2.90 (s, 4H), 2.93–3.14 (m, 3H), 3.17 (dd, *J* = 13.7, 4.3 Hz, 1H), 3.74–3.84 (m, 1H), 4.72 (dddd, *J* = 10.4, 8.3, 4.2, 1.7 Hz, 1H), 6.75 (dd, *J* = 8.7, 4.9 Hz, 2H), 7.03–7.12 (m, 1H), 7.15–7.26 (m, 3H), 7.29–7.35 (m, 2H), 7.40–7.49 (m, 1H), 7.49–7.61 (m, 3H), 7.66 (t, *J* = 7.7 Hz, 1H), 7.74 (dt, *J* = 6.1, 1.3 Hz, 3H), 7.79 (ddt, *J* = 10.0, 8.1, 1.5 Hz, 2H), 7.89–7.98 (m, 1H), 8.09–8.18 (m, 2H), 8.54 (dd, *J* = 8.3, 1.2 Hz, 1H), 9.01 (d, *J* = 8.4 Hz, 1H), 12.05–12.10 (m, 1H). ^13^C NMR (101 MHz, DMSO) δ 172.63, 171.10, 169.10, 164.85, 162.78, 156.05, 141.24, 139.78, 139.34, 138.75, 135.74, 132.67, 130.73, 130.05, 129.61, 129.55, 129.06, 128.49, 128.45, 127.31, 126.70, 126.29, 125.82, 123.39, 120.86, 78.40, 77.80, 55.43, 40.62, 40.41, 40.20, 39.99, 39.78, 39.57, 39.37, 37.67, 36.49, 36.25, 31.24, 29.67, 28.74, 28.68, 28.63, 25.70, 23.33. HRMS (ESI): calcd for C_48_H_60_N_6_O_8_ + H: 849.4578 found 849.4555.

(*S*)-6-((3-((*S*)-2-(2-([1,1′-biphenyl]-3-carboxamido)benzamido)-3-phenylpropanamido)propyl)amino)-6-oxohexane-1,5-diaminium (**11**).

Synthesised according to procedure 6 from compound **23**.

^1^H NMR (400 MHz, DMSO) δ 1.32 (p, *J* = 7.9 Hz, 2H), 1.55 (dt, *J* = 16.1, 7.5 Hz, 4H), 1.68 (dt, *J* = 14.6, 5.2 Hz, 3H), 2.77 (dq, *J* = 11.7, 6.0 Hz, 2H), 2.99 (dd, *J* = 13.8, 10.8 Hz, 1H), 3.03–3.22 (m, 4H), 4.73 (ddd, *J* = 10.8, 8.3, 4.2 Hz, 1H), 7.08 (t, *J* = 7.3 Hz, 1H), 7.16–7.23 (m, 2H), 7.23–7.30 (m, 2H), 7.32 (d, *J* = 7.6 Hz, 2H), 7.45 (t, *J* = 7.3 Hz, 1H), 7.53 (d, *J* = 7.5 Hz, 1H), 7.56 (dd, *J* = 5.5, 3.2 Hz, 1H), 7.66 (t, *J* = 7.7 Hz, 1H), 7.75 (d, *J* = 7.1 Hz, 2H), 7.81 (dd, *J* = 15.0, 7.4 Hz, 4H), 7.90–7.96 (m, 1H), 8.12 (t, *J* = 1.9 Hz, 1H), 8.17 (s, 3H), 8.20–8.26 (m, 1H), 8.45 (t, *J* = 5.6 Hz, 1H), 8.50 (d, *J* = 8.3 Hz, 1H), 9.01 (d, *J* = 8.4 Hz, 1H), 12.03 (d, *J* = 3.1 Hz, 1H). ^13^C NMR (101 MHz, DMSO) δ 171.42, 171.20, 171.18, 169.05, 168.75, 164.91, 158.86, 158.55, 141.24, 139.77, 139.21, 138.69, 135.73, 132.67, 130.75, 130.05, 129.63, 129.55, 129.03, 128.49, 128.22, 127.32, 126.74, 126.33, 125.84, 123.50, 121.84, 121.81, 121.18, 121.05, 119.08, 55.39, 52.55, 52.22, 40.61, 40.40, 40.19, 39.98, 39.77, 39.56, 39.35, 38.92, 37.73, 37.01, 30.97, 29.37, 29.25, 27.02, 25.69, 21.85, 21.71. HRMS (ESI): calcd for C_38_H_46_N_6_O_4_: 650.3678 found 650.3528.

(*S*)-*N*-(2-((1-((3-di-*tert*-butyl-guanidinopropyl)amino)-1-oxo-3-phenylpropan-2-yl)carbamoyl)phenyl)-[1,1′-biphenyl]-3-carboxamide (**24**).

Peptide-mimic **3** was suspended in ACN, followed by the addition of DIPEA (2.5 equiv). *N*,*N*’-Di-Boc-1H-pyrazole-1-carboxamidine (1.2 equiv) was then added, and the reaction was left to stir overnight. The next morning, the reaction was diluted in EtOAc and washed with water (1×) and brine (1×). The resulting organic phase was dried over magnesium sulphate and concentrated under reduced pressure. The resulting crude mixture was purified using flash chromatography with hexane/ethyl acetate to produce **24** as a white solid with a 67% yield. ^1^H NMR (400 MHz, DMSO) δ 1.37 (s, 8H), 1.45 (s, 9H), 1.48 (s, 1H), 1.56 (tt, *J* = 13.5, 6.5 Hz, 2H), 2.96–3.25 (m, 6H), 4.68 (ddd, *J* = 10.6, 8.3, 4.6 Hz, 1H), 7.07–7.12 (m, 1H), 7.18–7.24 (m, 3H), 7.31–7.35 (m, 2H), 7.41–7.46 (m, 1H), 7.50–7.56 (m, 2H), 7.56–7.60 (m, 1H), 7.64 (t, *J* = 7.7 Hz, 1H), 7.72 (q, *J* = 1.7 Hz, 1H), 7.73–7.78 (m, 2H), 7.81 (dt, *J* = 7.9, 1.3 Hz, 1H), 7.89 (ddd, *J* = 7.7, 1.9, 1.1 Hz, 1H), 8.11 (t, *J* = 1.8 Hz, 1H), 8.21 (t, *J* = 5.8 Hz, 1H), 8.31 (t, *J* = 5.7 Hz, 1H), 8.54 (dd, *J* = 8.4, 1.2 Hz, 1H), 9.02 (d, *J* = 8.2 Hz, 1H), 11.46 (s, 1H), 12.08 (s, 1H). ^13^C NMR (101 MHz, DMSO) δ 171.21, 170.83, 169.17, 164.89, 163.59, 155.75, 152.39, 141.24, 139.81, 139.37, 138.72, 135.74, 132.68, 130.69, 129.96, 129.58, 129.05, 128.51, 128.40, 128.21, 127.30, 126.73, 126.27, 125.78, 123.39, 121.56, 121.18, 120.90, 104.67, 83.24, 78.57, 60.23, 40.58, 40.38, 40.17, 39.96, 39.75, 39.54, 39.33, 38.07, 37.60, 36.33, 31.75, 31.15, 30.83, 29.25, 28.72, 28.43, 28.27, 28.05, 21.22, 14.54, 0.64. HRMS (ESI): calcd for C_38_H_46_N_6_O_4_: 763.3778 found 763.3814.

(*S*)-1-(3-(2-(2-([1,1′-biphenyl]-3-carboxamido)benzamido)-3-phenylpropanamido)propyl)guanidinium (**12**).

Synthesised according to procedure 6 from compound **24**. ^1^H NMR (400 MHz, DMSO) δ 1.37 (s, 8H), 1.45 (s, 9H), 1.48 (s, 1H), 1.56 (tt, *J* = 13.5, 6.5 Hz, 2H), 2.96–3.25 (m, 6H), 4.68 (ddd, *J* = 10.6, 8.3, 4.6 Hz, 1H), 7.07–7.12 (m, 1H), 7.18–7.24 (m, 3H), 7.31–7.35 (m, 2H), 7.41–7.46 (m, 1H), 7.50–7.56 (m, 2H), 7.56–7.60 (m, 1H), 7.64 (t, *J* = 7.7 Hz, 1H), 7.72 (q, *J* = 1.7 Hz, 1H), 7.73–7.78 (m, 2H), 7.81 (dt, *J* = 7.9, 1.3 Hz, 1H), 7.89 (ddd, *J* = 7.7, 1.9, 1.1 Hz, 1H), 8.11 (t, *J* = 1.8 Hz, 1H), 8.21 (t, *J* = 5.8 Hz, 1H), 8.31 (t, *J* = 5.7 Hz, 1H), 8.54 (dd, *J* = 8.4, 1.2 Hz, 1H), 9.02 (d, *J* = 8.2 Hz, 1H), 11.46 (s, 1H), 12.08 (s, 1H). ^13^C NMR (101 MHz, DMSO) δ 171.37, 169.11, 164.93, 157.19, 141.25, 139.78, 139.23, 138.65, 135.73, 132.70, 130.75, 130.04, 129.63, 129.56, 129.04, 128.51, 128.47, 127.32, 126.76, 126.31, 125.83, 123.50, 121.76, 121.04, 55.41, 40.59, 40.38, 40.17, 39.96, 39.75, 39.54, 39.33, 38.76, 37.65, 36.48, 29.14. HRMS (ESI): calcd for C_38_H_46_N_6_O_4_: 563.2878 found 563.2767.

## 4. Conclusions

Within the presented work, a library of peptide-mimicking LMWGs containing varying amino acids and a range of structurally differing aromatic caps has been synthesised in high yields. Of the synthesised structures, three novel hydrogels at low concentration have been synthesised under physiological pH, and many features that hinder gelation have been identified. Furthermore, a method for tuning the physical features of the hydrogels has been identified by altering the position of secondary phenyl groups within the aromatic cap. This finding could be largely beneficial as a simple guide to improving the physical properties of similar gels in the future. These peptide-mimics have been synthesised on a gram-scale with commercially available and economical starting materials, making this synthetic method preferable over current solid-phase peptide-based LMWGs. Hydrogel **7** showed the best activity against both *S. aureus* and *E. coli*. The structural features of these hydrogels provide a general guide to lead the rational design of future hydrogels that will improve desired physical hydrogel characteristics. Furthermore, utilising the ability of these compounds to form supramolecular fibres resulting in hydrogels highlights the potential for more active antibacterial gels that could reduce the risk of infection as wound dressings.

## Figures and Tables

**Table 1:** List of synthesised peptide-mimics and their gelled forms. OG = opaque gel, P = precipitate, N = no gelation. n/a is used whereby CGC is not applicable.

Compound	Amino Acid	Capping Group	X	Cationic Group	Gel Formation	CGC (*w*/*v*)
**1**	Phe	Naphthoyl	H	NH_3_^+^	OG	0.1 [30]
**2**	Phe	2-B.P.	H	NH_3_^+^	N	n/a
**3**	Phe	3-B.P.	H	NH_3_^+^	OG	0.5%
**4**	Phe	4-B.P.	H	NH_3_^+^	OG	0.5%
**5**	Phe	NH_3_^+^	H	NH_3_^+^	N	n/a
**6**	Trp	Naphthoyl	H	NH_3_^+^	N	n/a
**7**	Trp	4-B.P.	H	NH_3_^+^	OG + P	0.5%
**8**	Phe	NH_3_^+^	Br	NH_3_^+^	N	n/a
**9**	Phe	Naphthoyl	Br	NH_3_^+^	N	n/a
**10**	Trp	Naphthoyl	Br	NH_3_^+^	N	n/a
**11**	Phe	3-B.P.	H	Lys^2+^	N	n/a
**12**	Phe	3-B.P.	H	Guan^+^	N	n/a

**Table 2:** Diameters of xerogel fibres recorded following AFM imaging.

Hydrogel	Ø (nm)
Cpd **1** Phe, Naphthoyl	115 (±5) [30]
Cpd **3** Phe, 3′-Biphenyl	26 (±3)
Cpd **4** Phe, 4′-Biphenyl	48 (±6)
Cpd **7** Trp, 4′-Biphenyl	38 (±5)

## Data Availability

Data is contained within the article and Supplementary Materials.

## Supplementary Materials

The following supporting information can be downloaded at: https://www.mdpi.com/article/10.3390/antibiotics14111118/s1, File S1: ^1^H NMR and ^13^C NMR data.

## Abbreviations

The following abbreviations are used in this manuscript:

4-DMAP	4-Dimethylaminopyridine
AFM	Atomic force microscopy
BP	Biphenyl
CD	Circular dichroism
DCM	Dichloromethane
DIPEA	Diisopropylethylamine
DMF	Dimethylformamide
*E. coli*	*Escherichia coli*
Equiv	Equivalents
Fmoc	Fluorenylmethoxycarbonyl
FST	Frequency sweep test
HOBt	Hydroxybenzotriazole
LMWG	Low-molecular-weight gelator
LVER	Linear viscoelastic region
MeOH	Methanoil
MHB	Mueller Hinton Broth
MQ	Milli-Q
Napth	Naphthoyl
OD	Optical density
Phe	Phenylalanine
*S. aureus*	*Staphylococcus aureus*
SFR	Structure–functional relationship
SST	Strain sweep test
TEA	Triethylamine
TFA	Trifluoroacetic acid
THF	Tetrahydrofuran
Trp	Tryptophan
