# Supplementary material for: Tuning the Structure–Functional Properties Within Peptide-Mimicking Antimicrobial Hydrogels
Source: Antibiotics (Basel). 2025 Nov 5;14(11):1118. doi: 10.3390/antibiotics14111118 (PMC12649725; doi:10.3390/antibiotics14111118)

# The Supplementary Data for

# Tuning the Structure-Functional Properties within Peptide-Mimicking Antimicrobial Hydrogels

**Samuel T. Attard, Vina R. Aldilla, Rajesh Kuppusamy, Renxun Chen, David StC Black, Pall Thoradson, Mark D. P. Willcox, and Naresh Kumar**

The analytical data for intermediates **14a-d**, **15a-d**, **16a-d** and **17a** and final compound **1** were already mentioned in previous publications [27,46].

$^{1}$H NMR of methyl (2-([1,1'-biphenyl]-2-carboxamido)benzoyl)-*L*-phenylalaninate (15e)

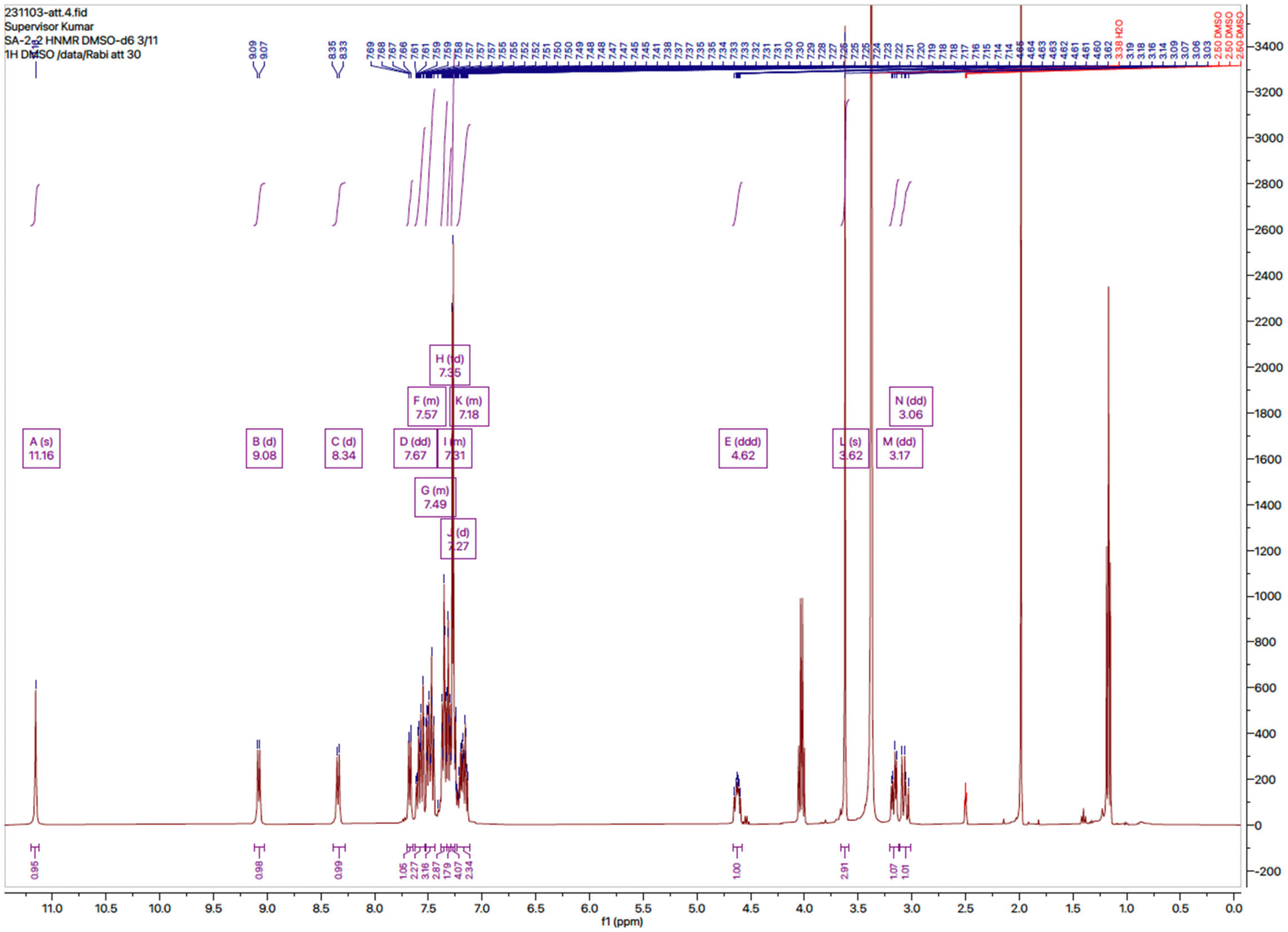

$^{13}C$ NMR of methyl (2-([1,1'-biphenyl]-2-carboxamido)benzoyl)-*L*-phenylalaninate (15e)

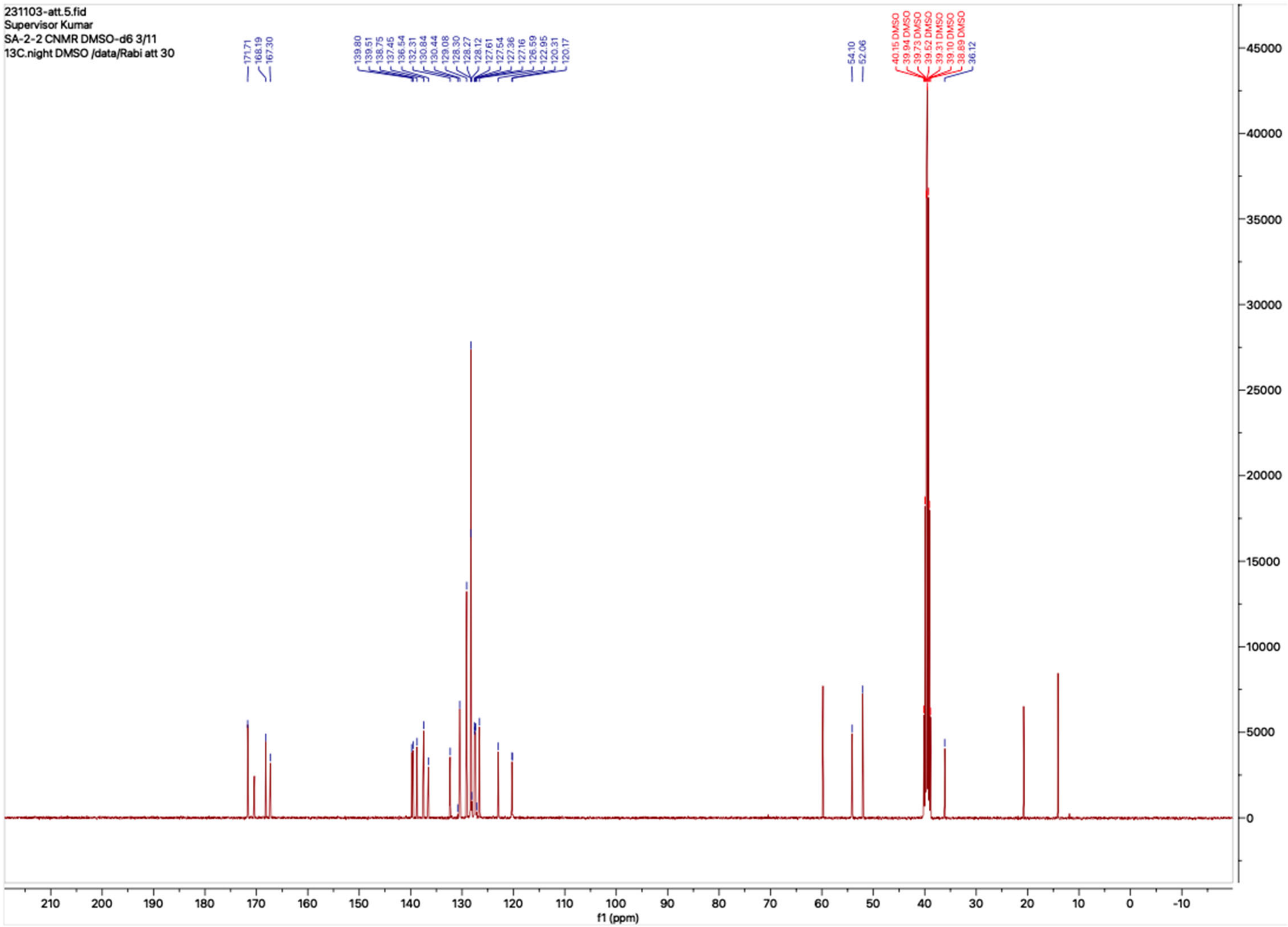

$^{1}$H NMR of Methyl (2-([1,1'-biphenyl]-3-carboxamido)benzoyl)-*L*-phenylalaninate (15f)

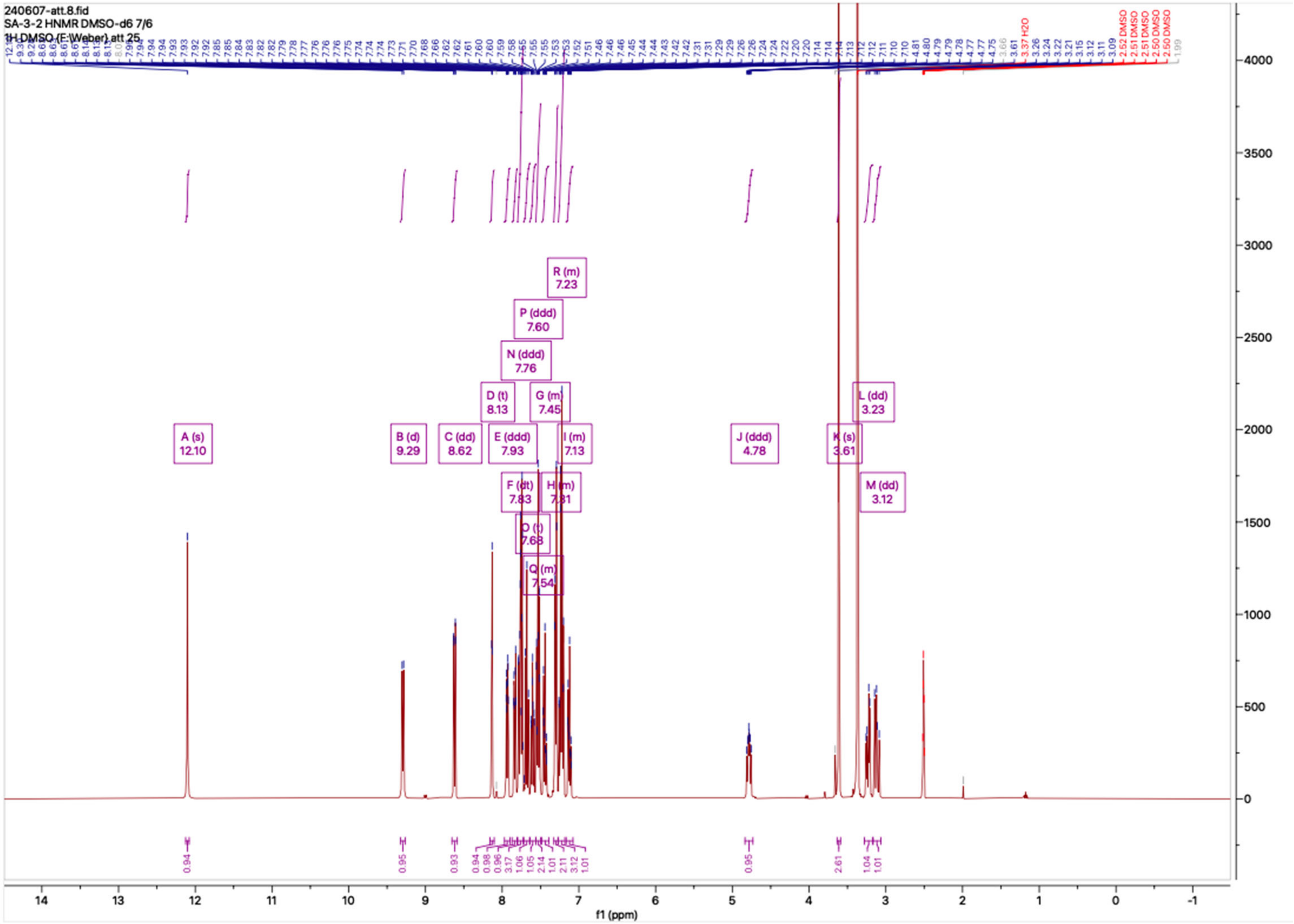

$^{13}$C NMR of Methyl (2-([1,1'-biphenyl]-3-carboxamido)benzoyl)-*L*-phenylalaninate (15f)

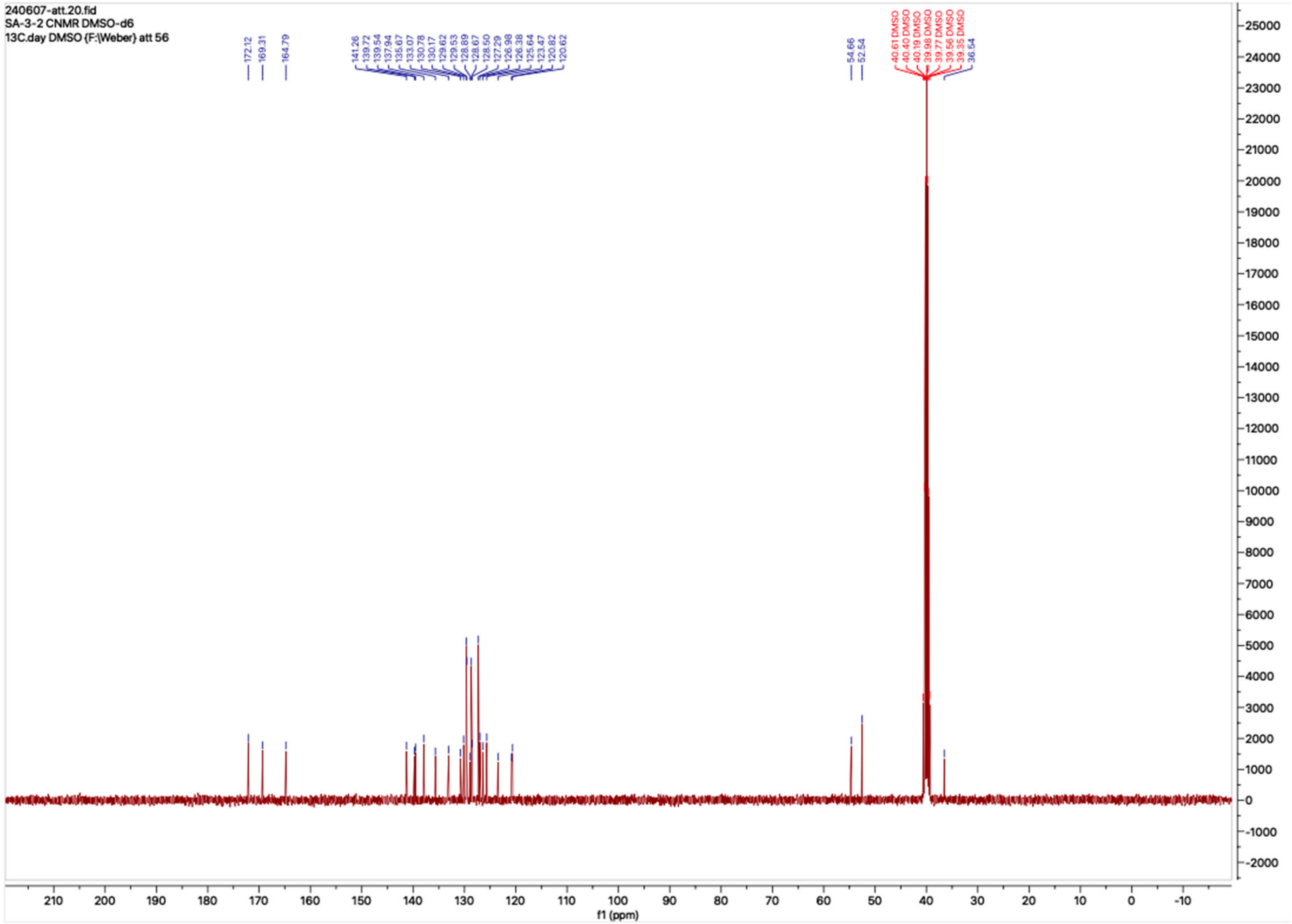

$^{1}$H NMR of Methyl (2-([1,1'-biphenyl]-4-carboxamido)benzoyl)-*L*-phenylalaninate (15g)

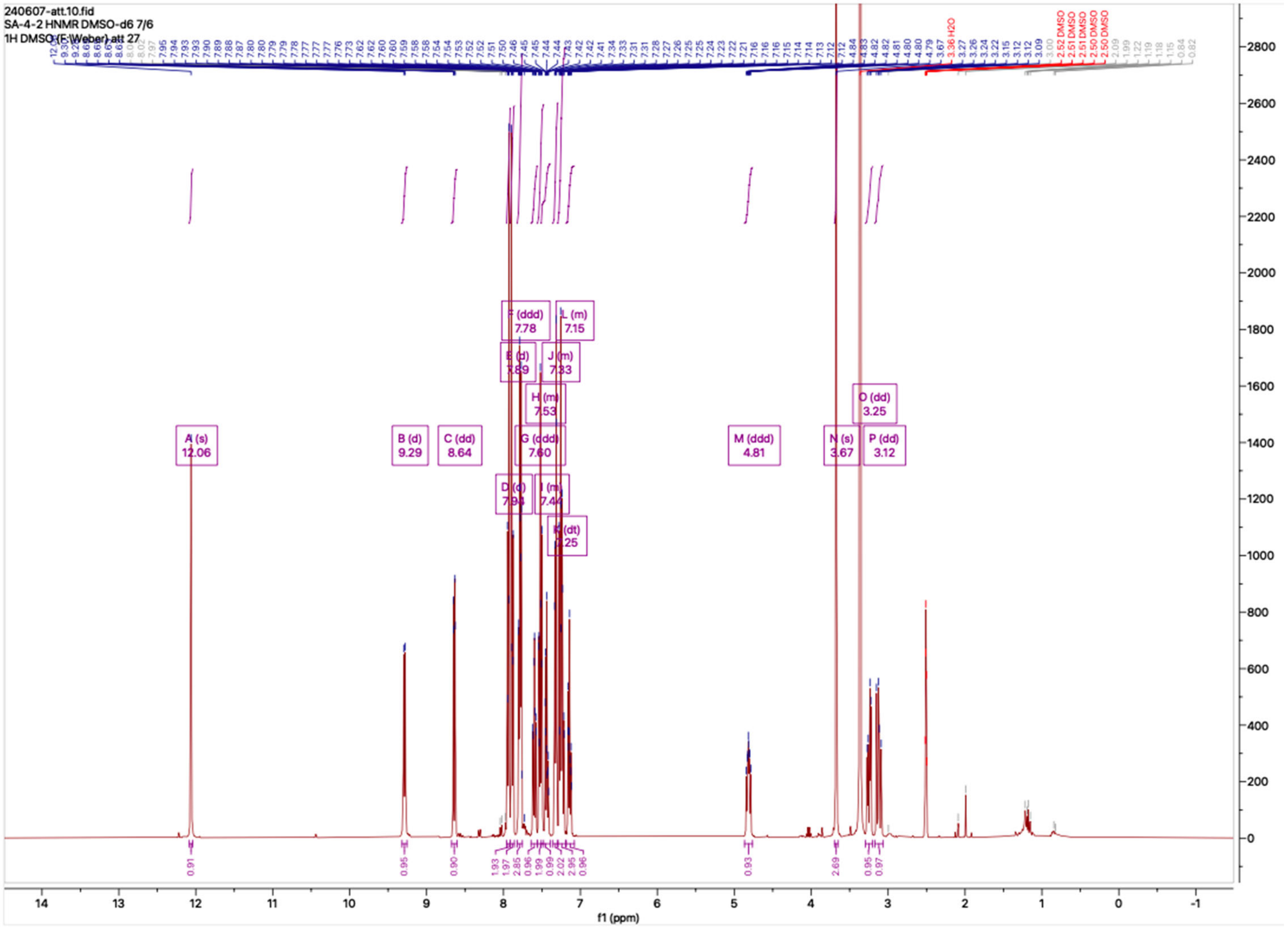

$^{13}$C NMR of Methyl (2-([1,1'-biphenyl]-4-carboxamido)benzoyl)-*L*-phenylalaninate (15g)

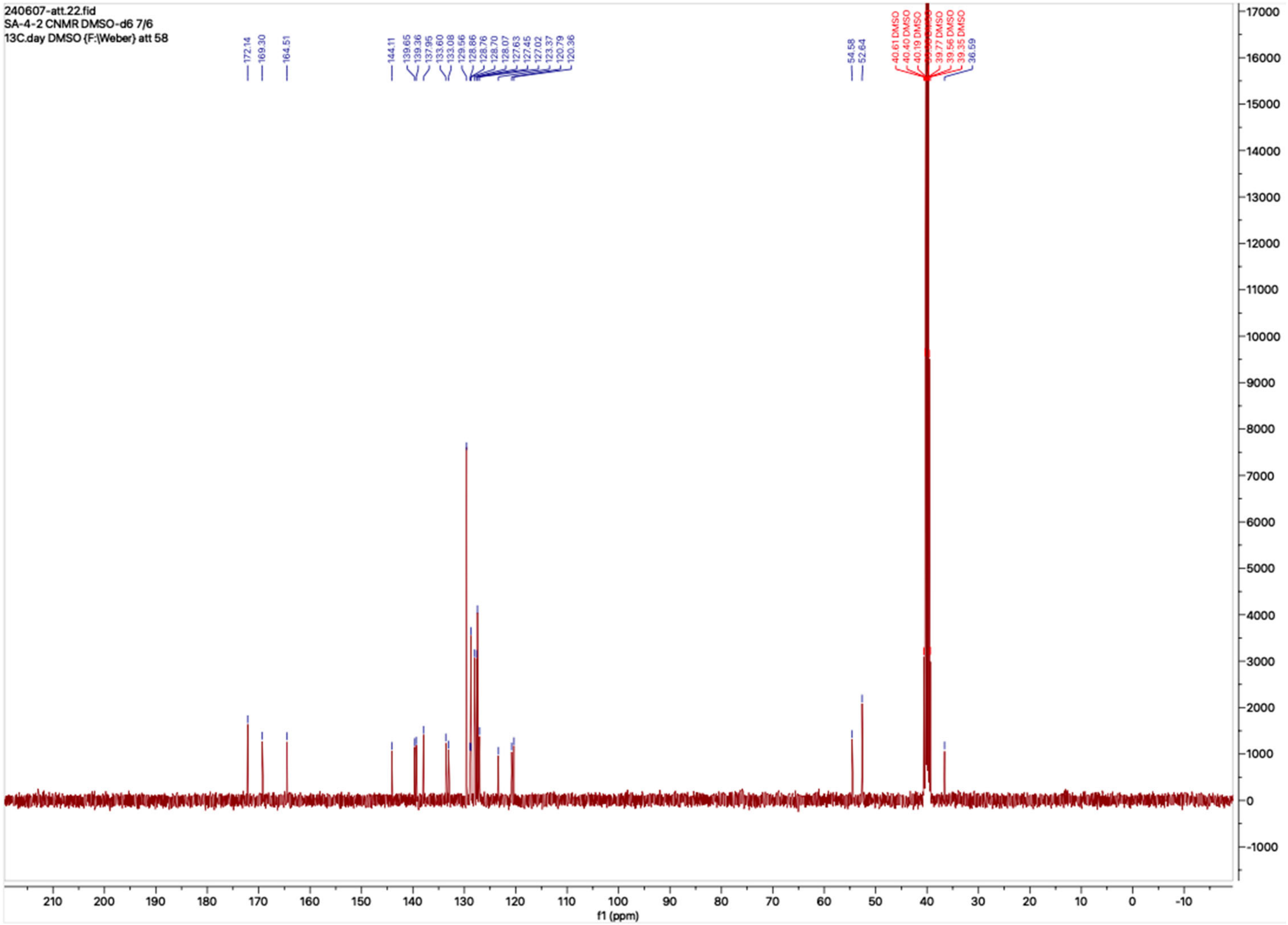

$^{1}$H NMR of Methyl (2-([1,1'-biphenyl]-4-carboxamido)benzoyl)-*L*-tryptophanate (15h)

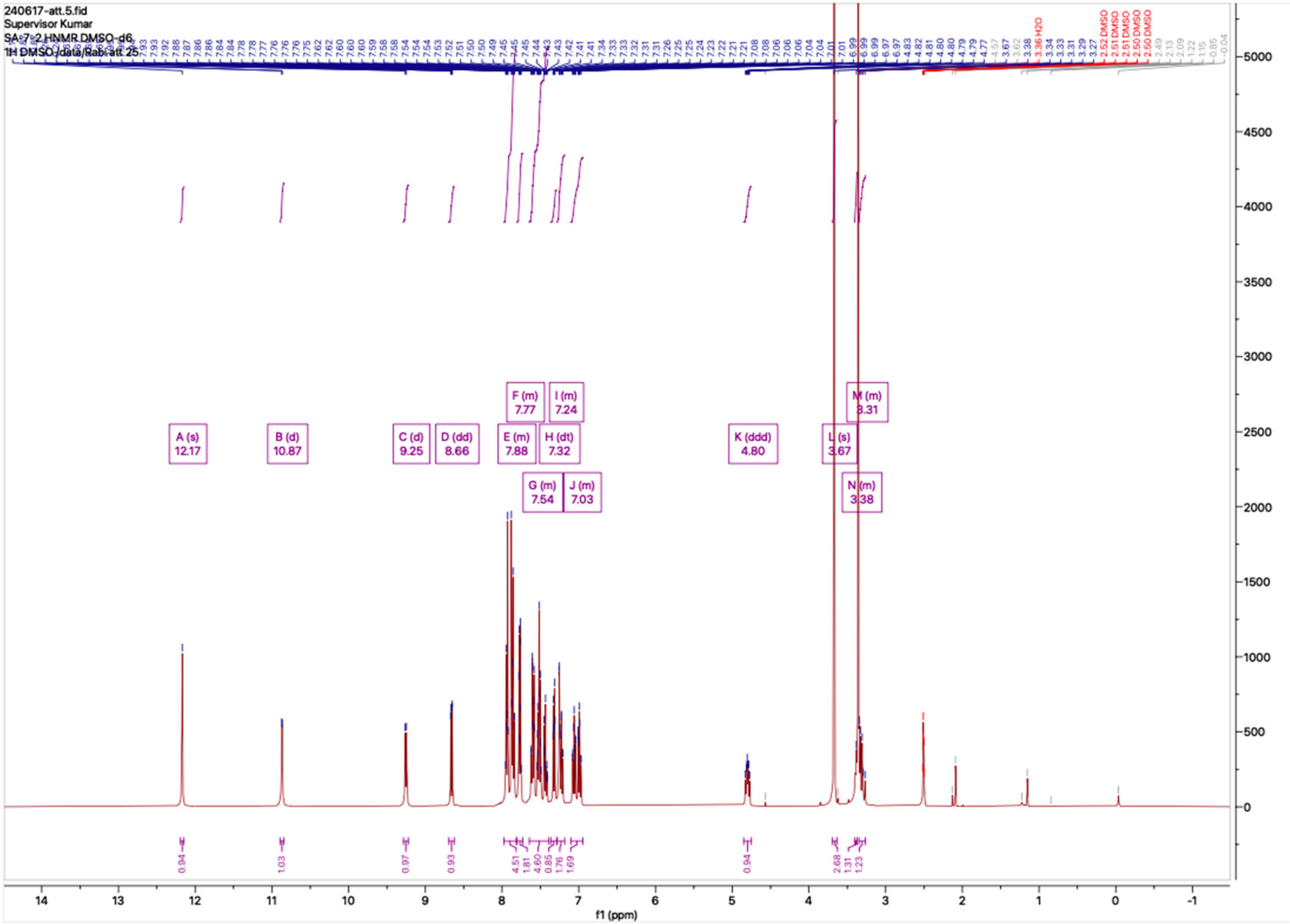

$^{13}$C NMR of Methyl (2-([1,1'-biphenyl]-4-carboxamido)benzoyl)-*L*-tryptophanate (15h)

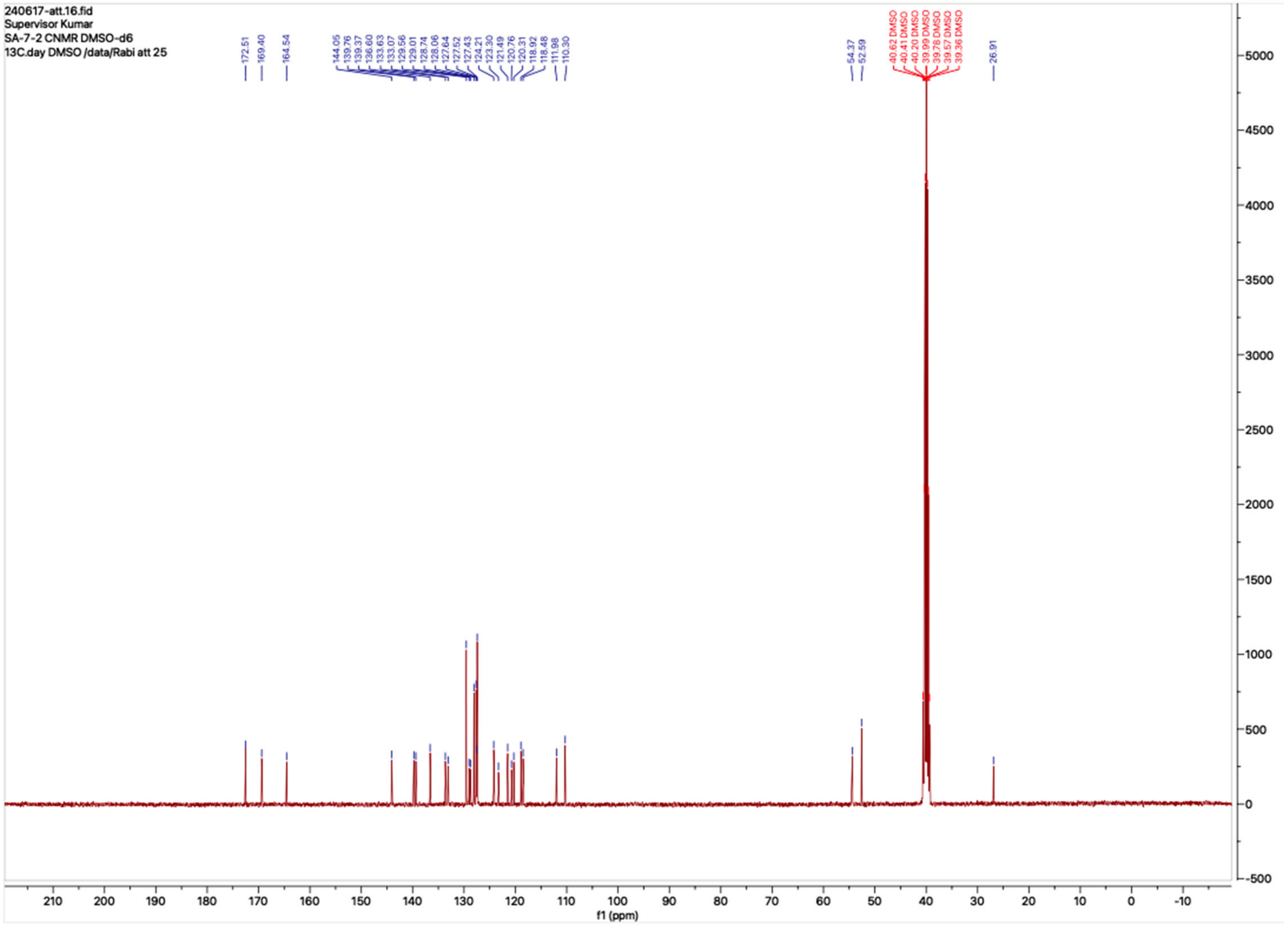

$^{1}$H NMR of (2-([1,1'-biphenyl]-2-carboxamido)benzoyl)-*L*-phenylalanine (16e)

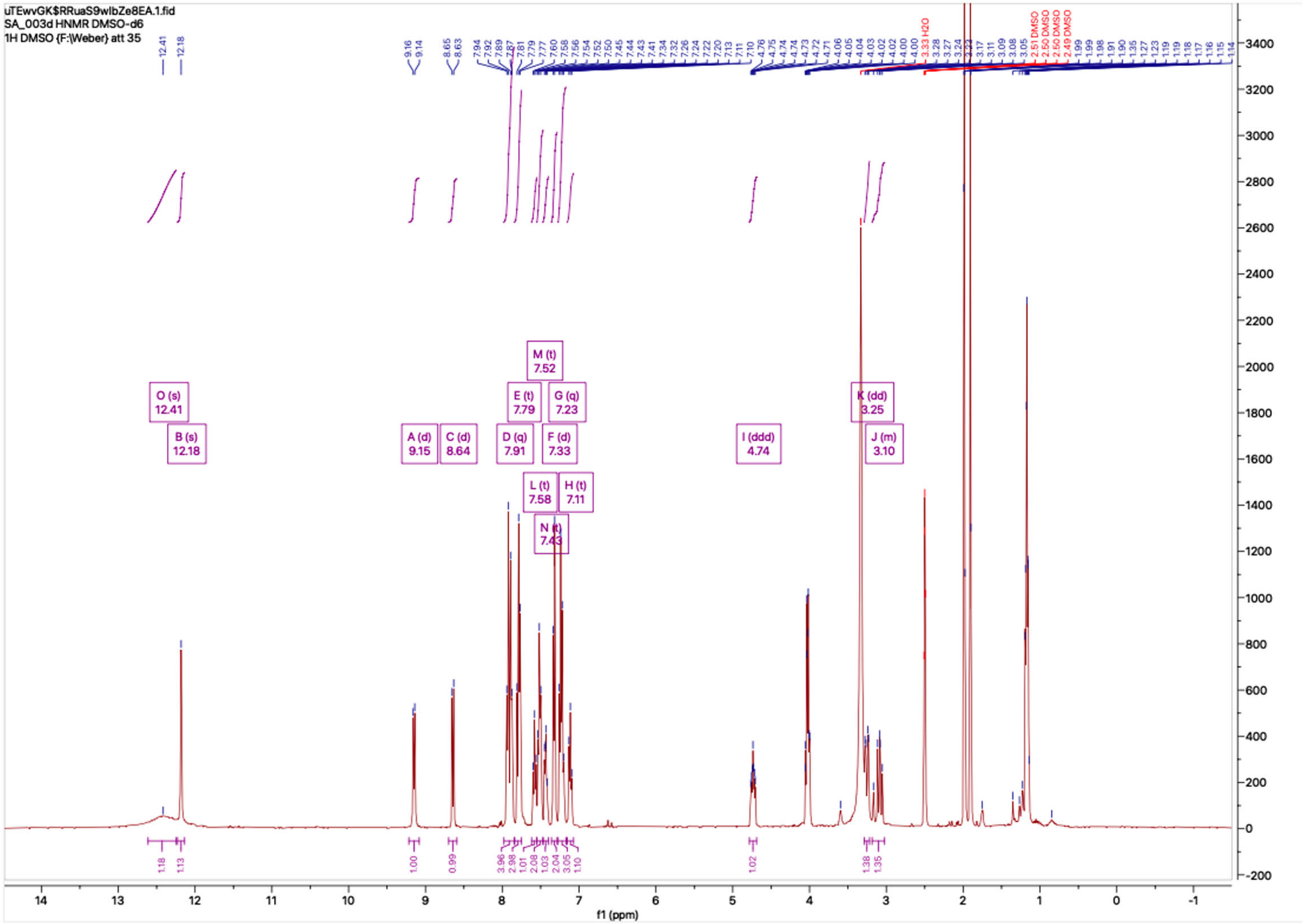

$^{13}$C NMR of (2-([1,1'-biphenyl]-2-carboxamido)benzoyl)-*L*-phenylalanine (16e)

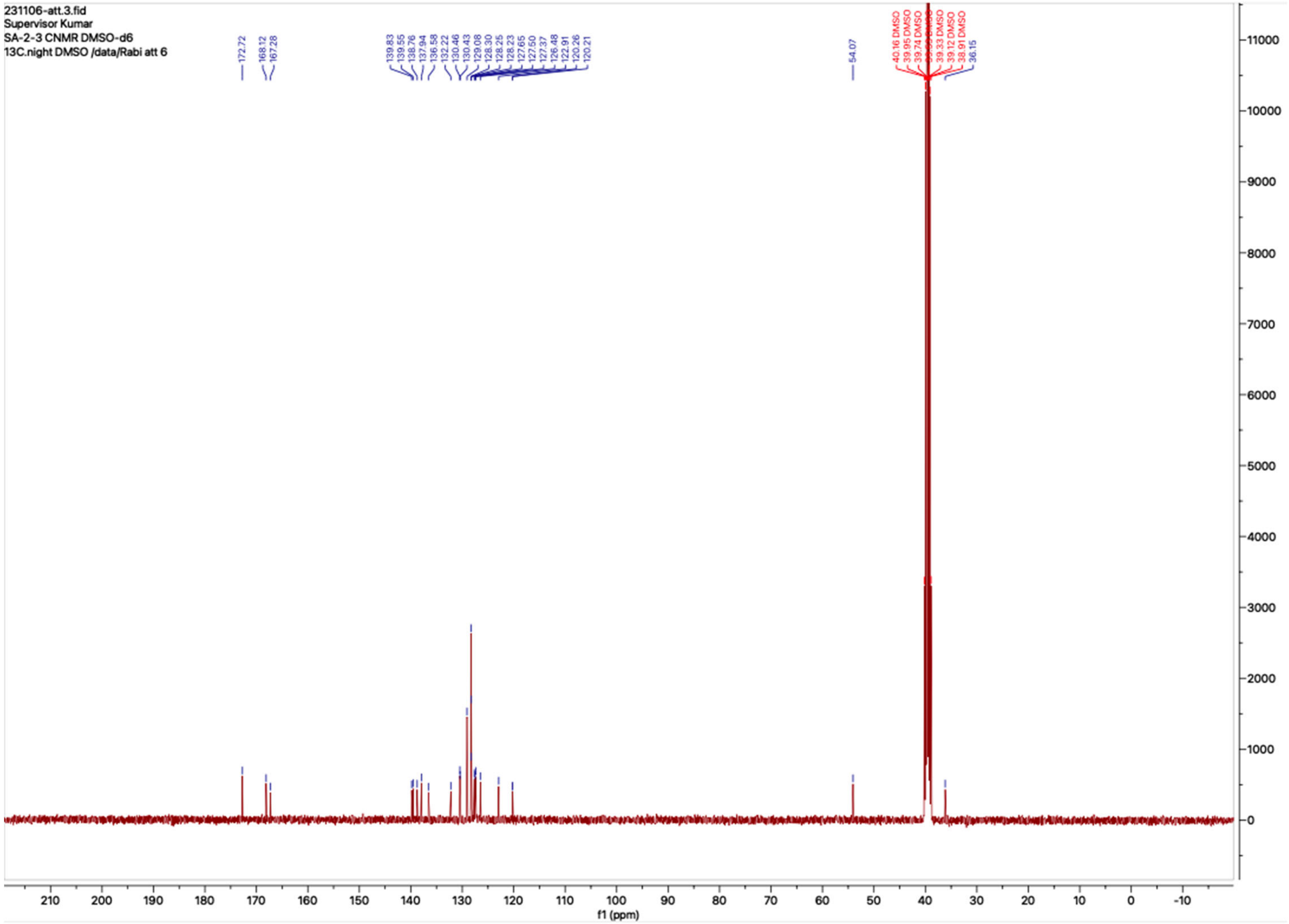

$^{1}$H NMR of (2-([1,1'-biphenyl]-3-carboxamido)benzoyl)-*L*-phenylalanine (16f)

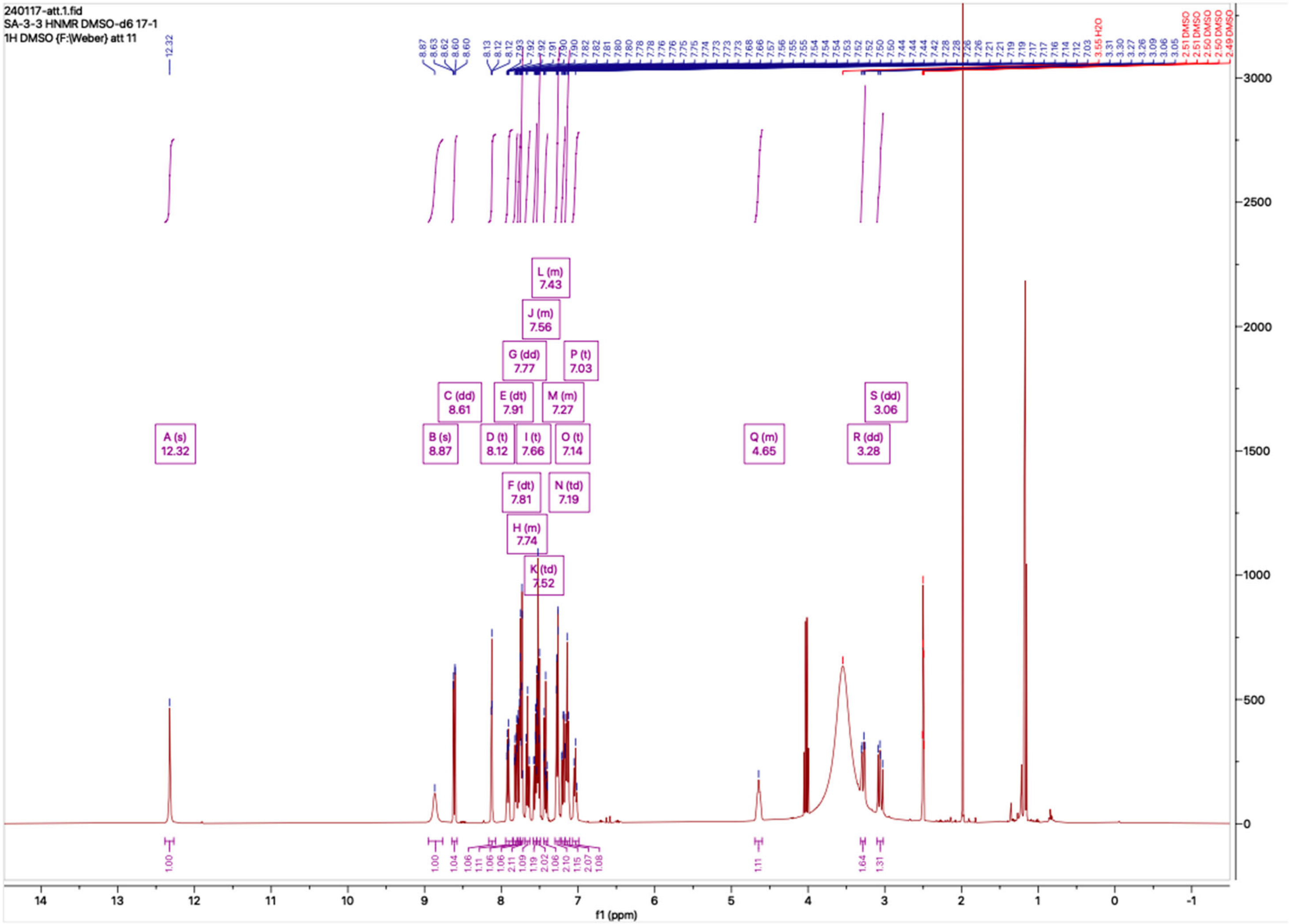

$^{13}$C NMR of (2-([1,1'-biphenyl]-3-carboxamido)benzoyl)-*L*-phenylalanine (16f)

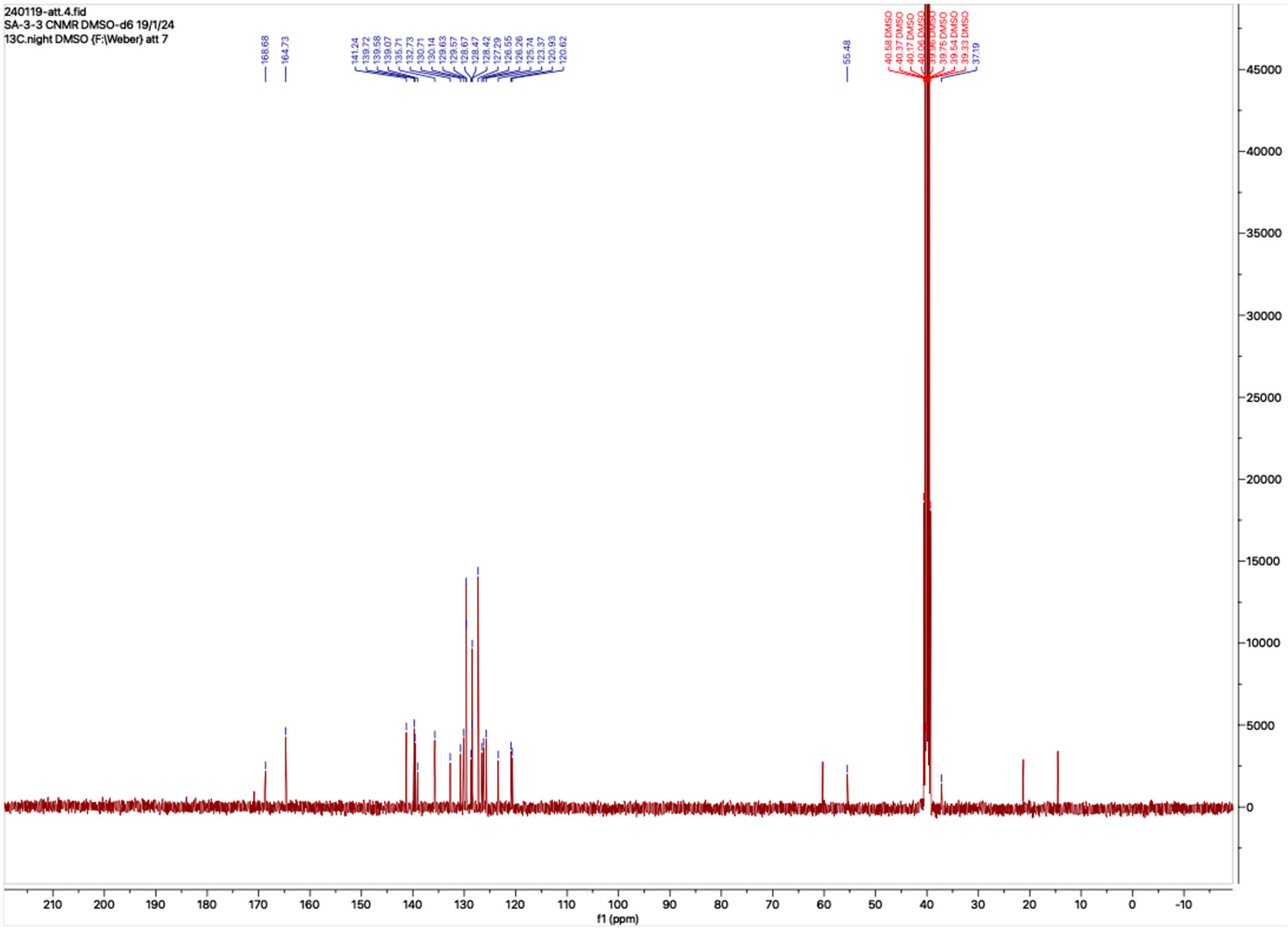

$^{1}$H NMR of (2-([1,1'-biphenyl]-4-carboxamido)benzoyl)-*L*-phenylalanine (16g)

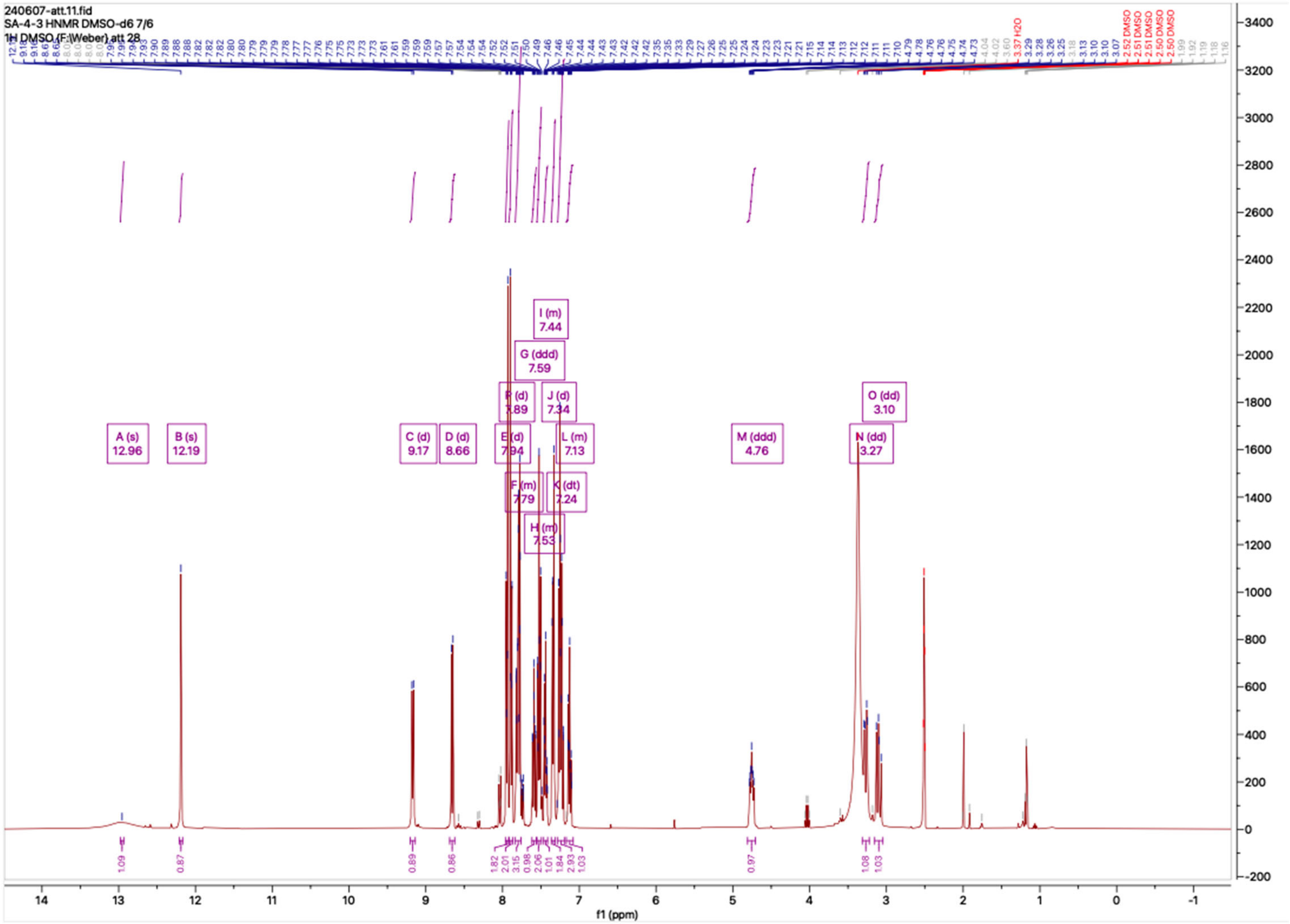

$^{13}$C NMR of (2-([1,1'-biphenyl]-4-carboxamido)benzoyl)-*L*-phenylalanine (16g)

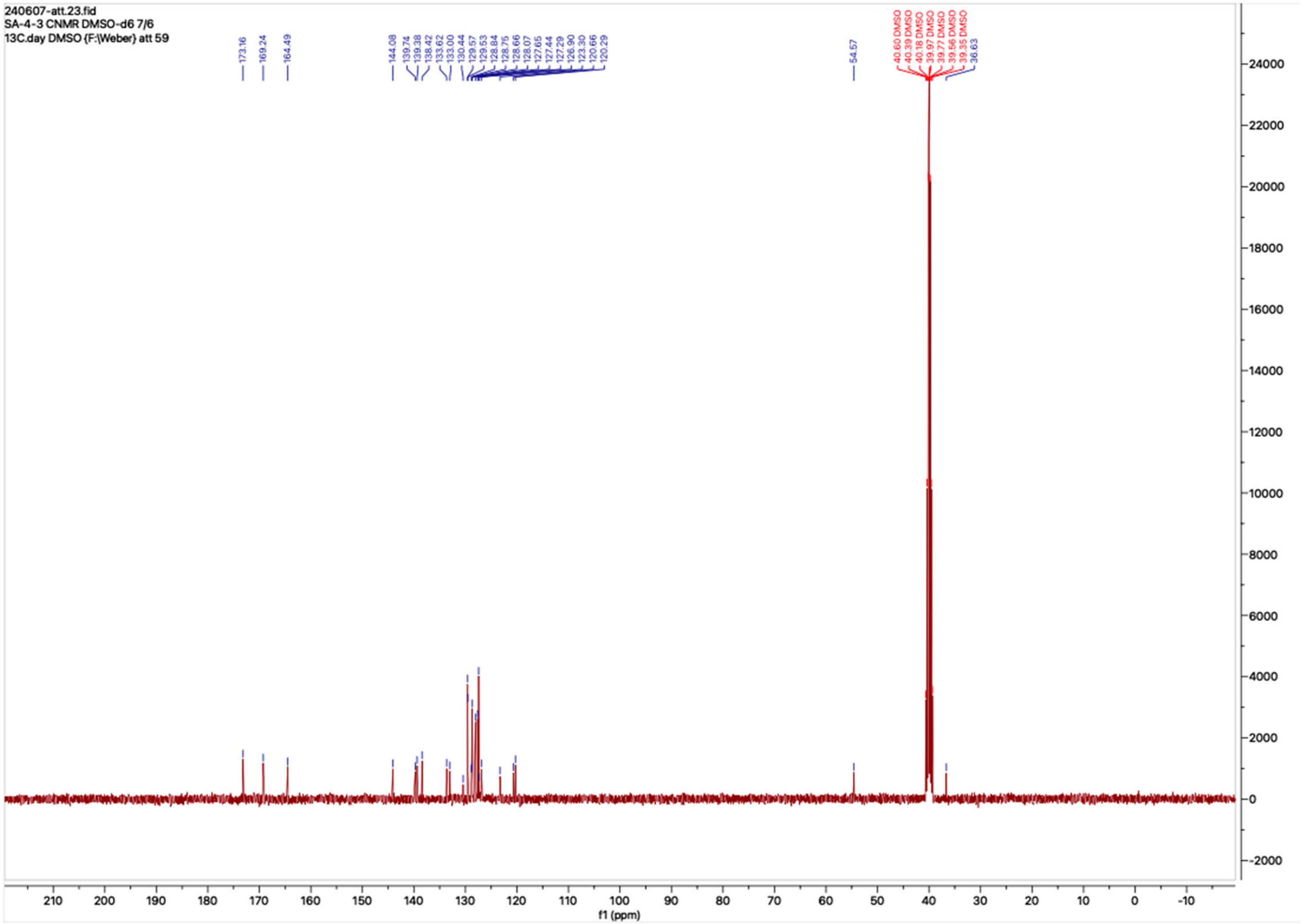

$^{1}$H NMR of (2-([1,1'-biphenyl]-4-carboxamido)benzoyl)-*L*-tryptophan (16h)

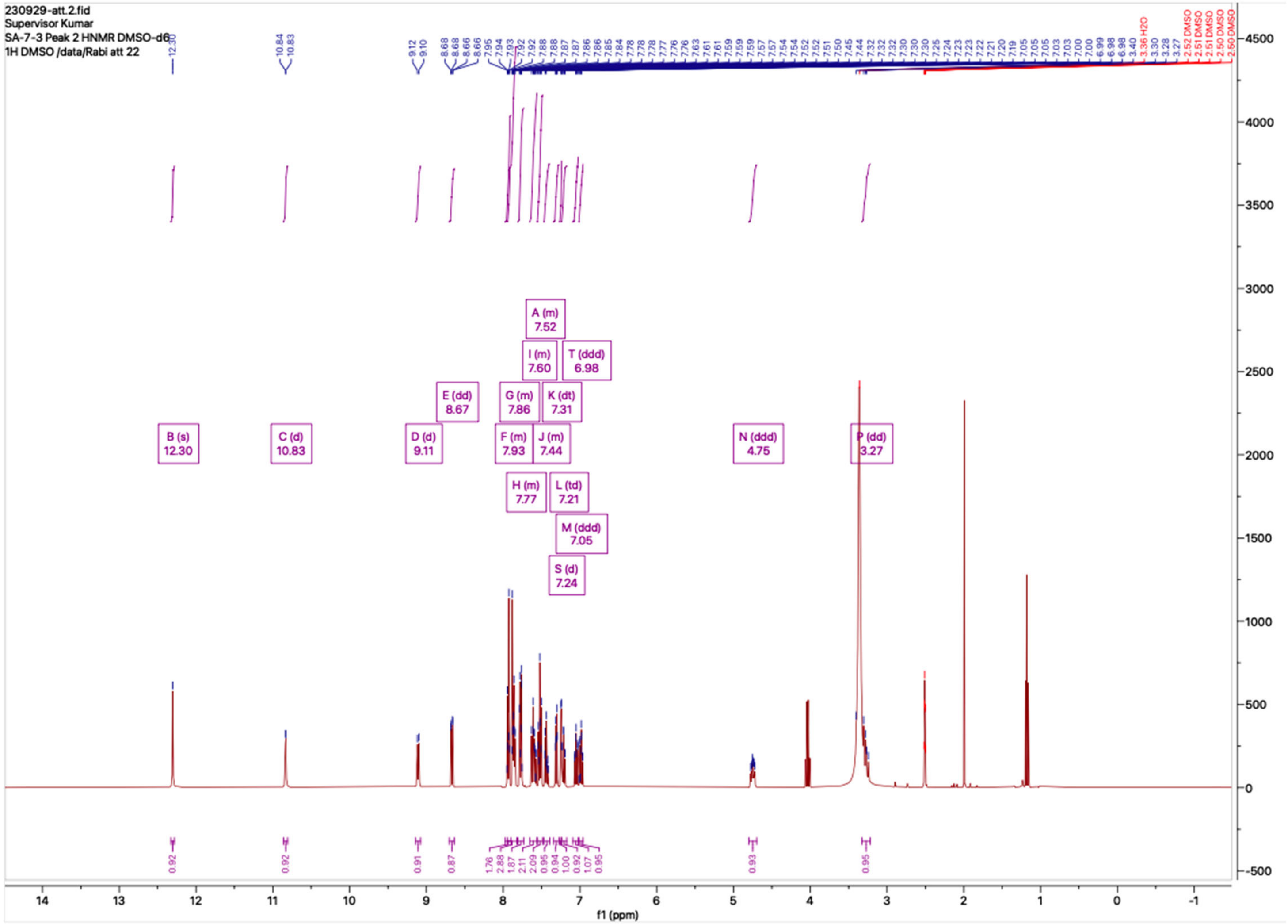

$^{13}$C NMR of (2-([1,1'-biphenyl]-4-carboxamido)benzoyl)-*L*-tryptophan (16h)

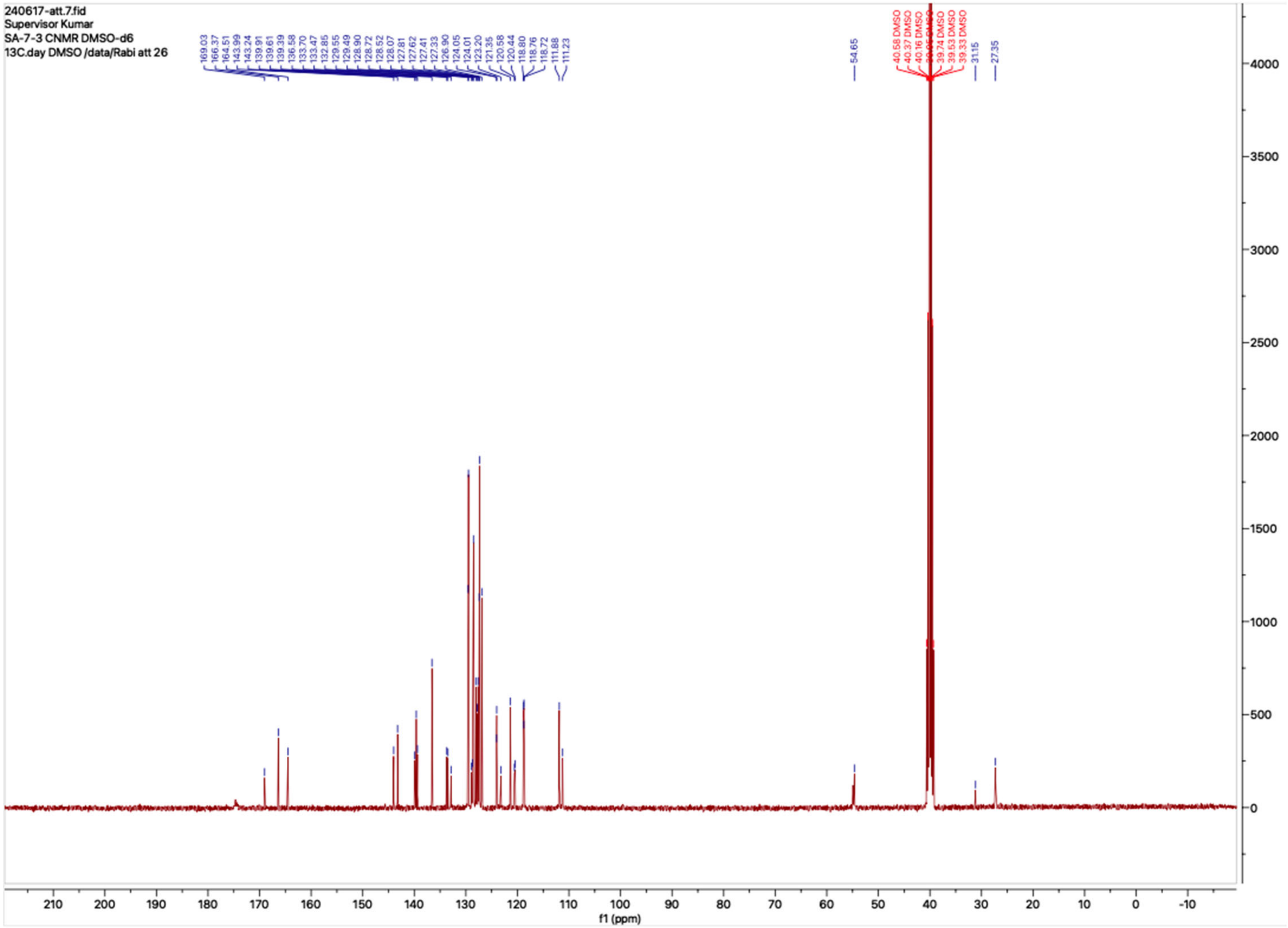

<sup></sup>

$^{1}$H NMR of *Tert*-butyl (*S*)-(3-(2-(2-(2-naphthamido)benzamido)-3-(1*H*-indol-3-yl)propanamido)propyl)carbamate (17b)

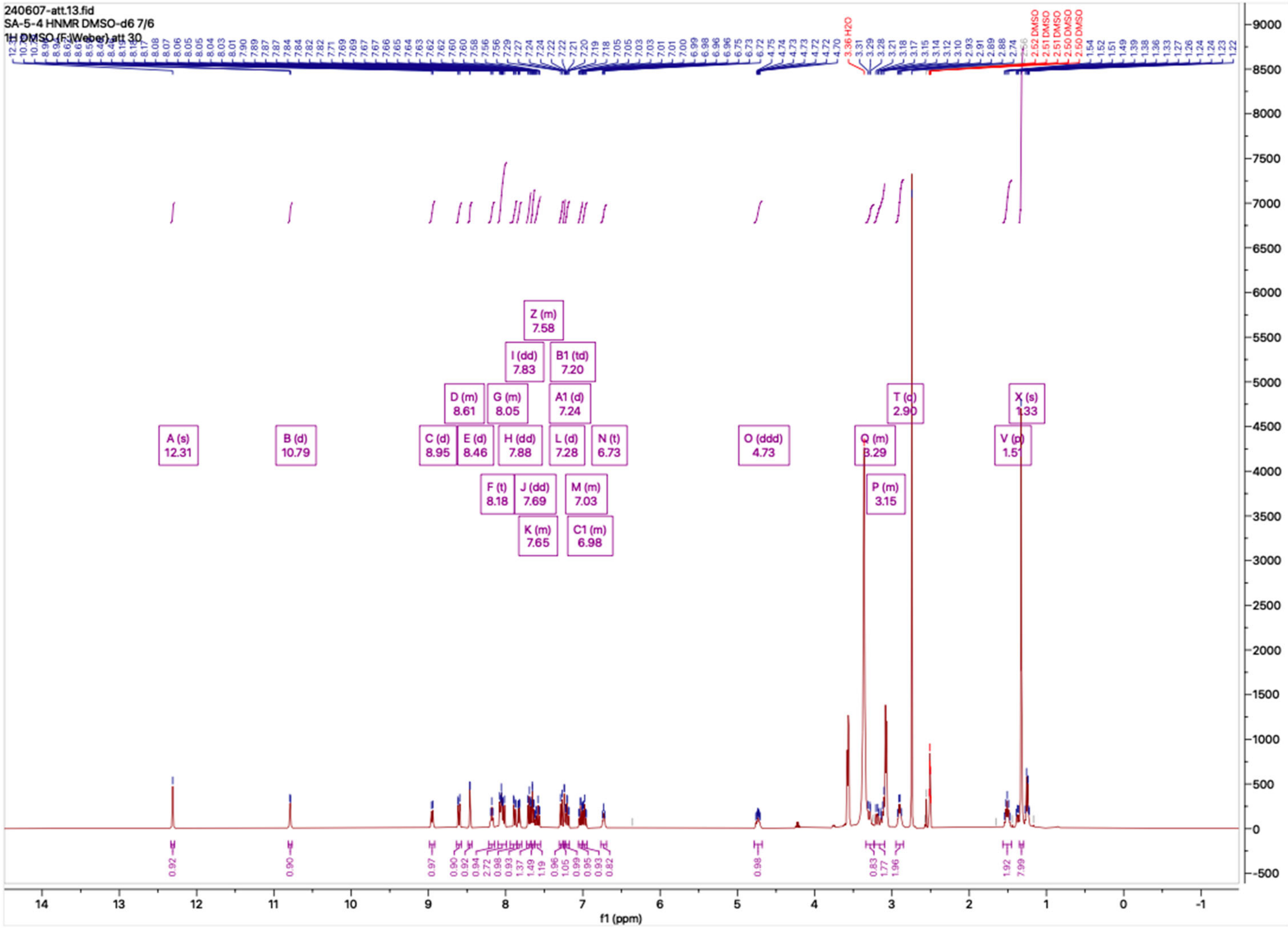

$^{13}C$ NMR of *Tert*-butyl (*S*)-(3-(2-(2-(2-naphthamido)benzamido)-3-(1*H*-indol-3-yl)propanamido)propyl)carbamate (17b)

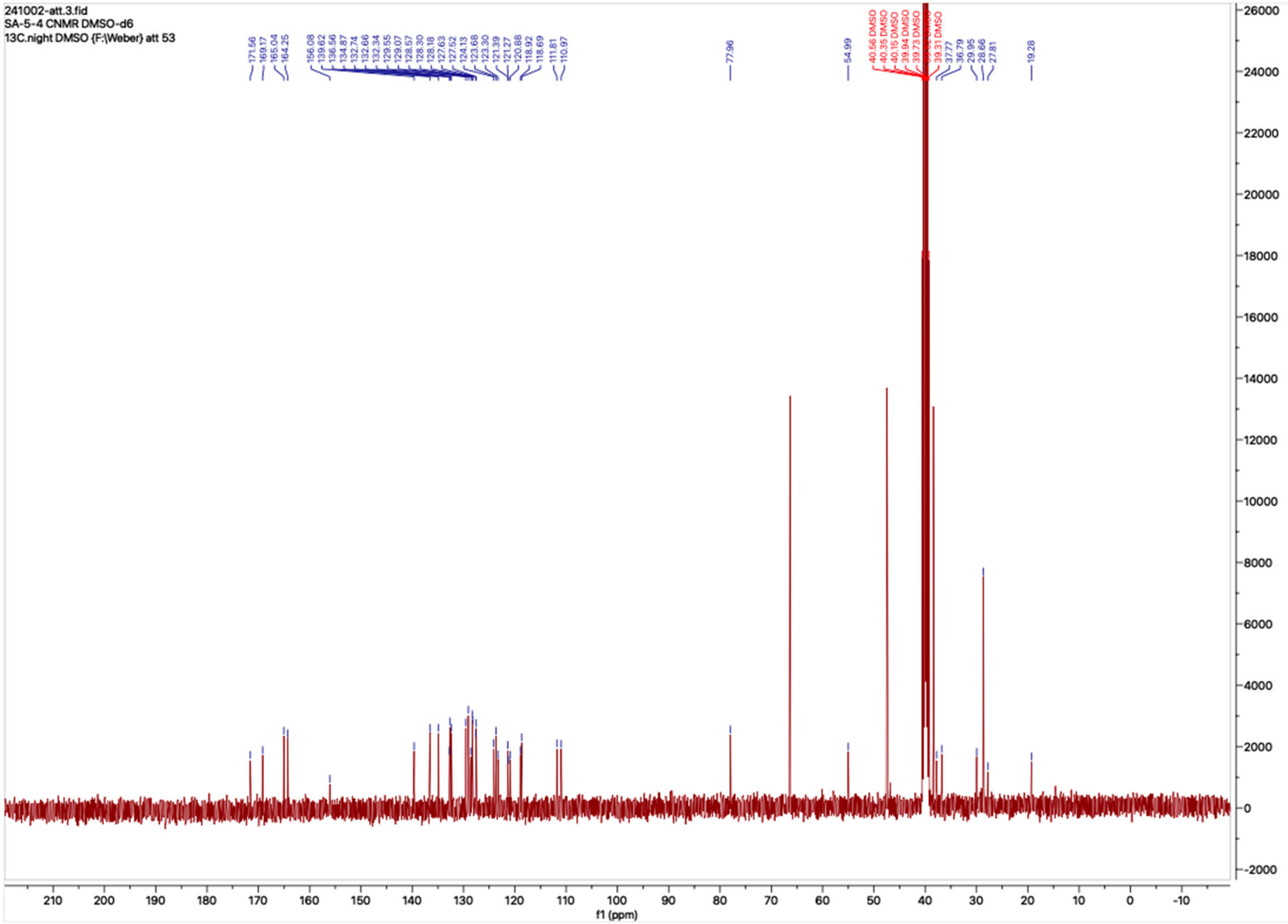

$^{1}$H NMR of *Tert*-butyl (*S*)-(3-(2-(2-(2-naphthamido)-5-bromobenzamido)-3-phenylpropanamido)propyl)carbamate (17c)

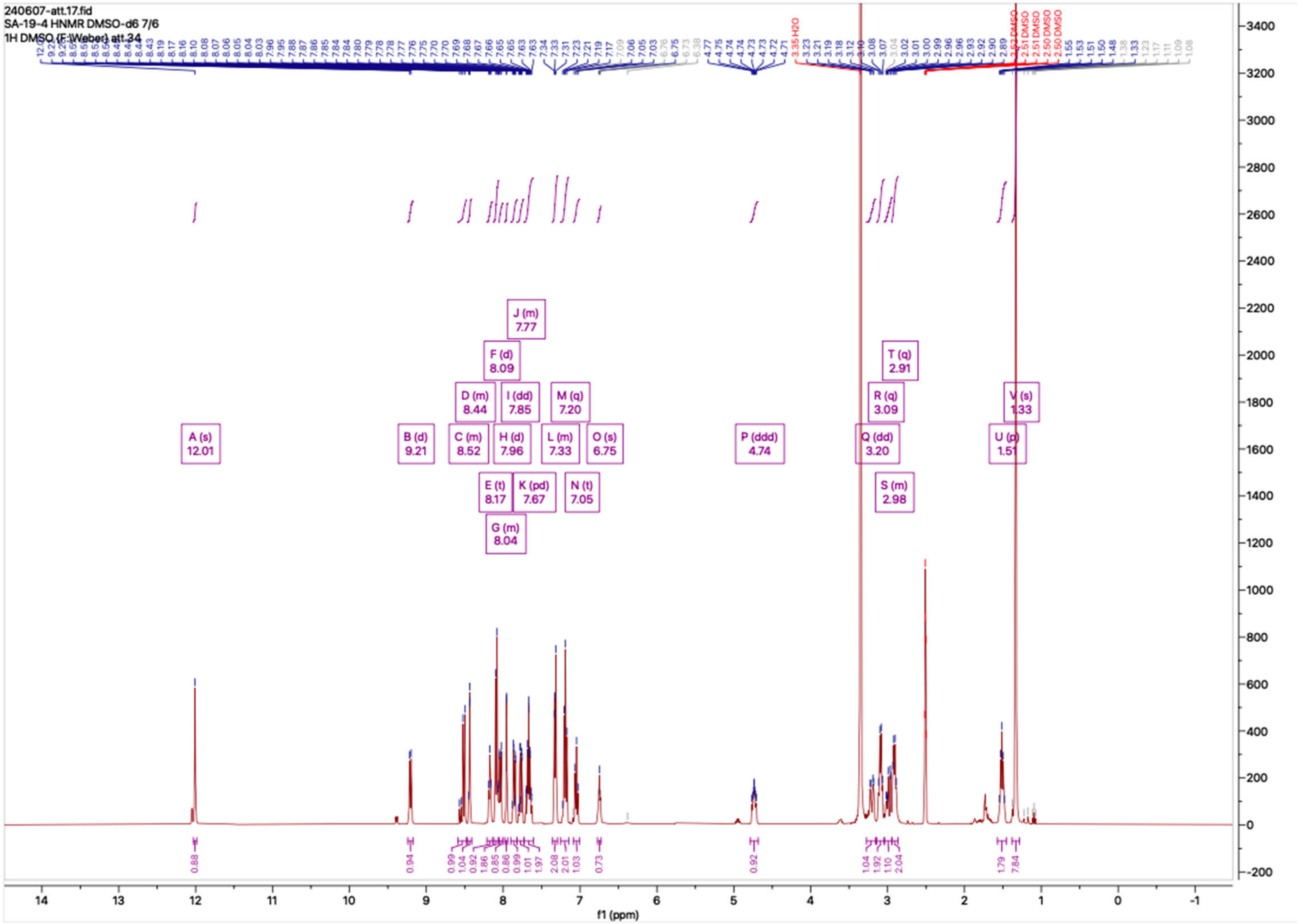

$^{13}$C NMR of *Tert*-butyl (*S*)-(3-(2-(2-(2-naphthamido)-5-bromobenzamido)-3-phenylpropanamido)propyl)carbamate (17c)

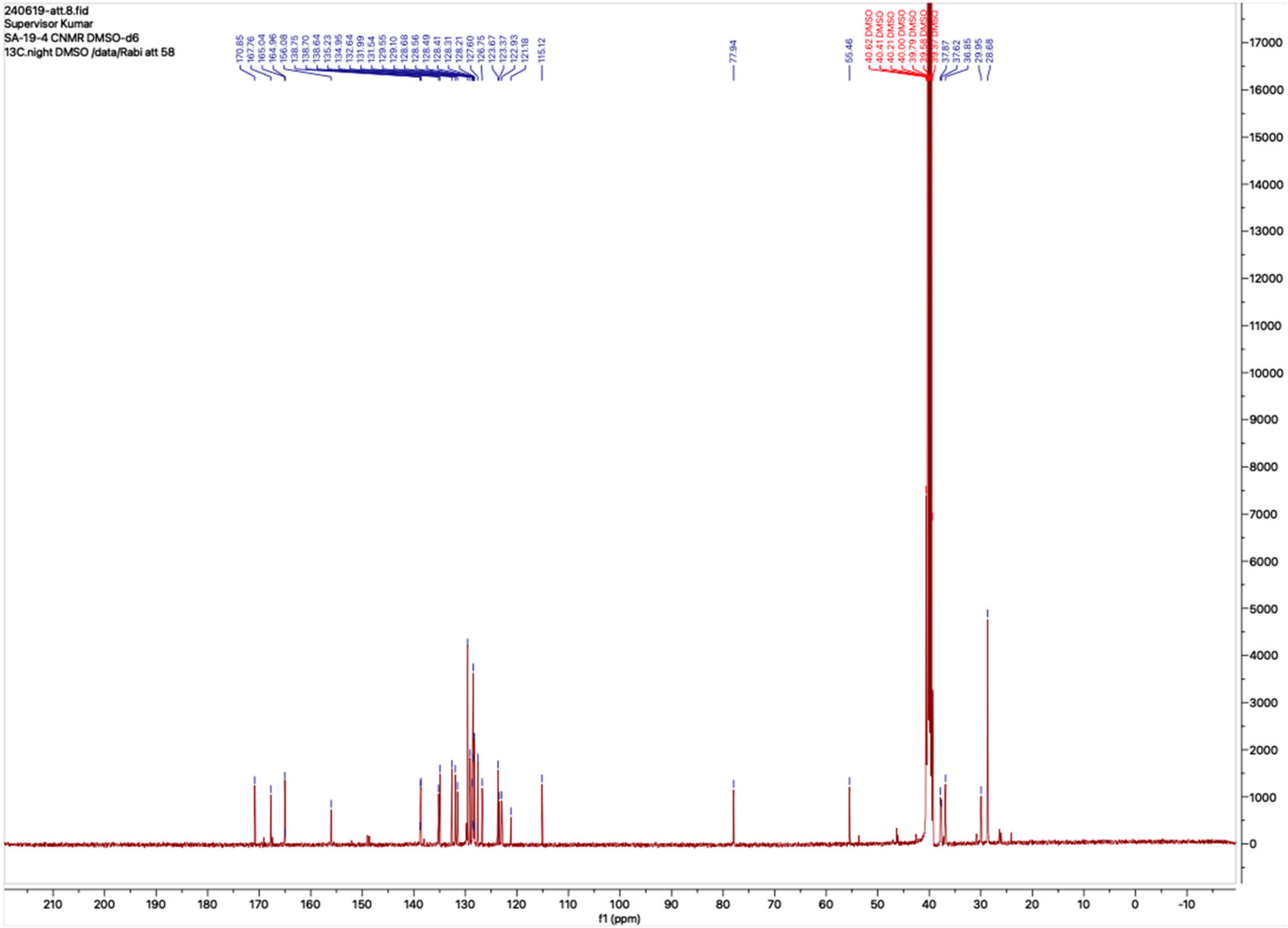

$^{1}$H NMR of *Tert*-butyl (*S*)-(3-(2-(2-(2-naphthamido)-5-bromobenzamido)-3-(1*H*-indol-3-yl)propanamido)propyl)carbamate (17d)

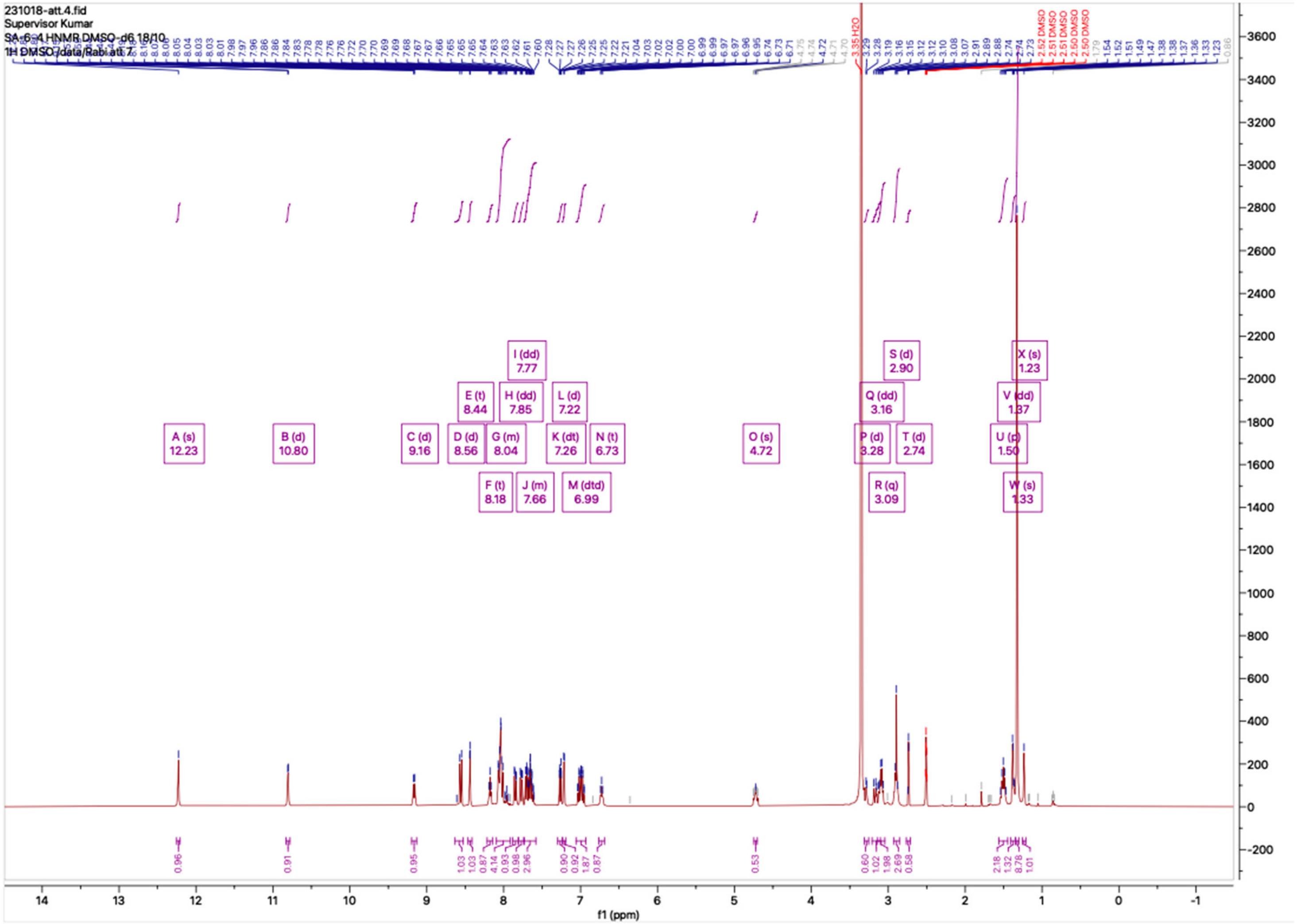

$^{13}$C NMR of *Tert*-butyl (*S*)-(3-(2-(2-(2-naphthamido)-5-bromobenzamido)-3-(1*H*-indol-3-yl)propanamido)propyl)carbamate (17d)

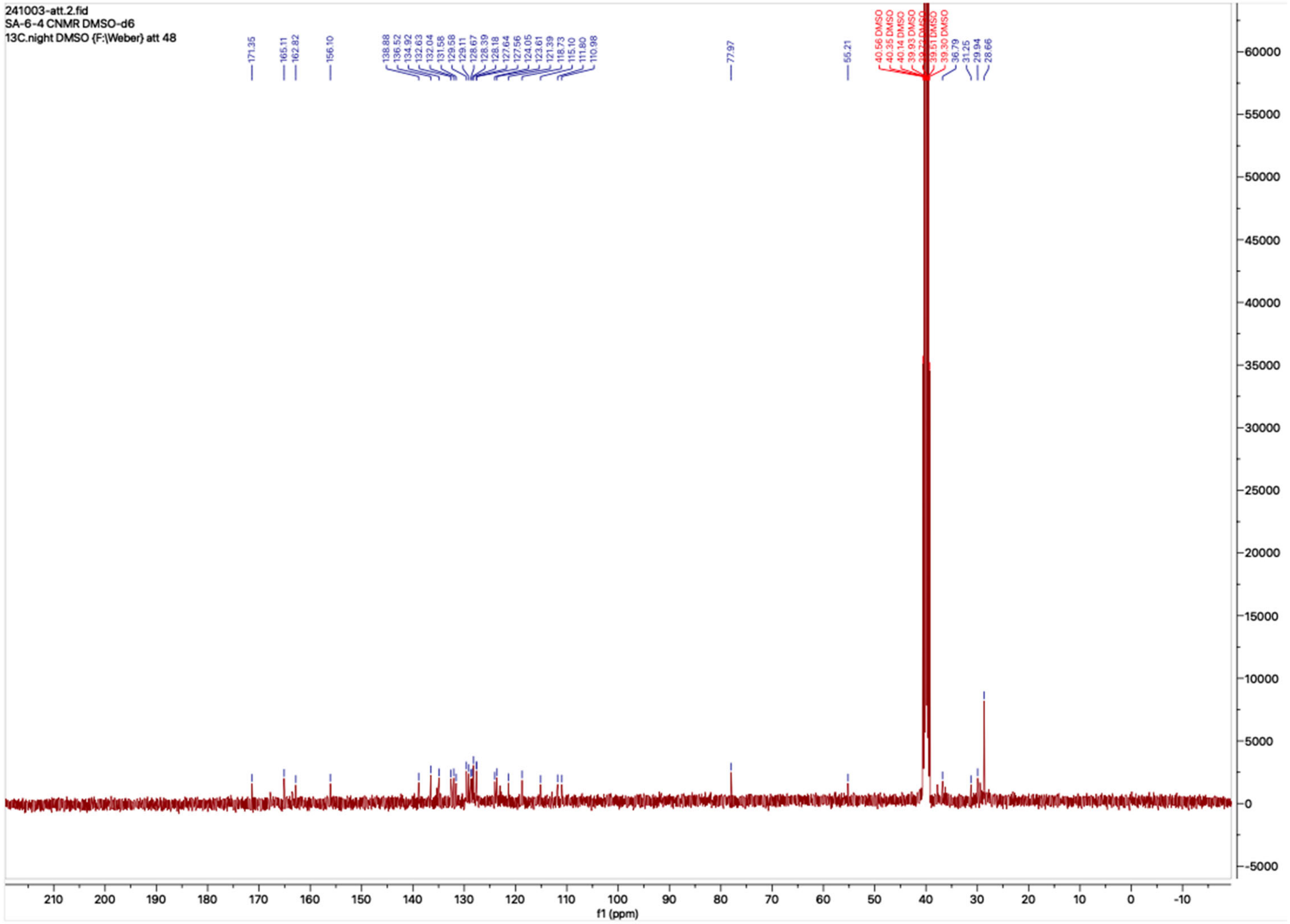

$^{1}$H NMR of *Tert*-butyl (*S*)-(3-(2-(2-([1,1'-biphenyl]-2-carboxamido)benzamido)-3-phenylpropanamido)propyl)carbamate (17e)

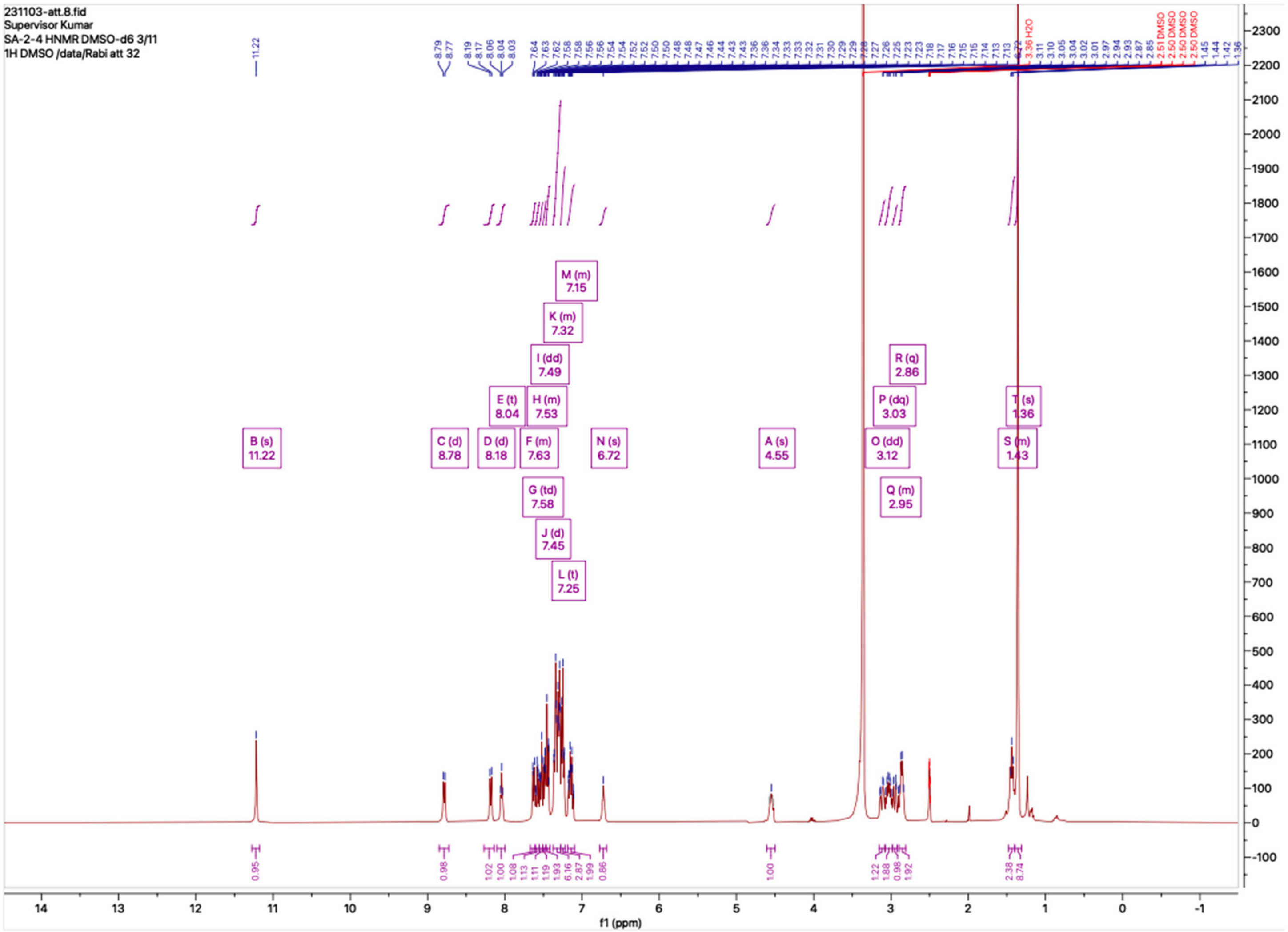

$^{13}$C NMR of *Tert*-butyl (*S*)-(3-(2-(2-([1,1'-biphenyl]-2-carboxamido)benzamido)-3-phenylpropanamido)propyl)carbamate (17e)

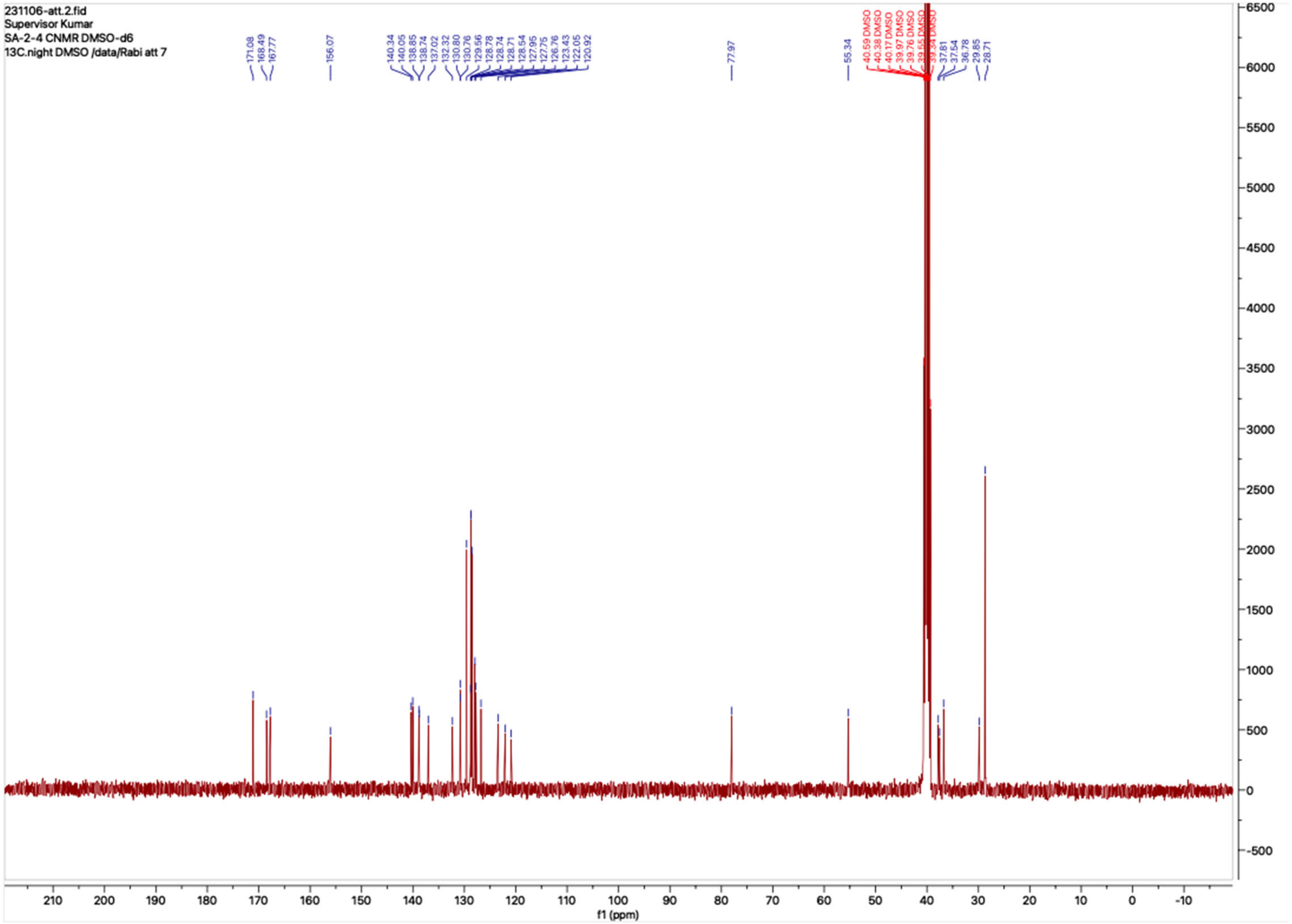

$^{1}$H NMR of *Tert*-butyl (*S*)-(3-(2-(2-([1,1'-biphenyl]-3-carboxamido)benzamido)-3-phenylpropanamido)propyl)carbamate (17f)

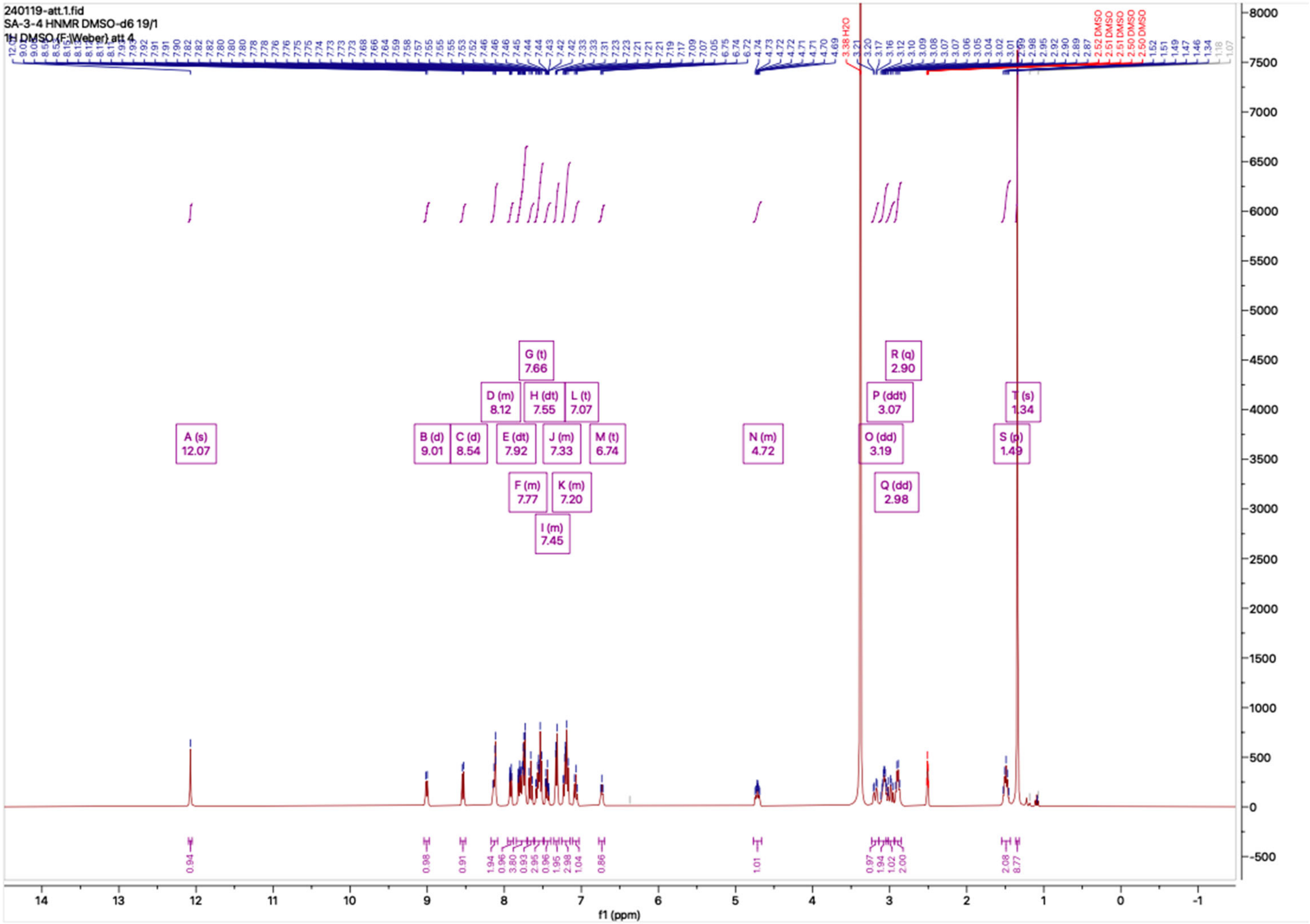

$^{13}$C NMR of *Tert*-butyl (*S*)-(3-(2-(2-([1,1'-biphenyl]-3-carboxamido)benzamido)-3-phenylpropanamido)propyl)carbamate (17f)

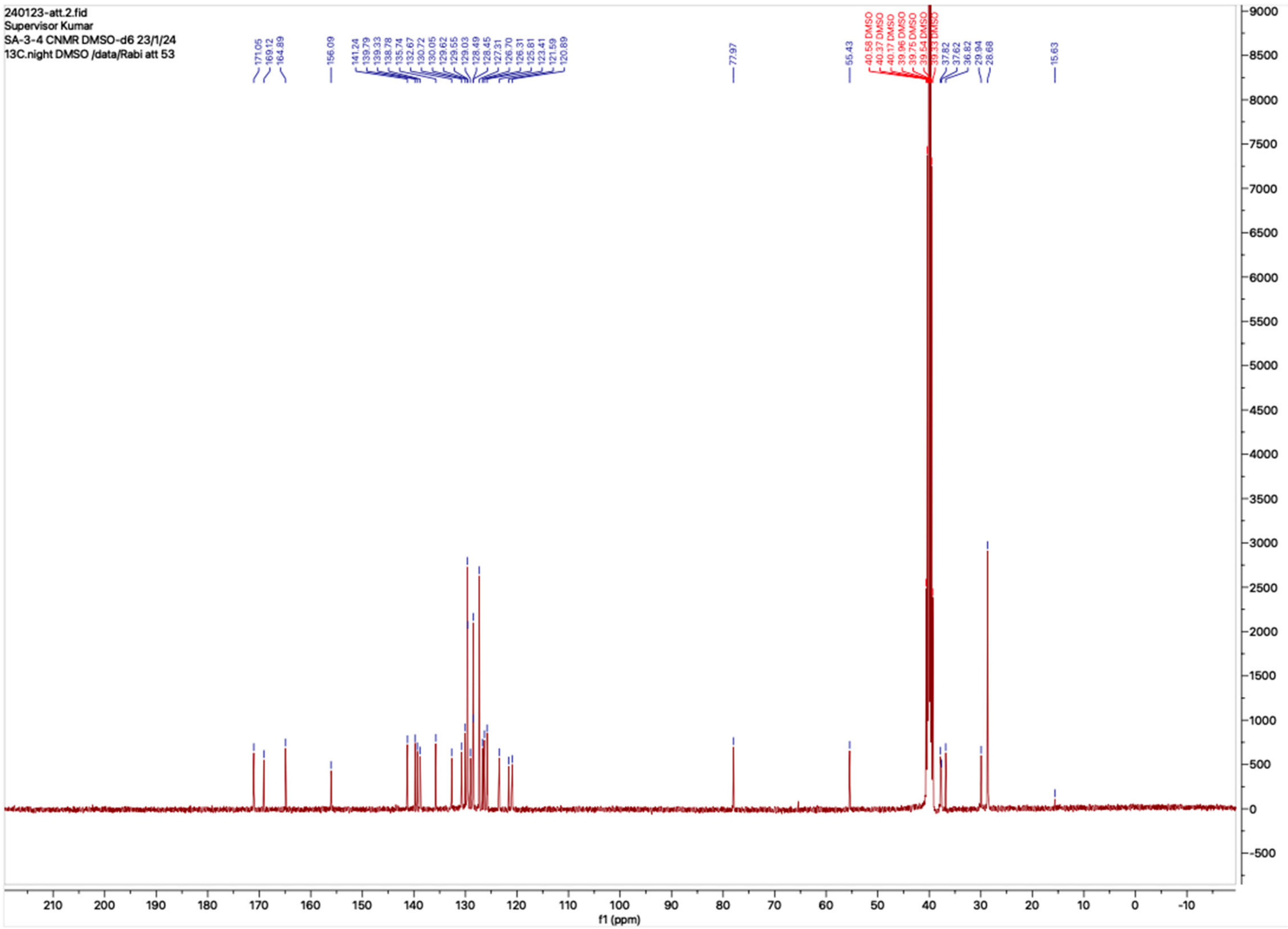

$^{1}$H NMR of *Tert*-butyl (*S*)-(3-(2-(2-([1,1'-biphenyl]-4-carboxamido)benzamido)-3-phenylpropanamido)propyl)carbamate (17g)

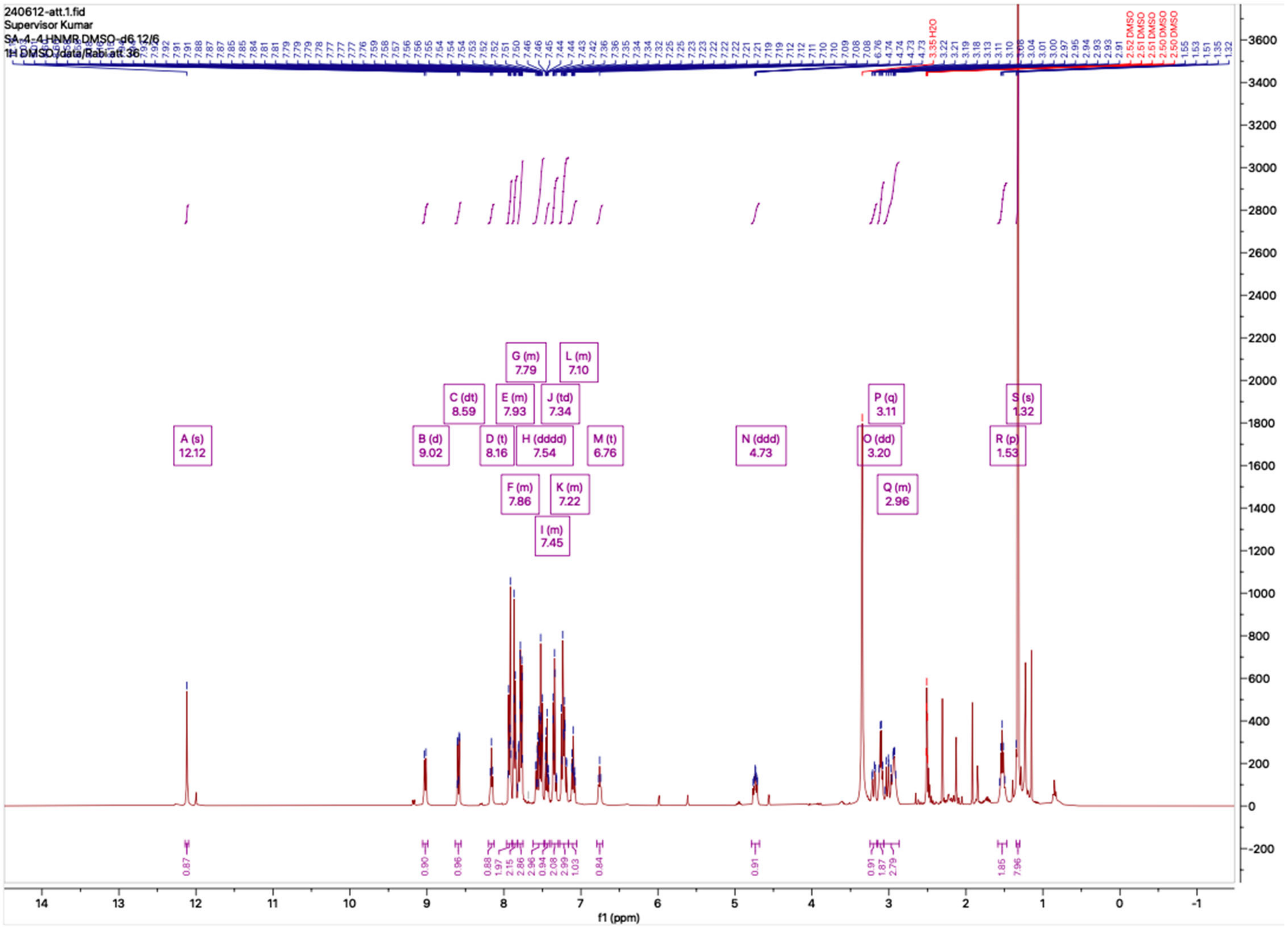

$^{13}$C NMR of *Tert*-butyl (*S*)-(3-(2-(2-([1,1'-biphenyl]-4-carboxamido)benzamido)-3-phenylpropanamido)propyl)carbamate (17g)

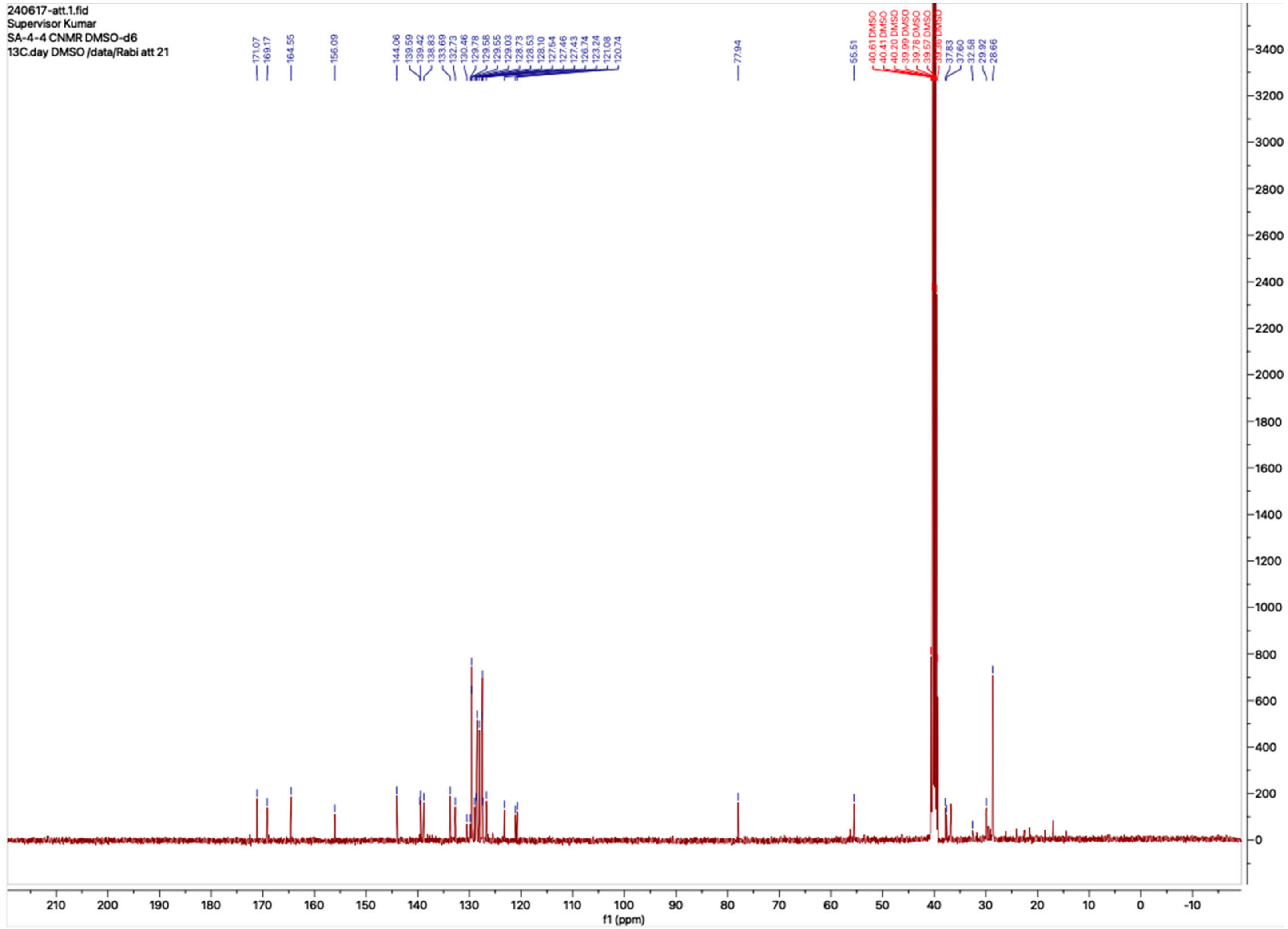

$^{1}$H NMR of *Tert*-butyl (*S*)-(3-(2-(2-([1,1'-biphenyl]-4-carboxamido)benzamido)-3-(1*H*-indol-3-yl)propanamido)propyl)carbamate (17h)

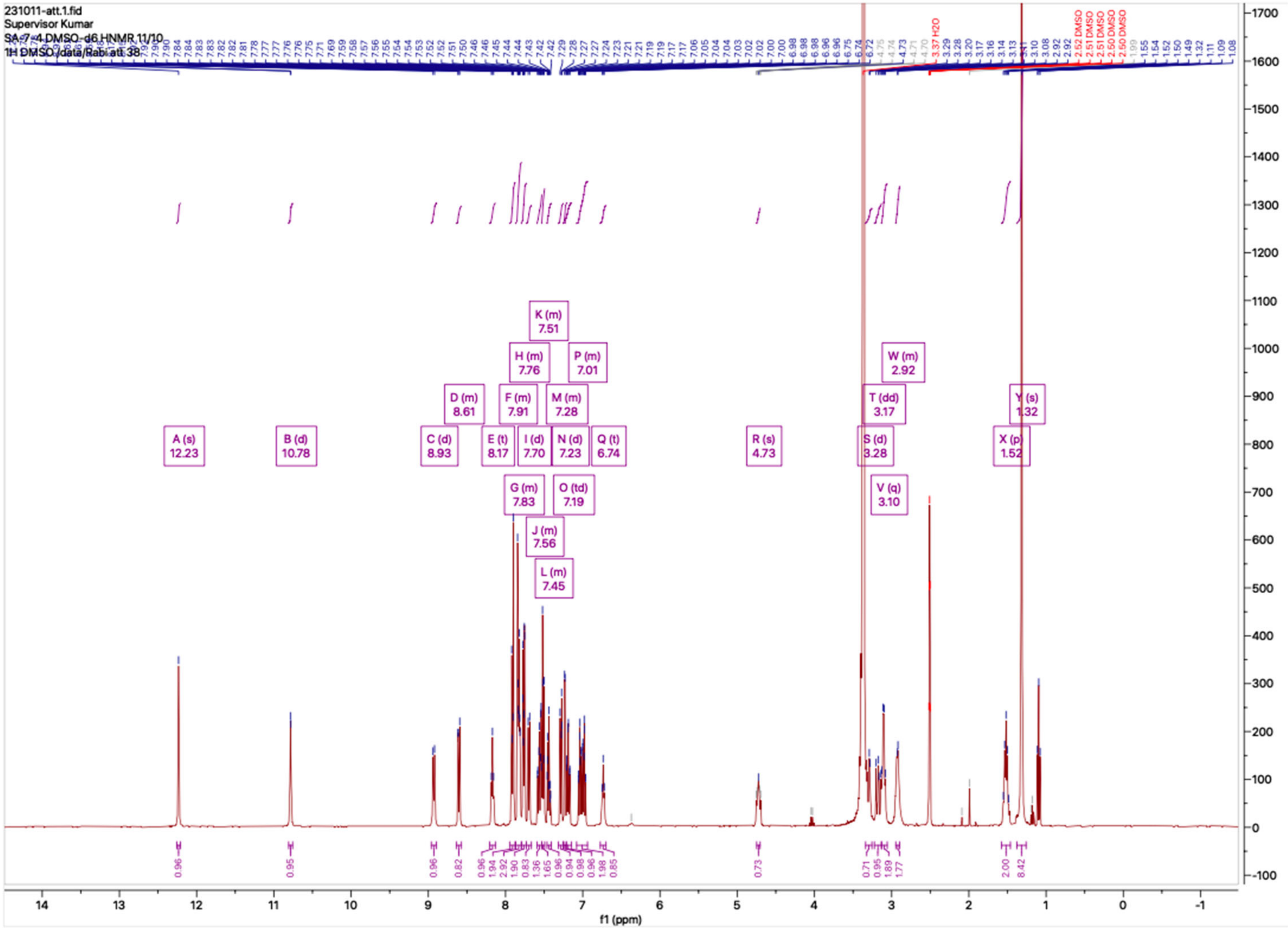

$^{13}$C NMR of *Tert*-butyl (*S*)-(3-(2-(2-([1,1'-biphenyl]-4-carboxamido)benzamido)-3-(1*H*-indol-3-yl)propanamido)propyl)carbamate (17h)

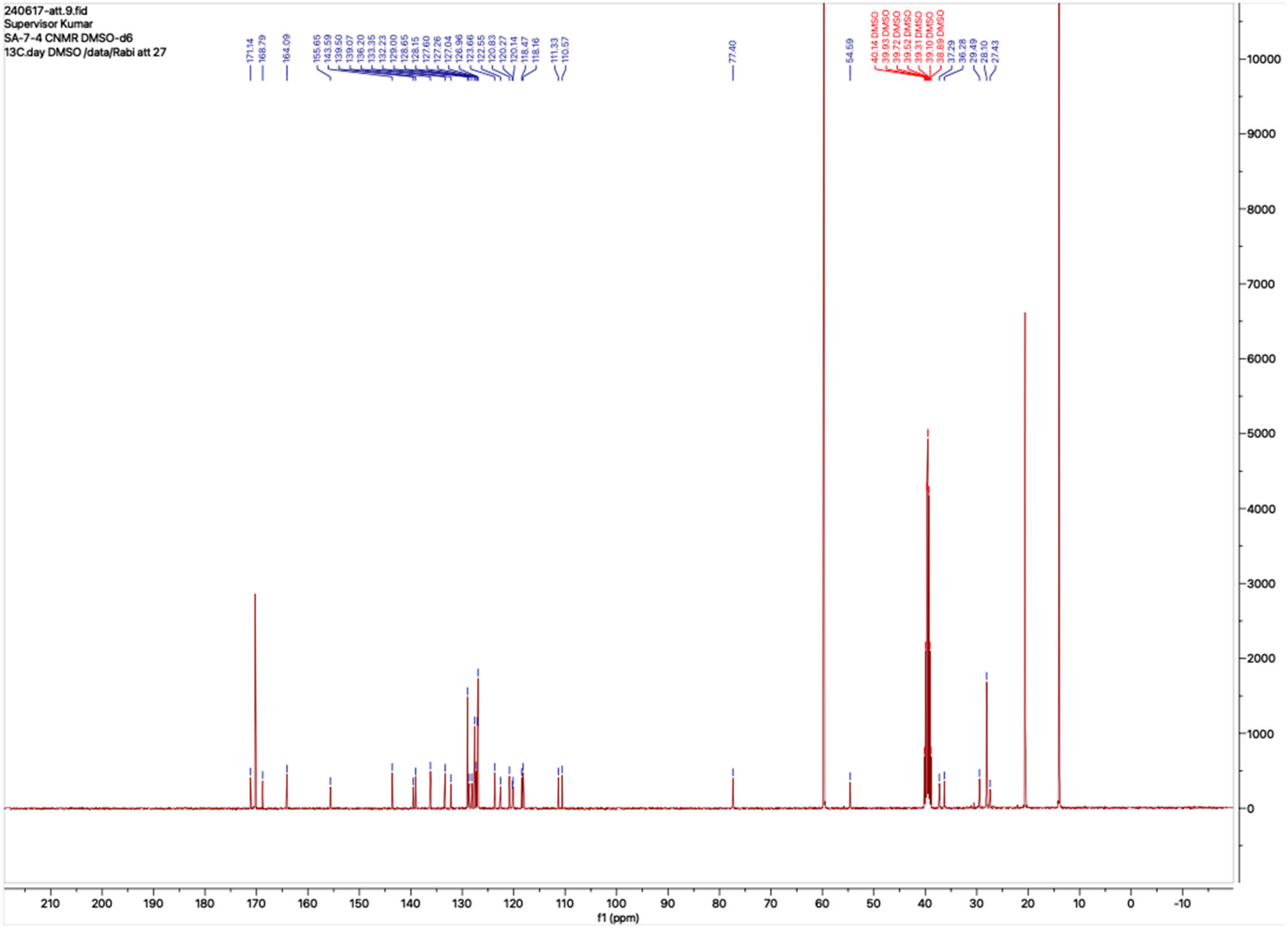

$^{1}$H NMR of (*S*)-3-(2-(2-([1,1'-biphenyl]-2-carboxamido)benzamido)-3-phenylpropanamido)propan-1-aminium (2)

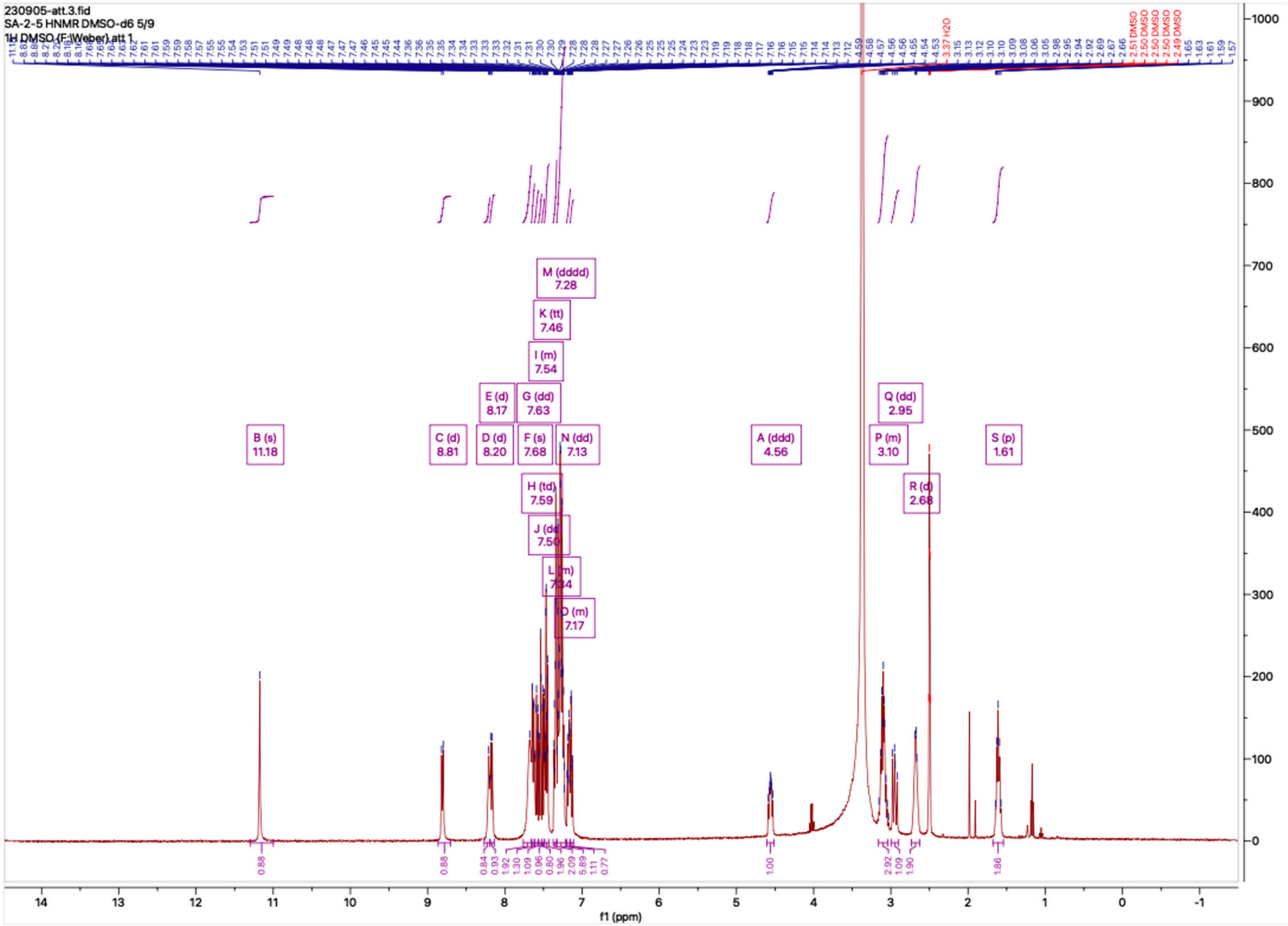

$^{13}$C NMR of (*S*)-3-(2-(2-([1,1'-biphenyl]-2-carboxamido)benzamido)-3-phenylpropanamido)propan-1-aminium (2)

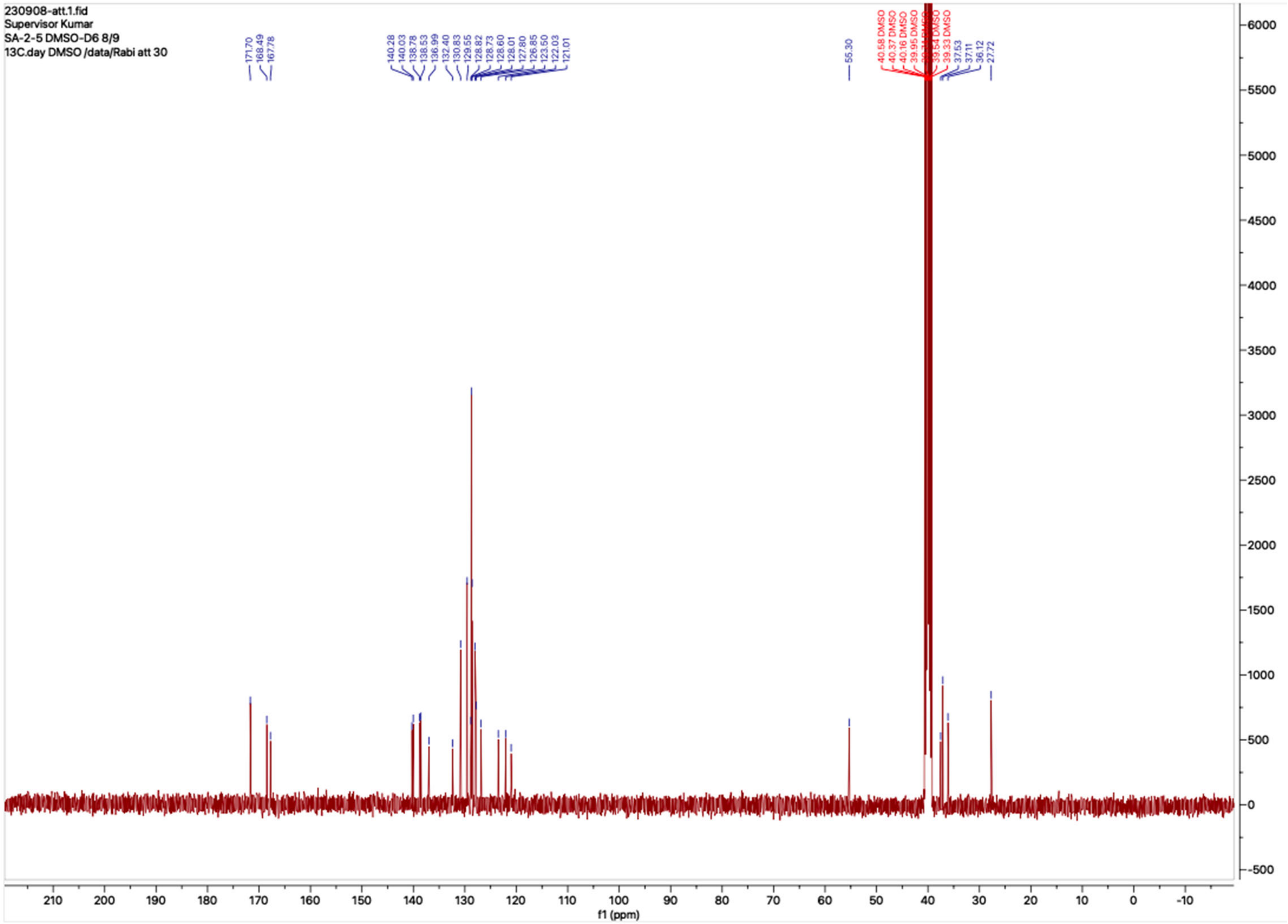

$^{1}$H NMR of (*S*)-3-(2-(2-([1,1'-biphenyl]-3-carboxamido)benzamido)-3-phenylpropanamido)propan-1-aminium (3)

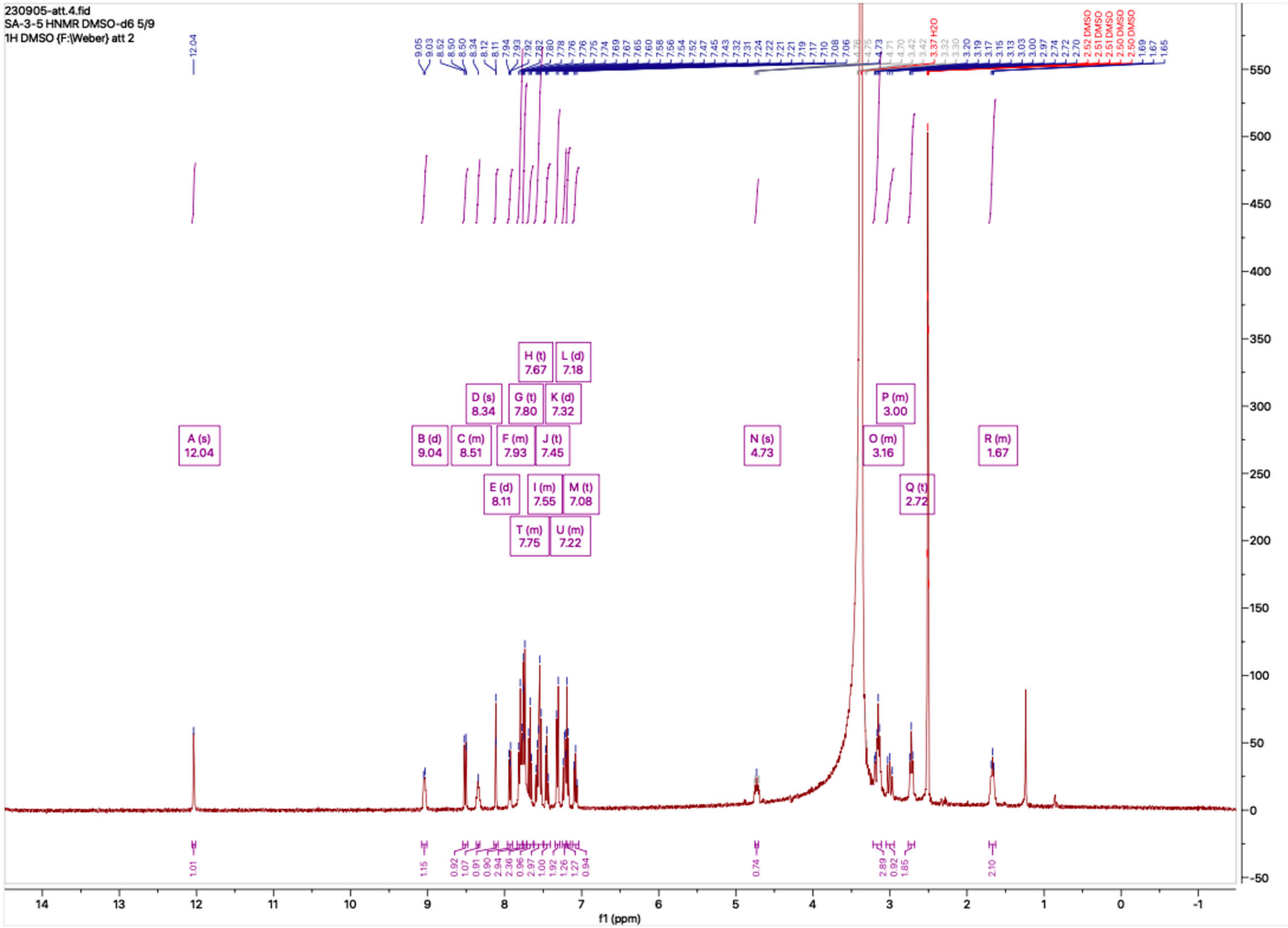

$^{13}$C NMR of (*S*)-3-(2-(2-([1,1'-biphenyl]-3-carboxamido)benzamido)-3-phenylpropanamido)propan-1-aminium (3)

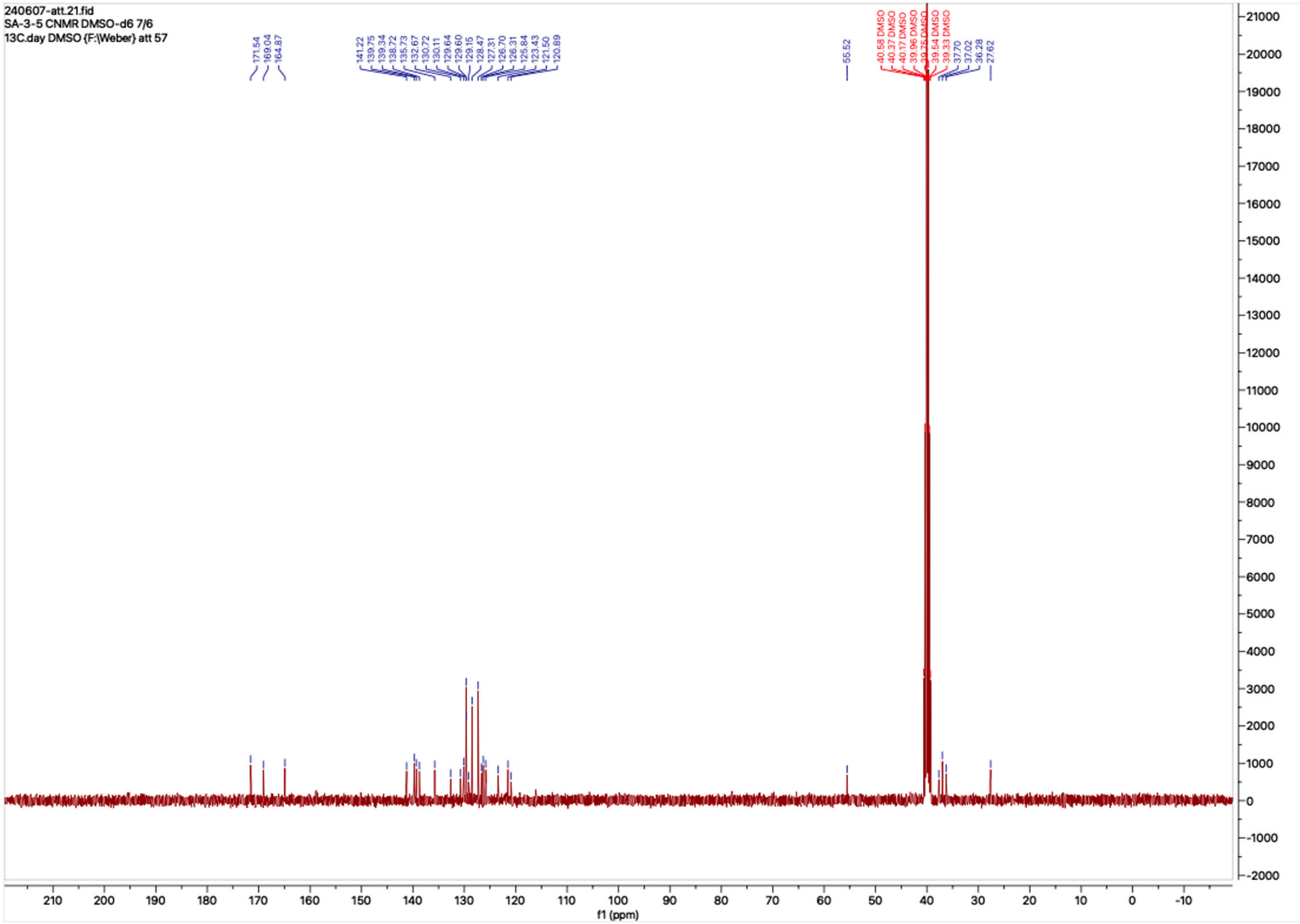

$^{1}$H NMR of (*S*)-3-(2-(2-([1,1'-biphenyl]-4-carboxamido)benzamido)-3-phenylpropanamido)propan-1-aminium (4)

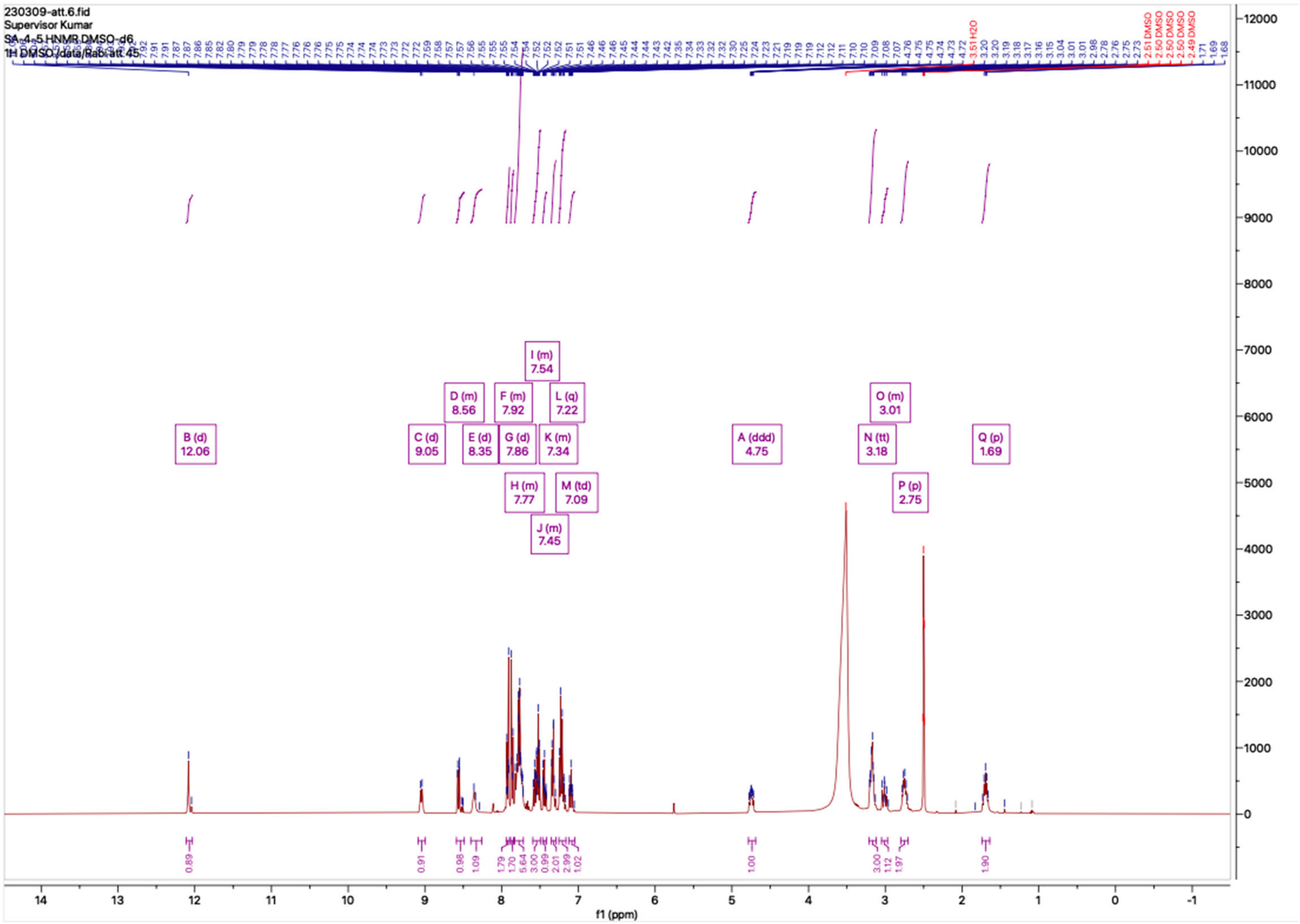

$^{13}$C NMR of (*S*)-3-(2-(2-([1,1'-biphenyl]-4-carboxamido)benzamido)-3-phenylpropanamido)propan-1-aminium (4)

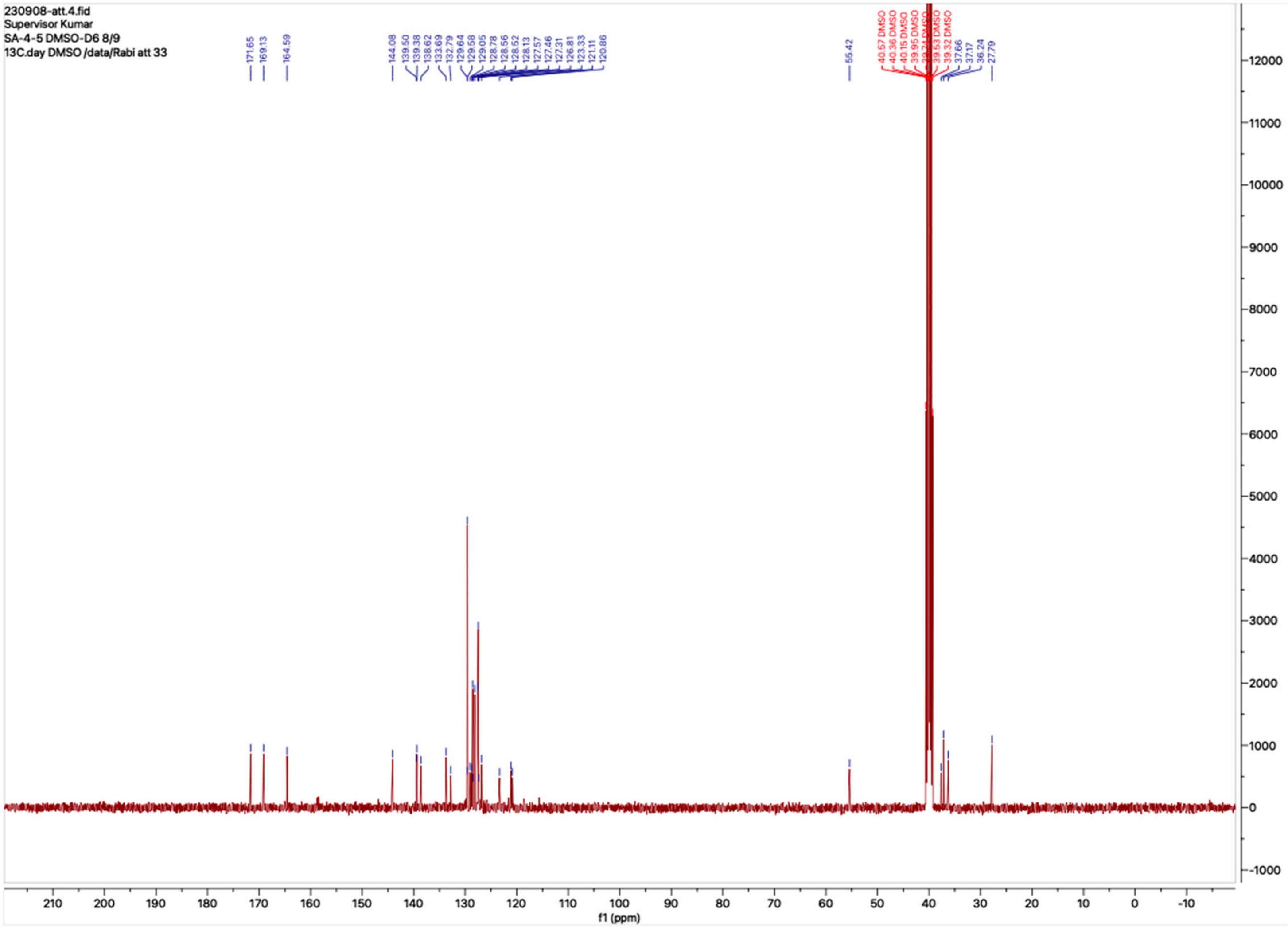

$^1$H NMR of (*S*)-3-(2-(2-(2-naphthamido)benzamido)-3-(1*H*-indol-3-yl)propanamido)propan-1-aminium (6)

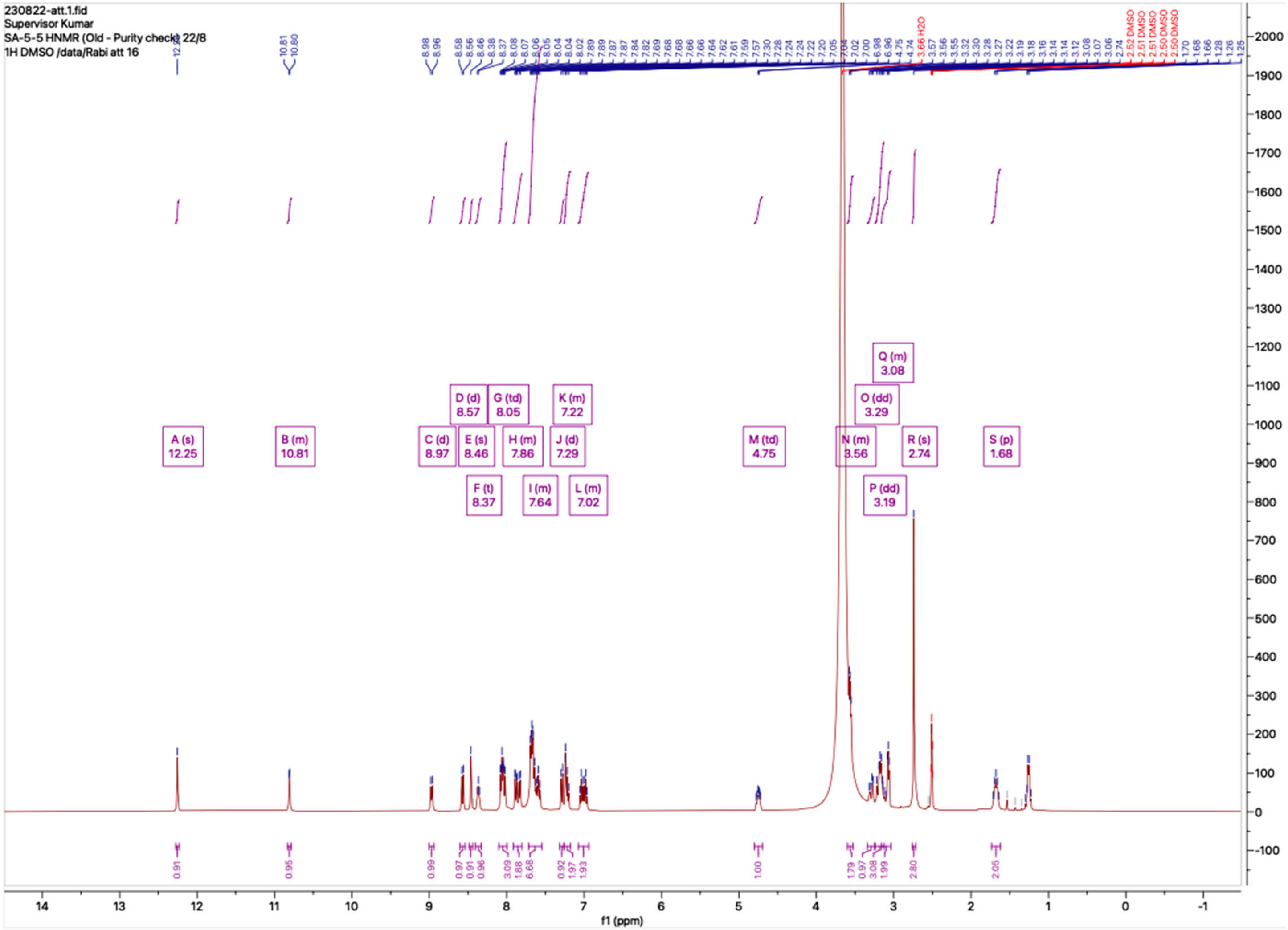

$^{13}$C NMR of (*S*)-3-(2-(2-(2-naphthamido)benzamido)-3-(1*H*-indol-3-yl)propanamido)propan-1-aminium (6)

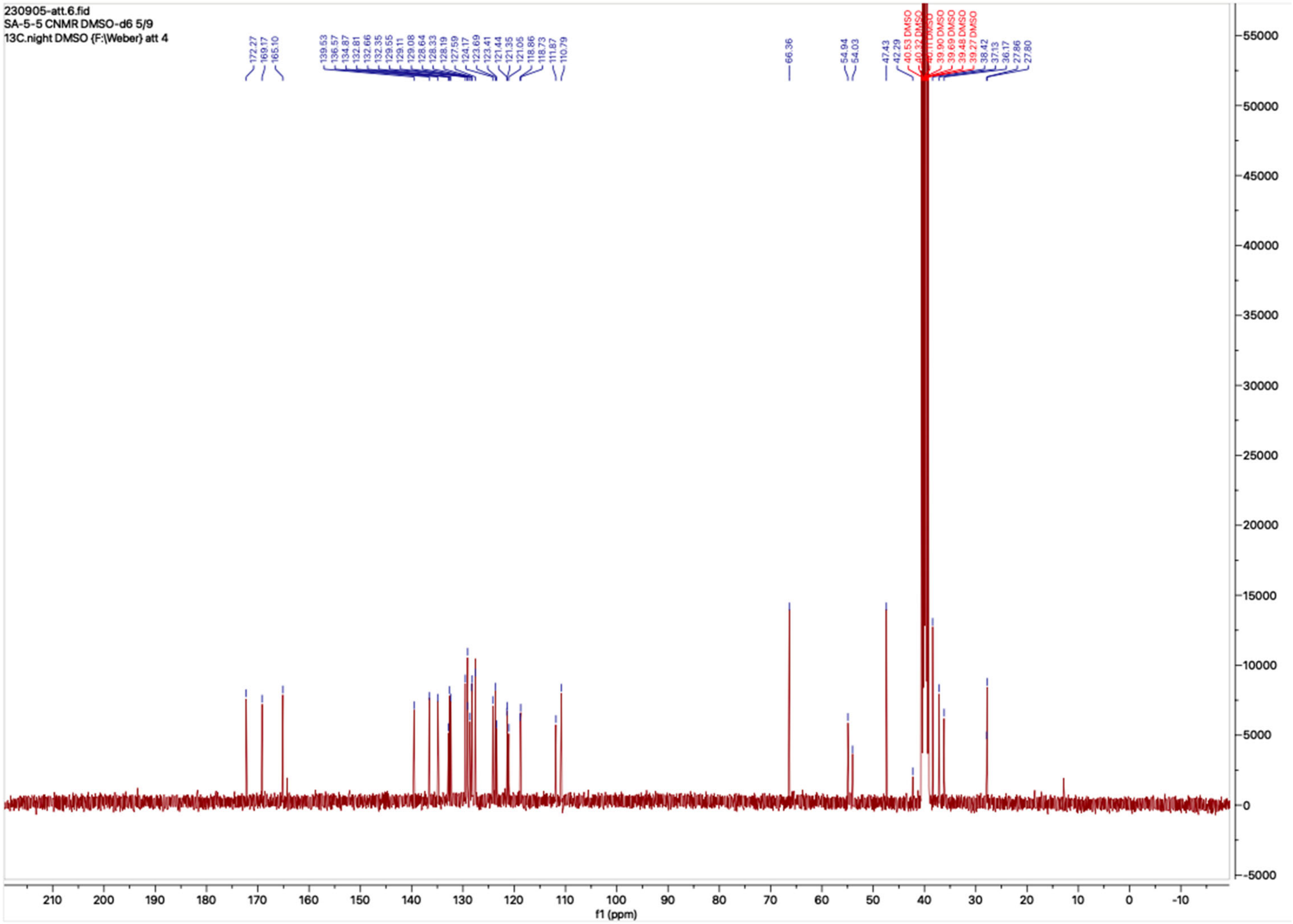

$^{1}$H NMR of (*S*)-3-(2-(2-([1,1'-biphenyl]-4-carboxamido)benzamido)-3-(1*H*-indol-3-yl)propanamido)propan-1-aminium (7)

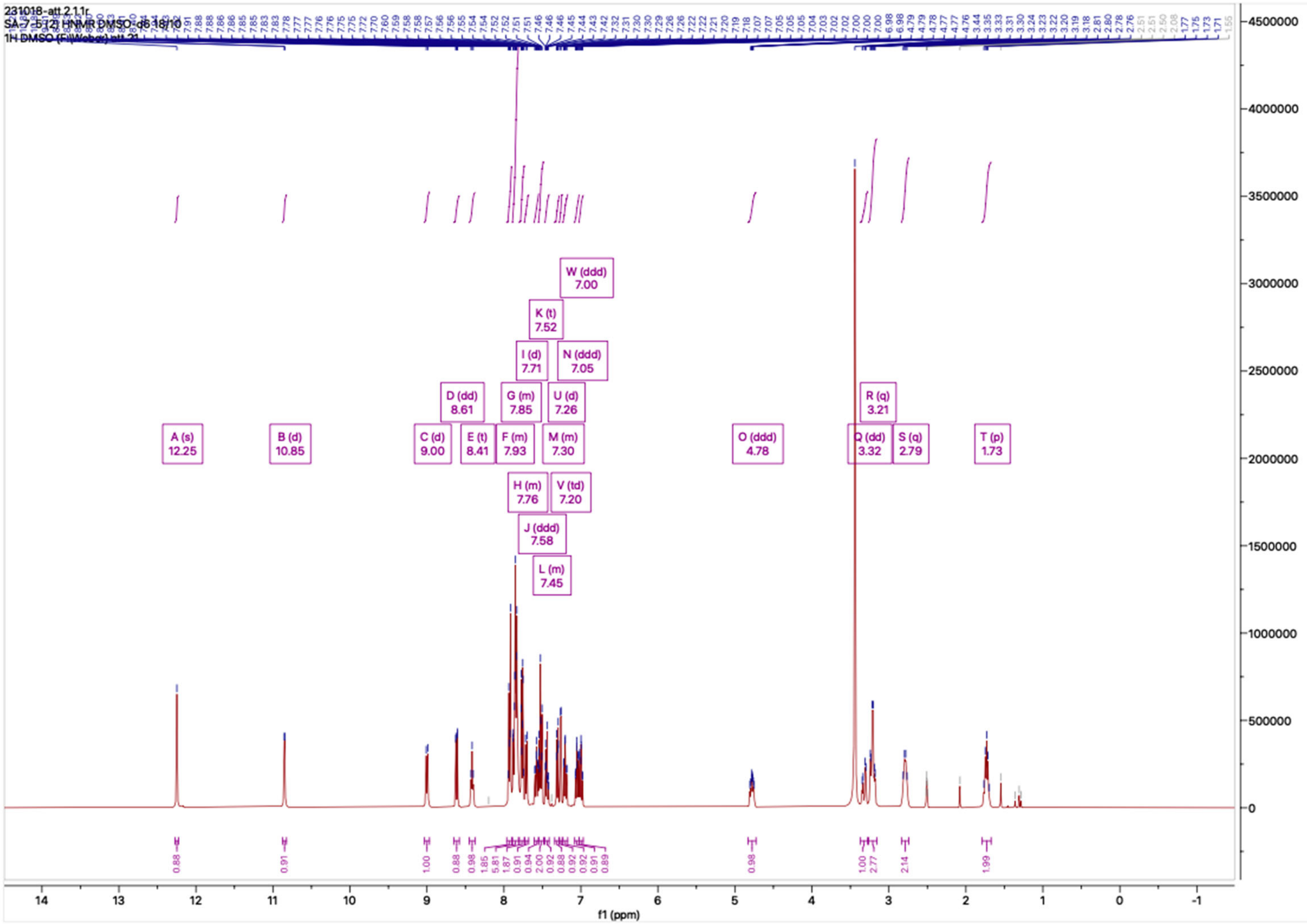

$^{13}$C NMR of (*S*)-3-(2-(2-([1,1'-biphenyl]-4-carboxamido)benzamido)-3-(1*H*-indol-3-yl)propanamido)propan-1-aminium (7)

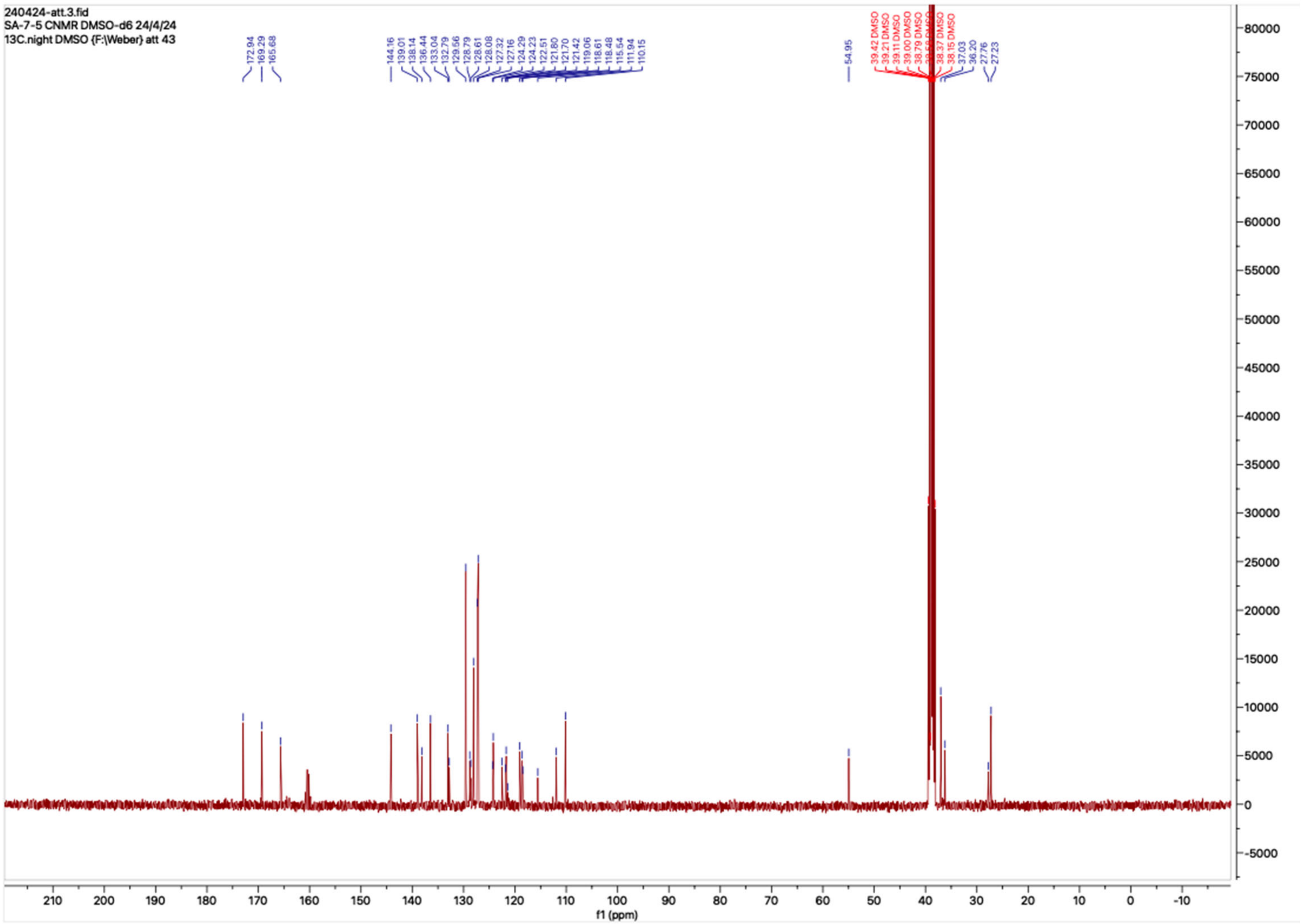

$^{1}$H NMR of (*S*)-3-(2-(2-(2-naphthamido)-5-bromobenzamido)-3-phenylpropanamido)propan-1-aminium (8)

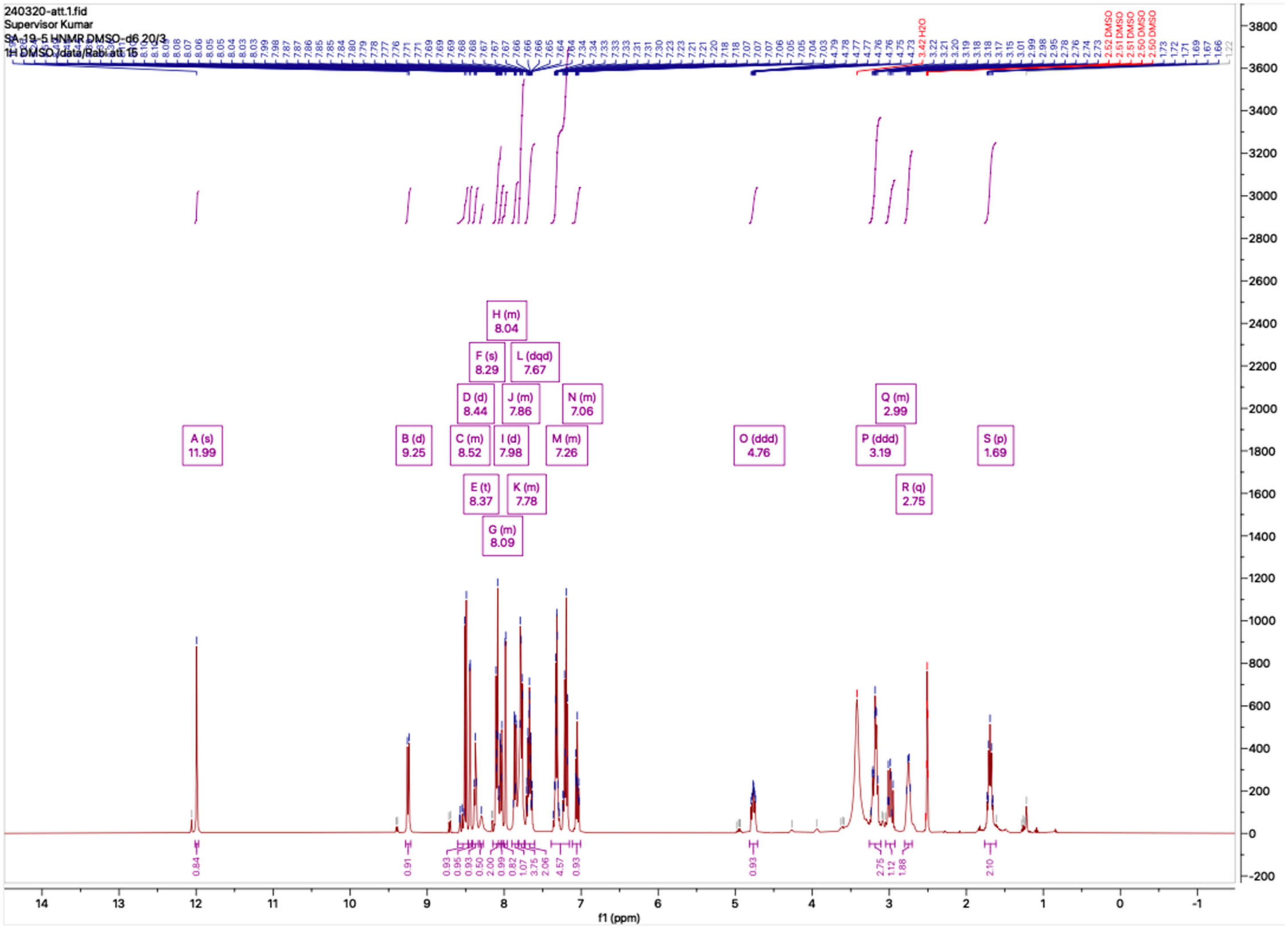

$^{13}$C NMR of (*S*)-3-(2-(2-(2-naphthamido)-5-bromobenzamido)-3-phenylpropanamido)propan-1-aminium (8)

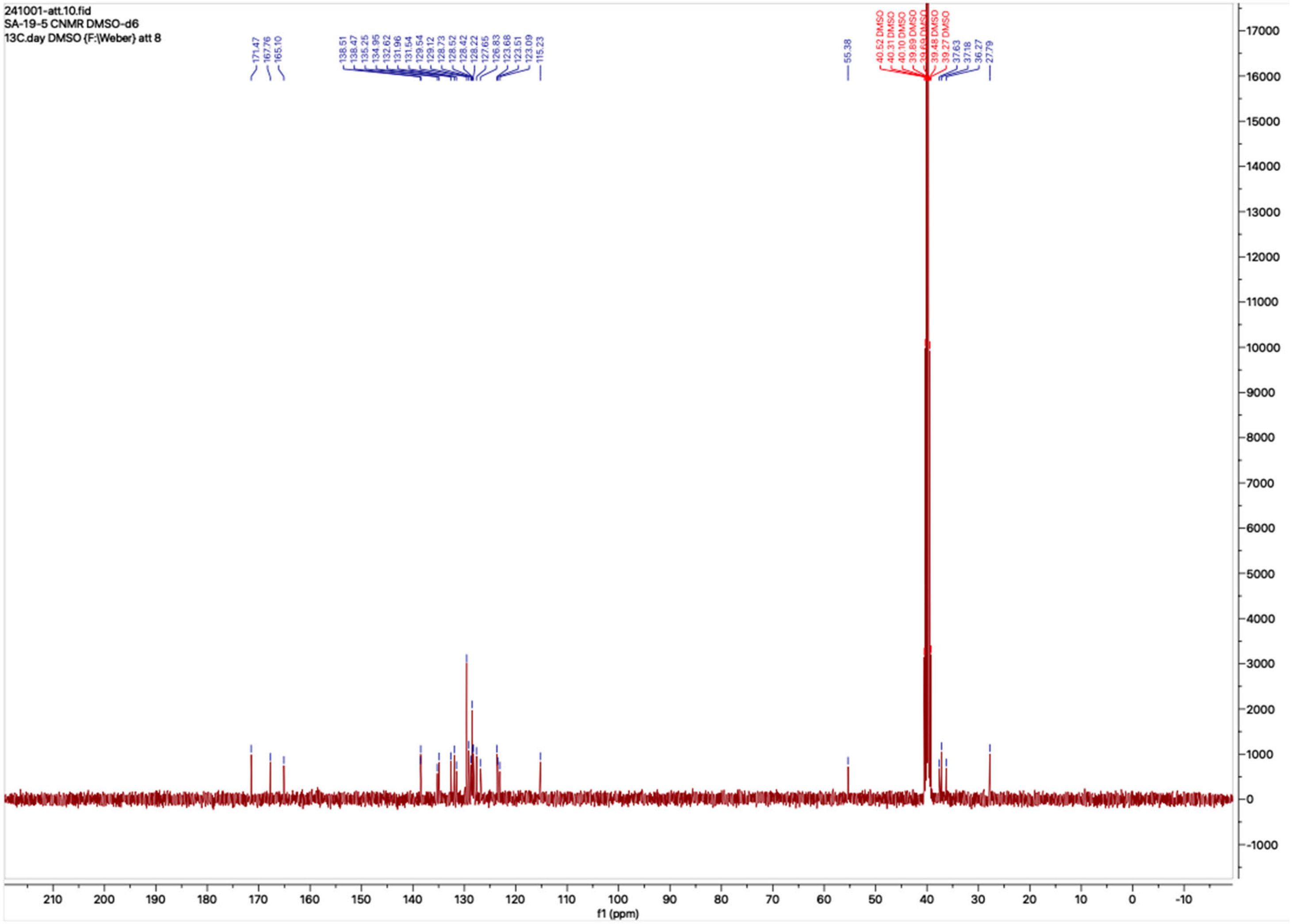

$^{1}$H NMR of (*S*)-3-(2-(2-(2-naphthamido)-5-bromobenzamido)-3-(1*H*-indol-3-yl)propanamido)propan-1-aminium (9)

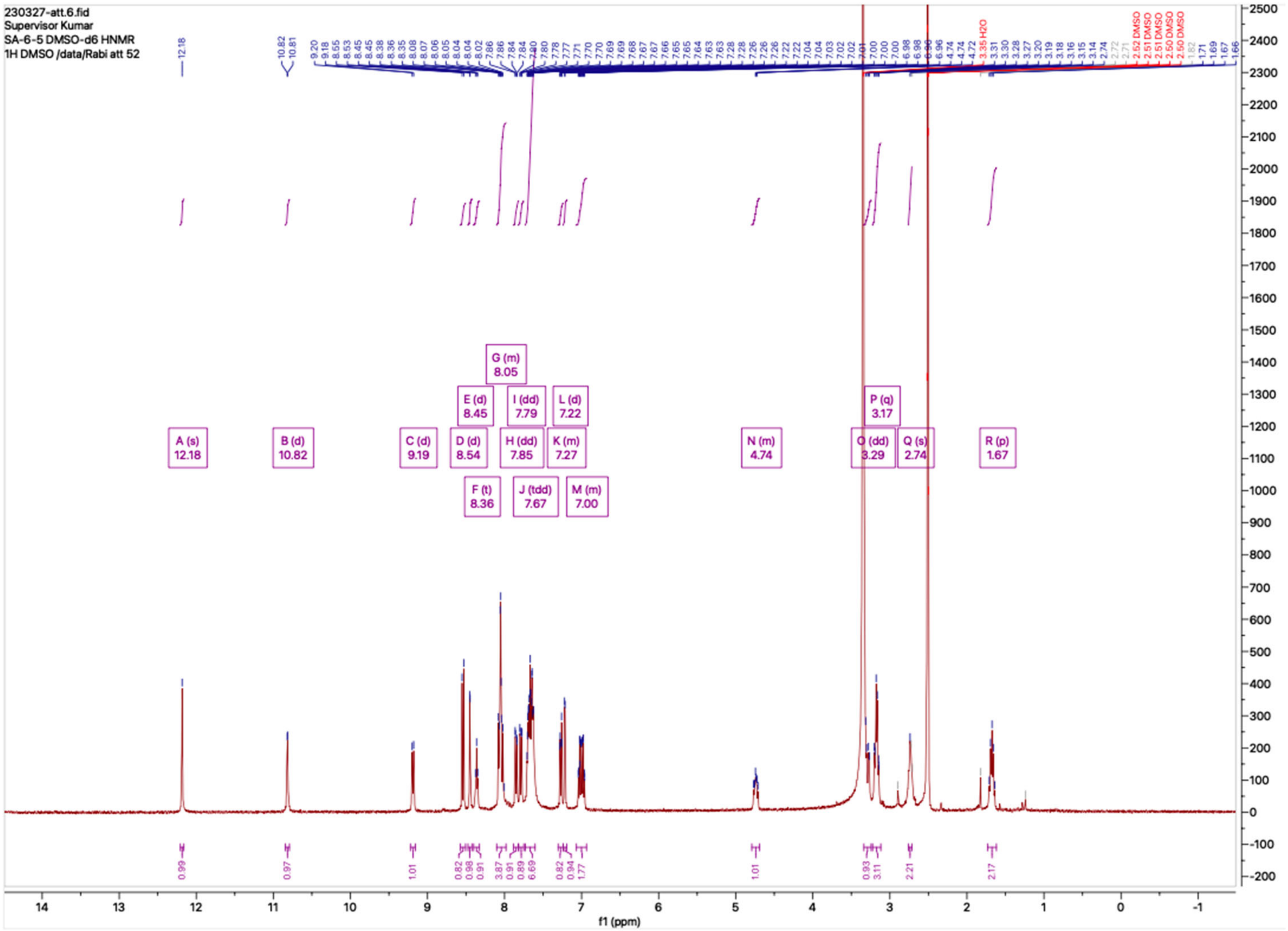

$^{13}$C NMR of (*S*)-3-(2-(2-(2-naphthamido)-5-bromobenzamido)-3-(1*H*-indol-3-yl)propanamido)propan-1-aminium (9)

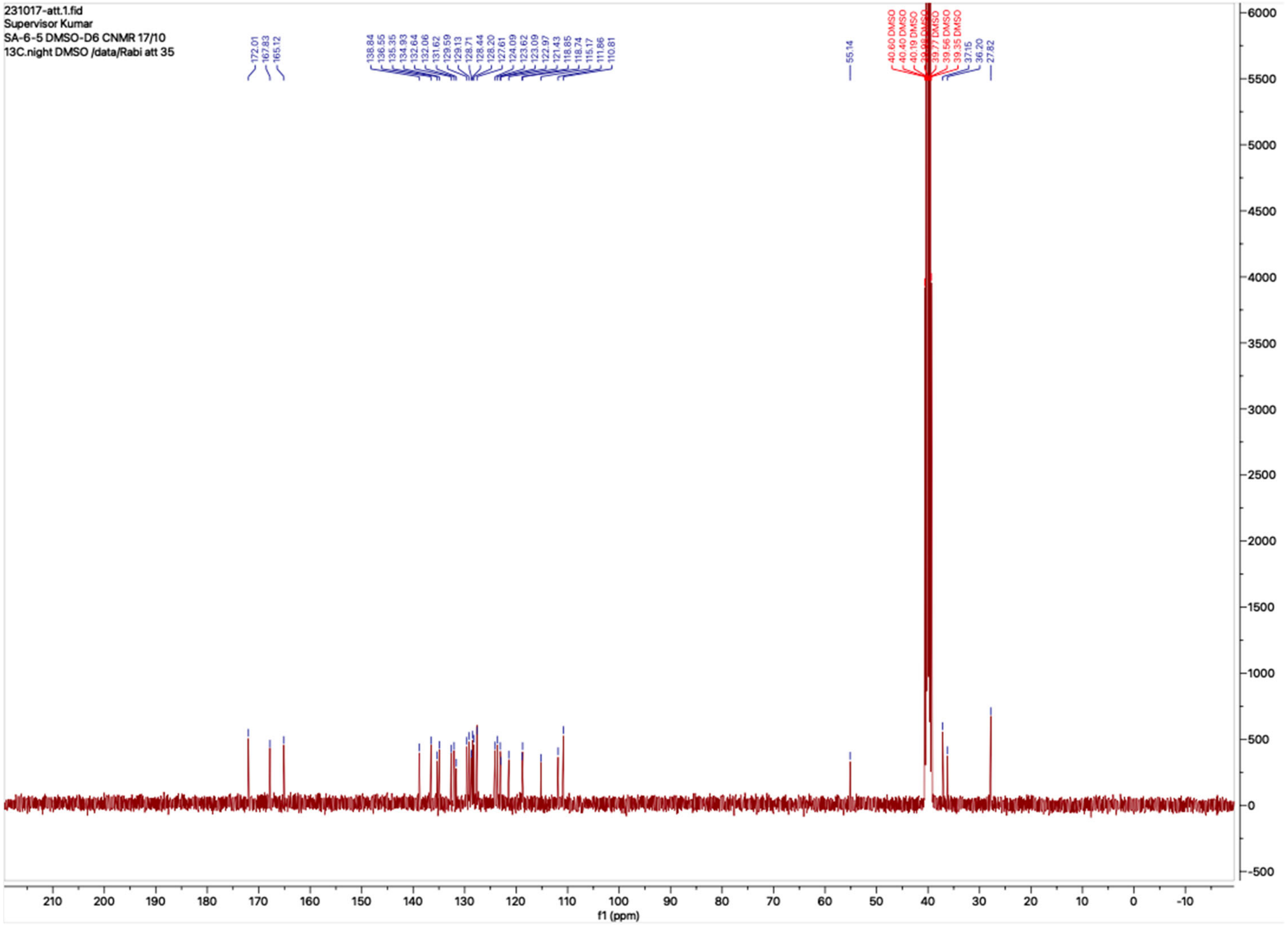

$^{1}$H NMR of *tert*-butyl (*S*)-(3-(2-((((9*H*-fluoren-9-yl)methoxy)carbonyl)amino)-3-phenylpropanamido)propyl)carbamate (20)

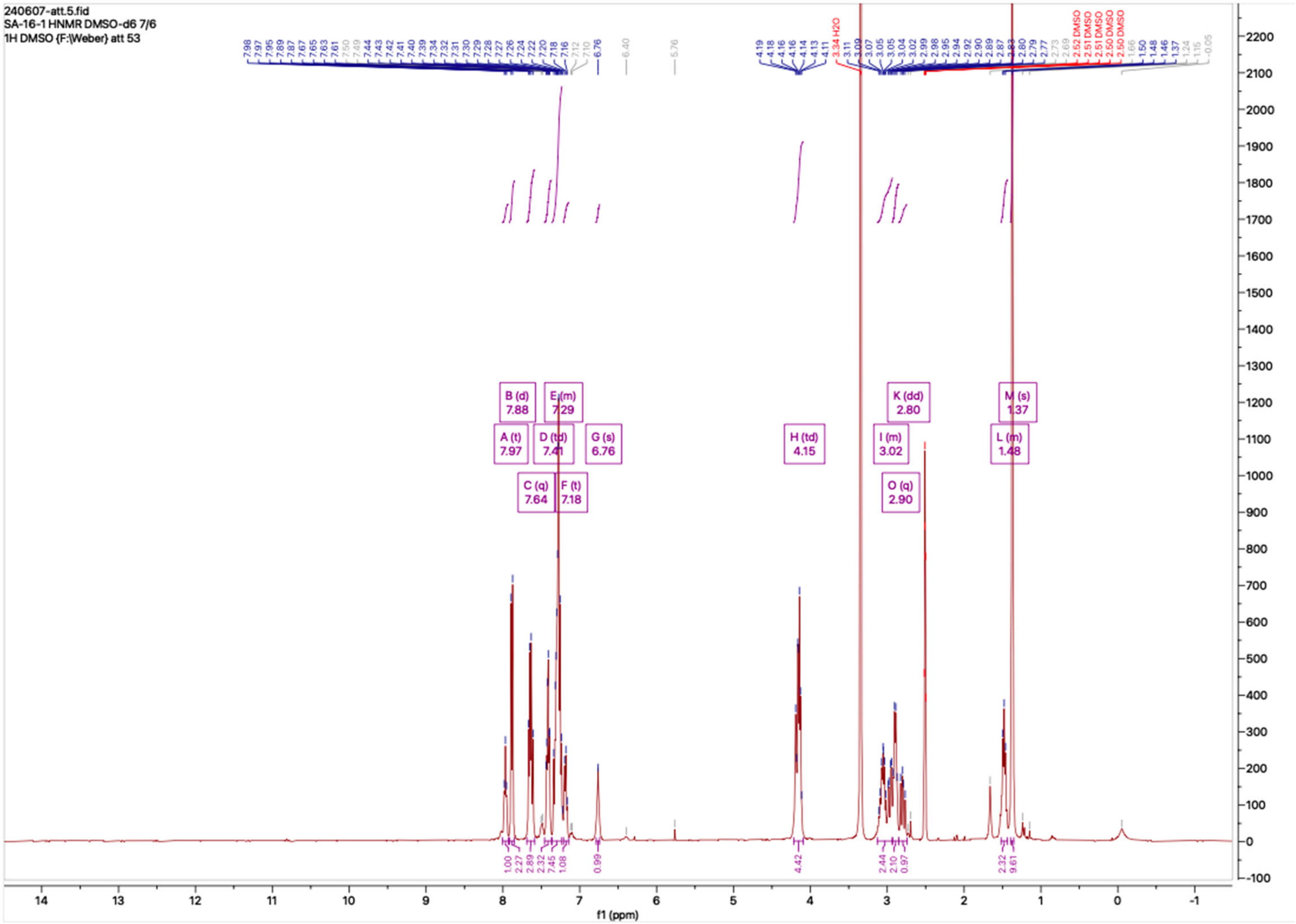

$^{13}$C NMR of *tert*-butyl (*S*)-(3-(2-((((9*H*-fluoren-9-yl)methoxy)carbonyl)amino)-3-phenylpropanamido)propyl)carbamate (20)

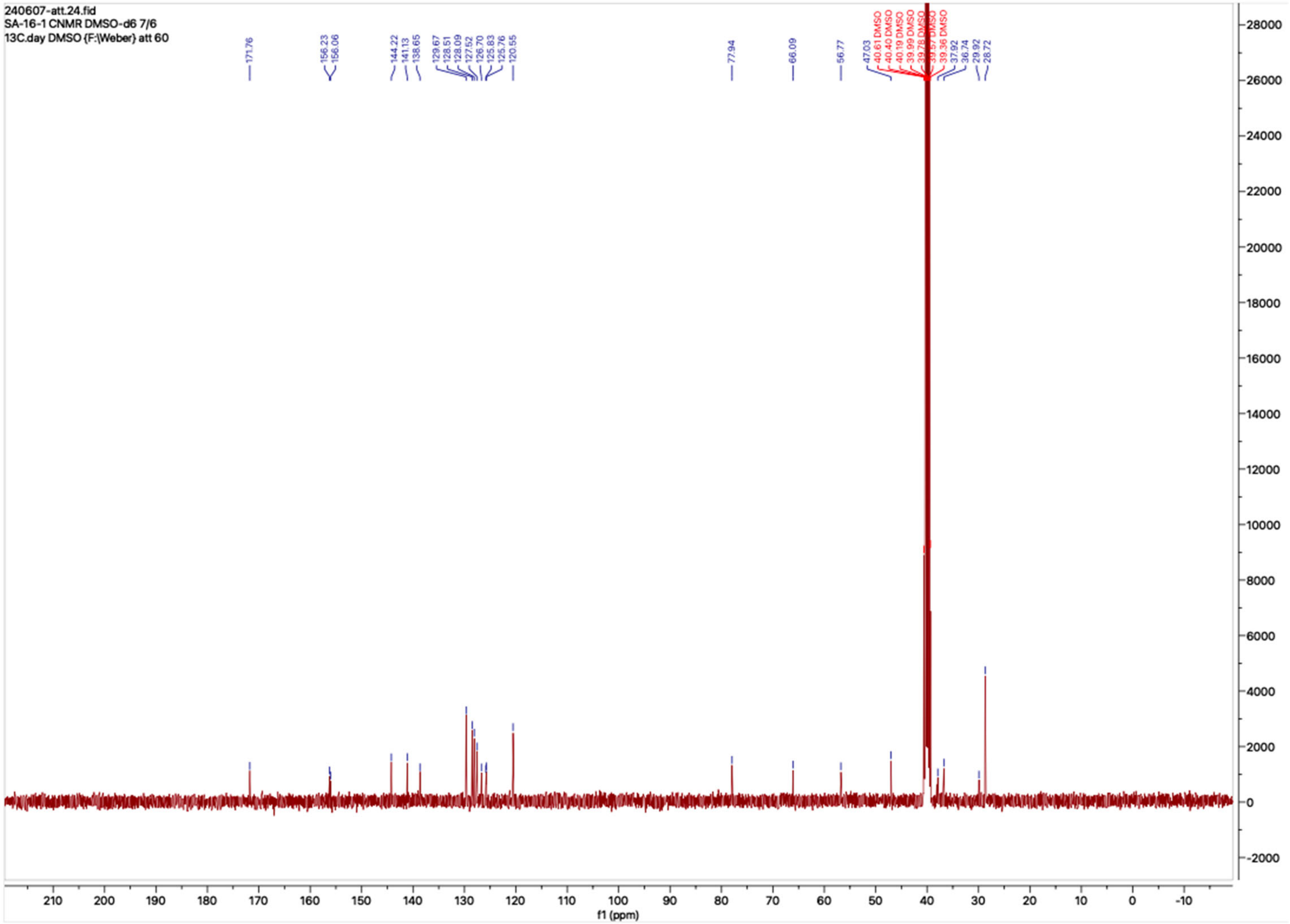

$^{1}$H NMR of *tert*-butyl (*S*)-(3-(2-amino-3-phenylpropanamido)propyl)carbamate (21)

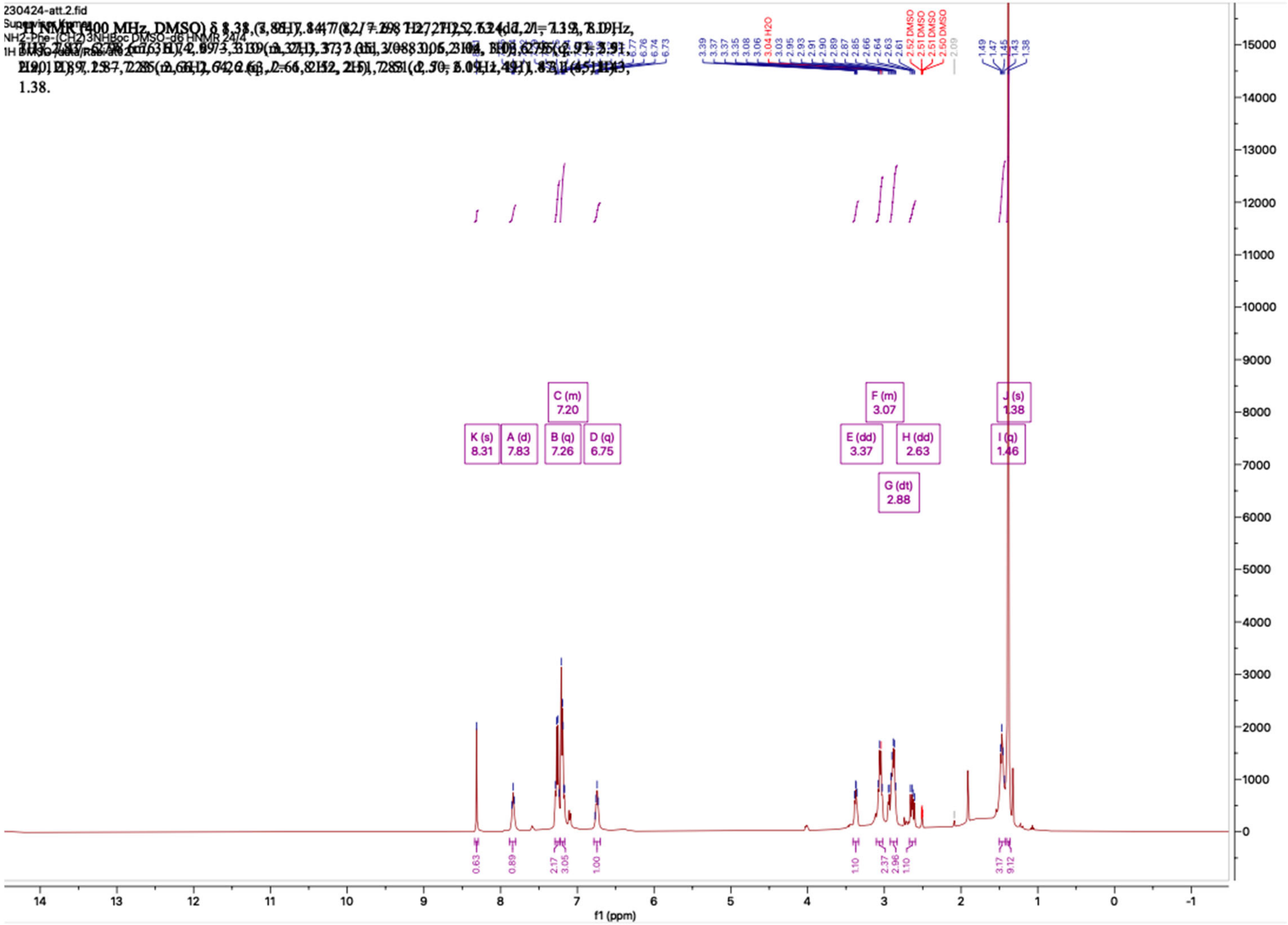

$^{13}$C NMR of *tert*-butyl (*S*)-(3-(2-amino-3-phenylpropanamido)propyl)carbamate (21)

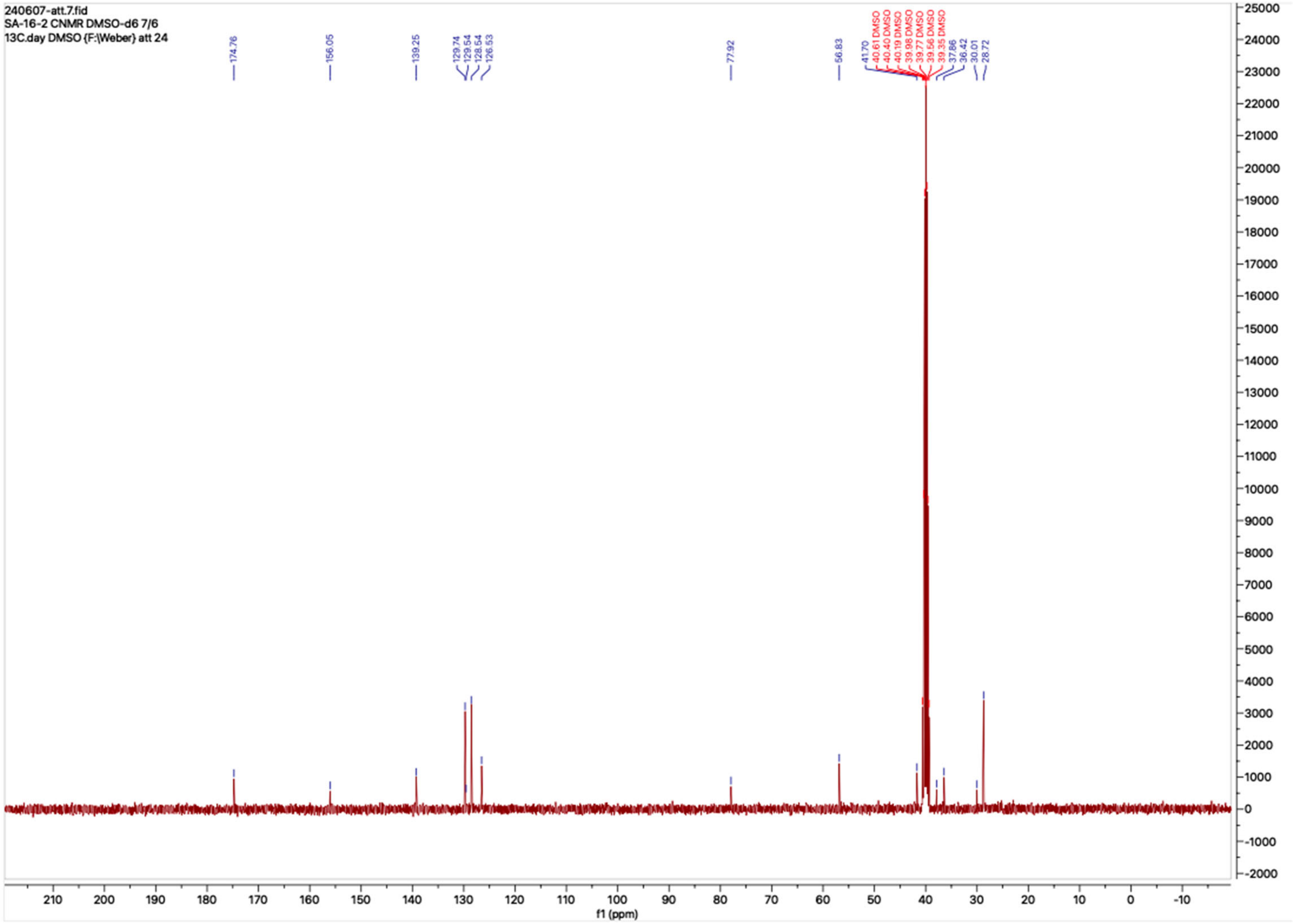

$^{1}$H NMR of *tert*-butyl (*S*)-(3-(2-(2-aminobenzamido)-3-phenylpropanamido)propyl)carbamate (22a)

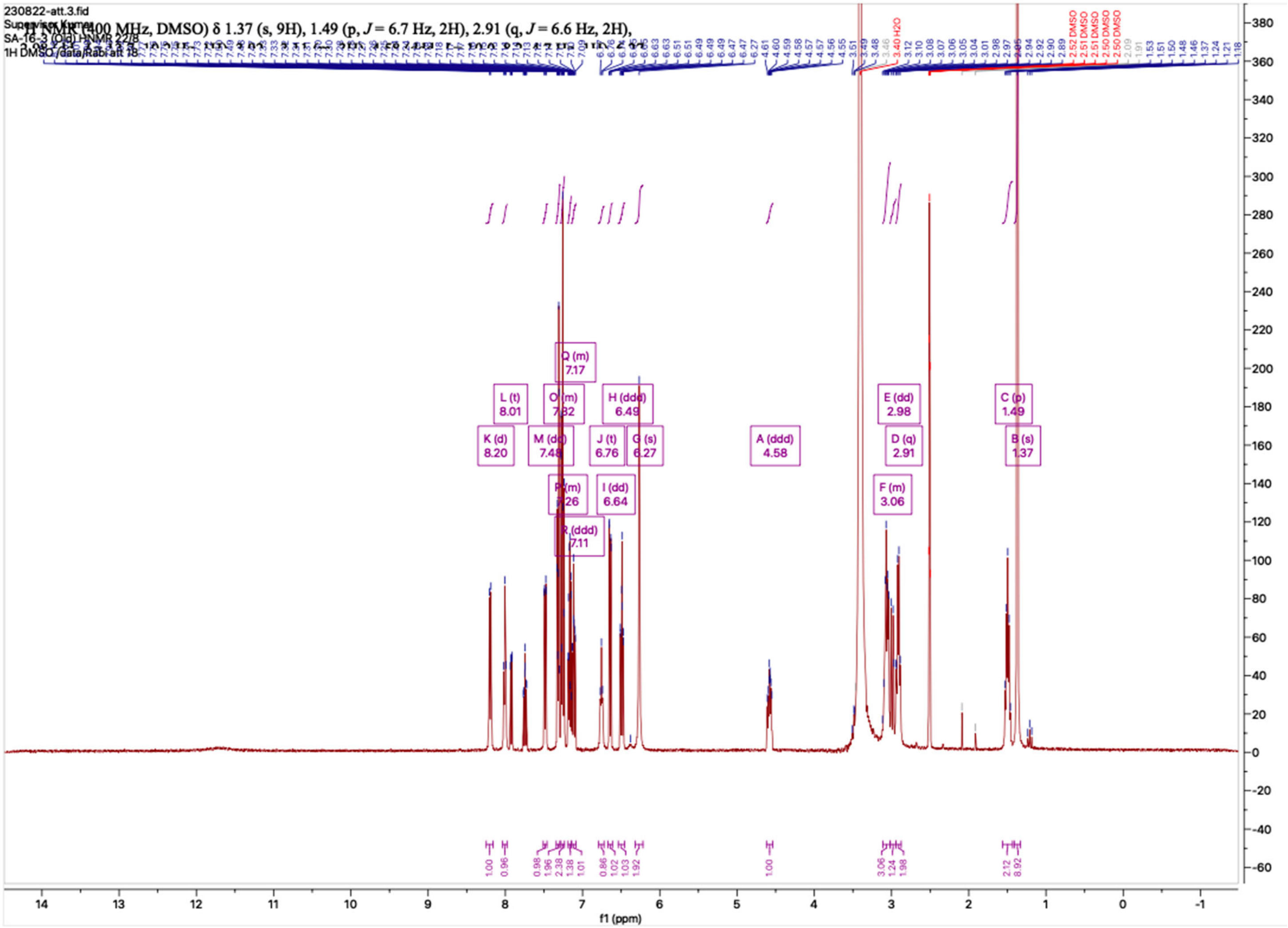

$^{13}$C NMR of *tert*-butyl (*S*)-(3-(2-(2-aminobenzamido)-3-phenylpropanamido)propyl)carbamate (22a)

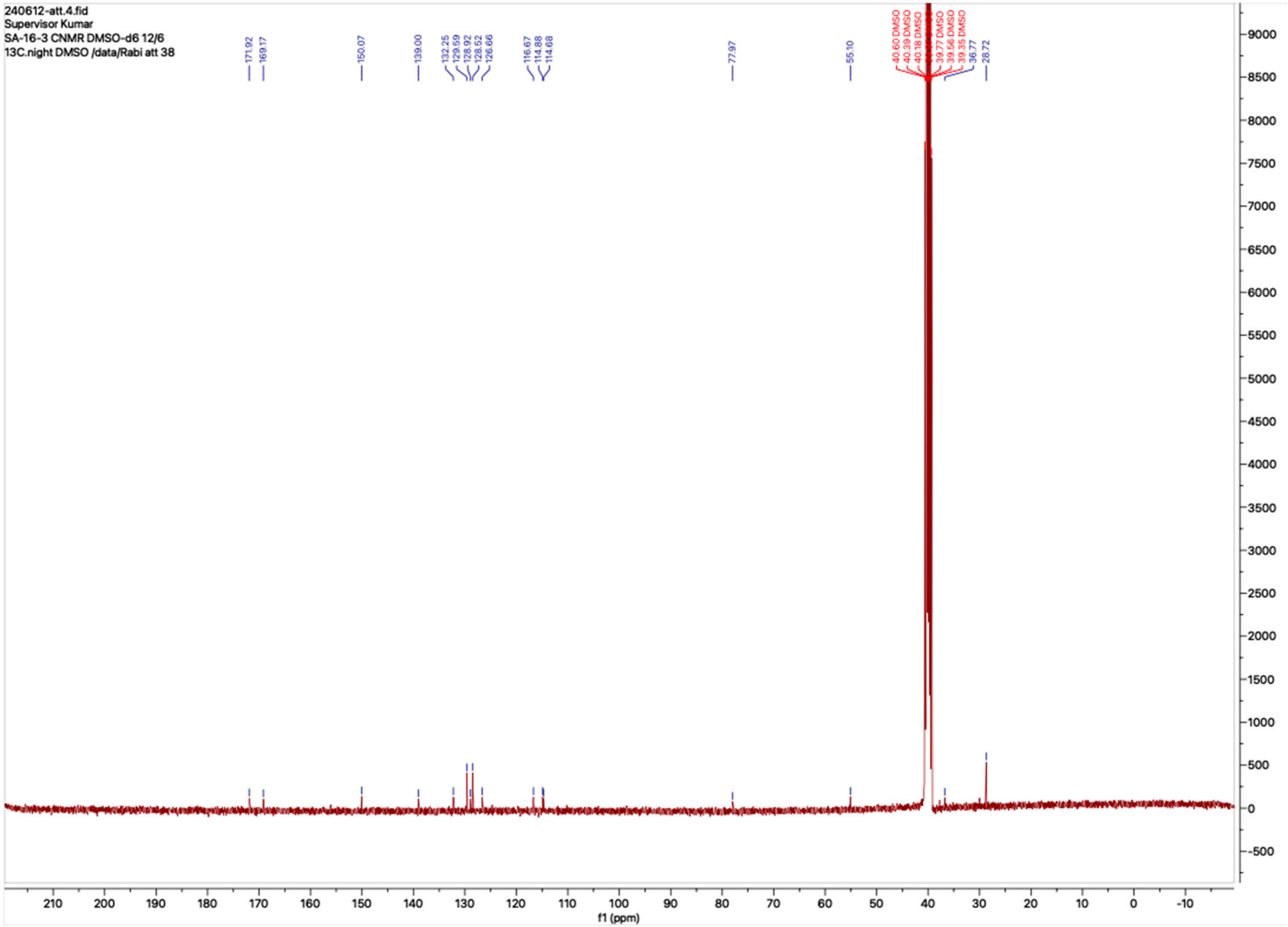

$^{1}$H NMR of *tert*-butyl (*S*)-(3-(2-(2-amino-5-bromobenzamido)-3-phenylpropanamido)propyl)carbamate (22b)

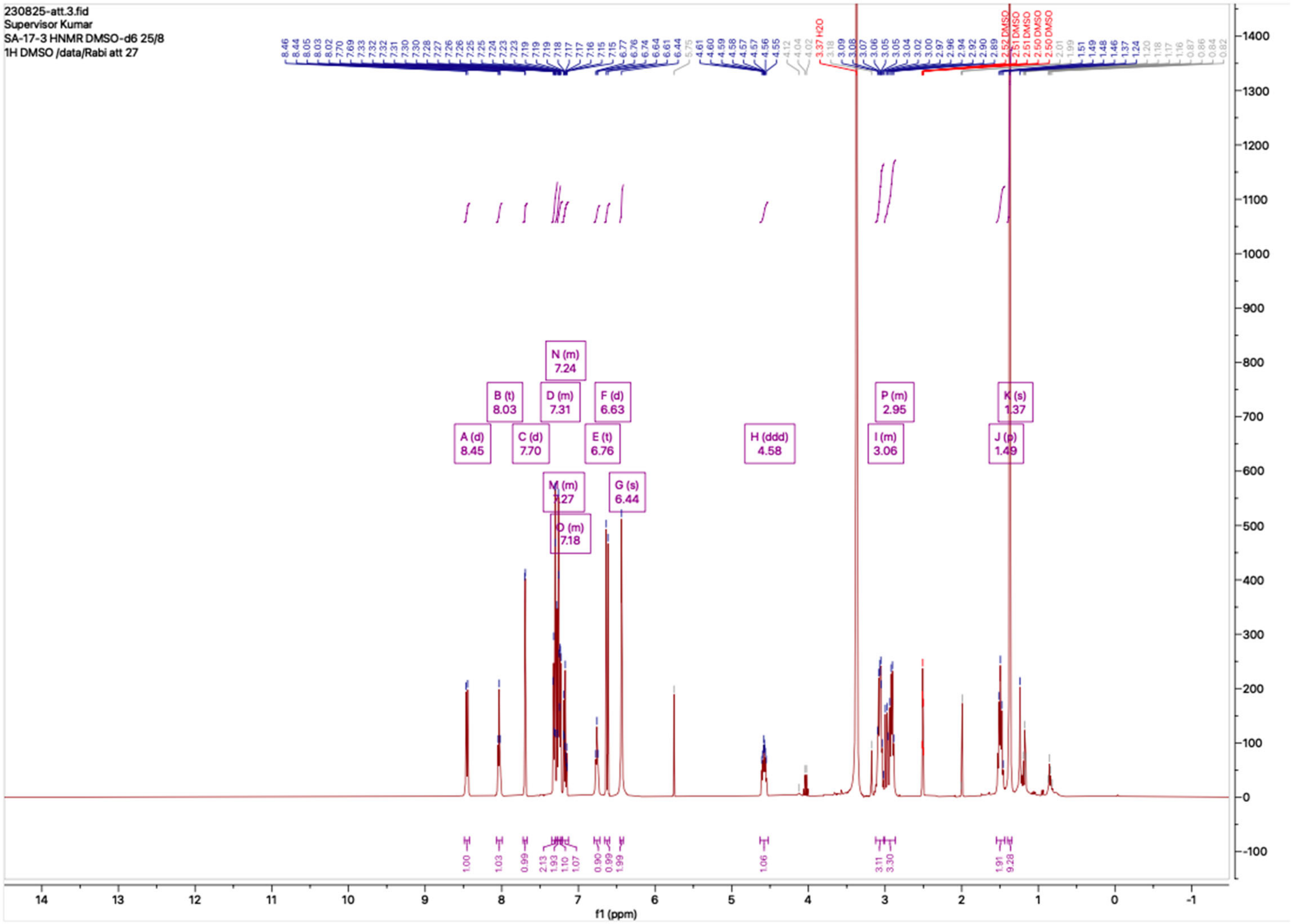

$^{13}$C NMR of *tert*-butyl (*S*)-(3-(2-(2-amino-5-bromobenzamido)-3-phenylpropanamido)propyl)carbamate (22b)

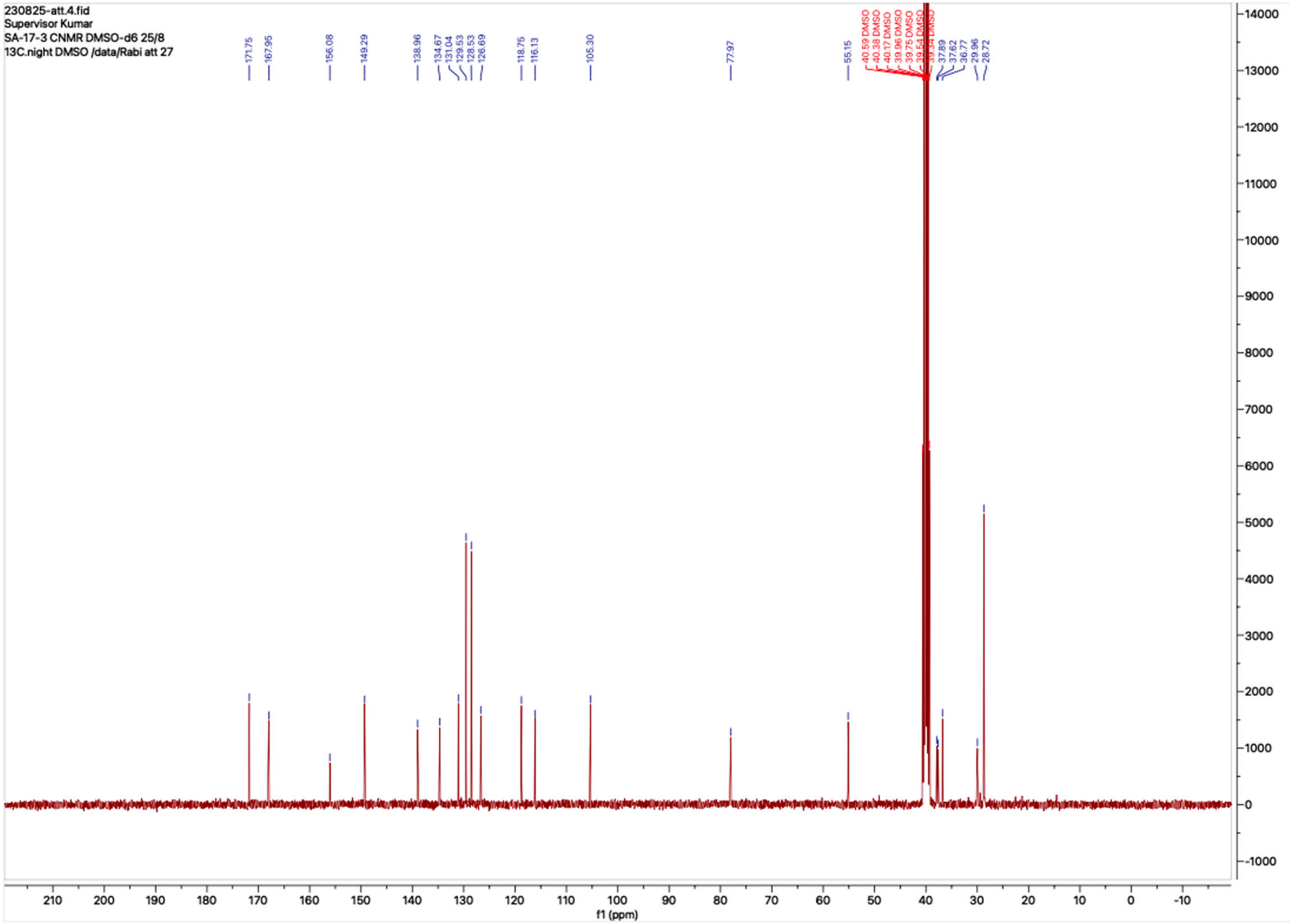

$^{1}$H NMR of (*S*)-2-((1-((3-ammoniopropyl)amino)-1-oxo-3-phenylpropan-2-yl)carbamoyl)benzenaminium (5)

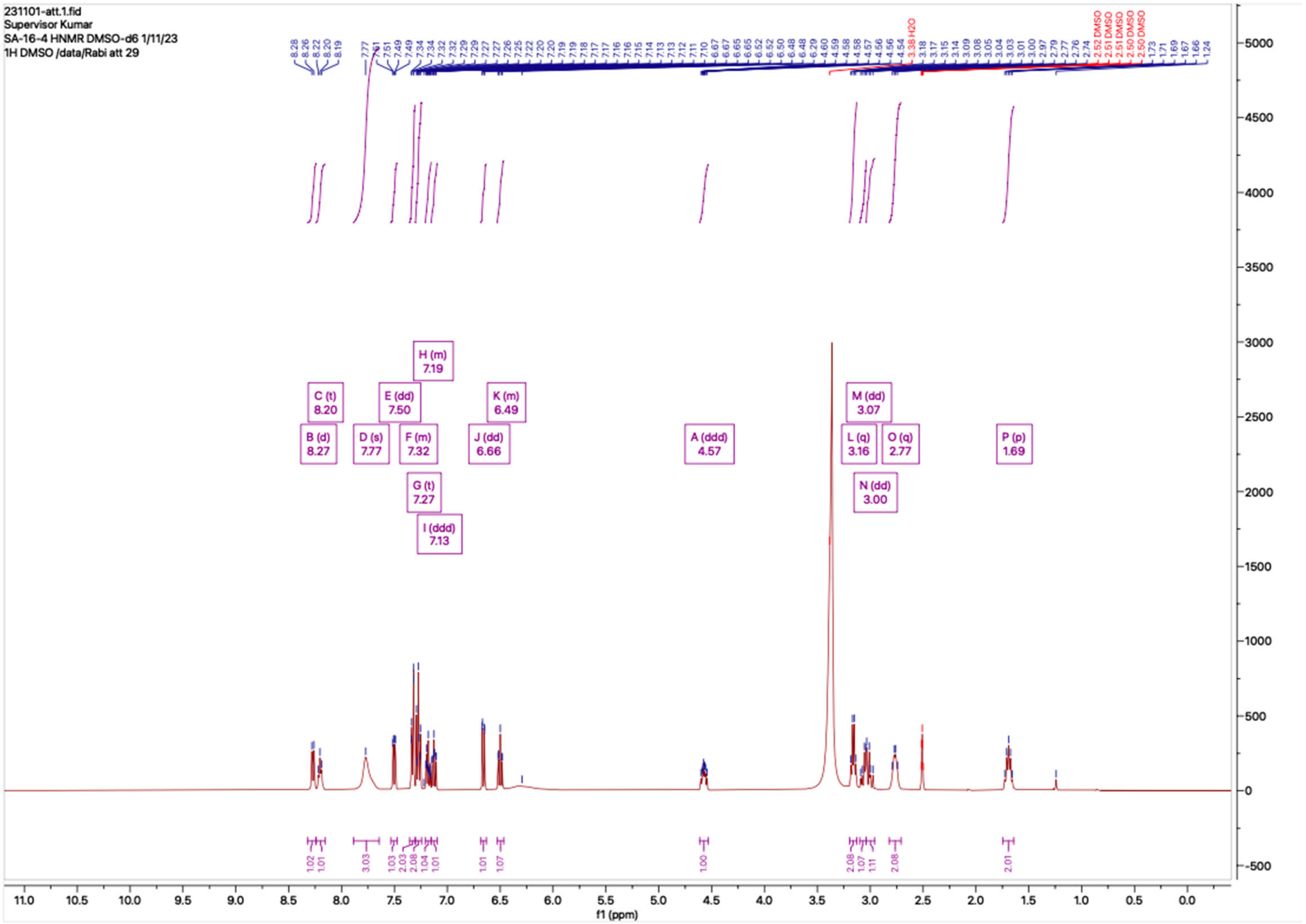

$^{13}C$ NMR of (*S*)-2-((1-((3-ammoniopropyl)amino)-1-oxo-3-phenylpropan-2-yl)carbamoyl)benzenaminium (5)

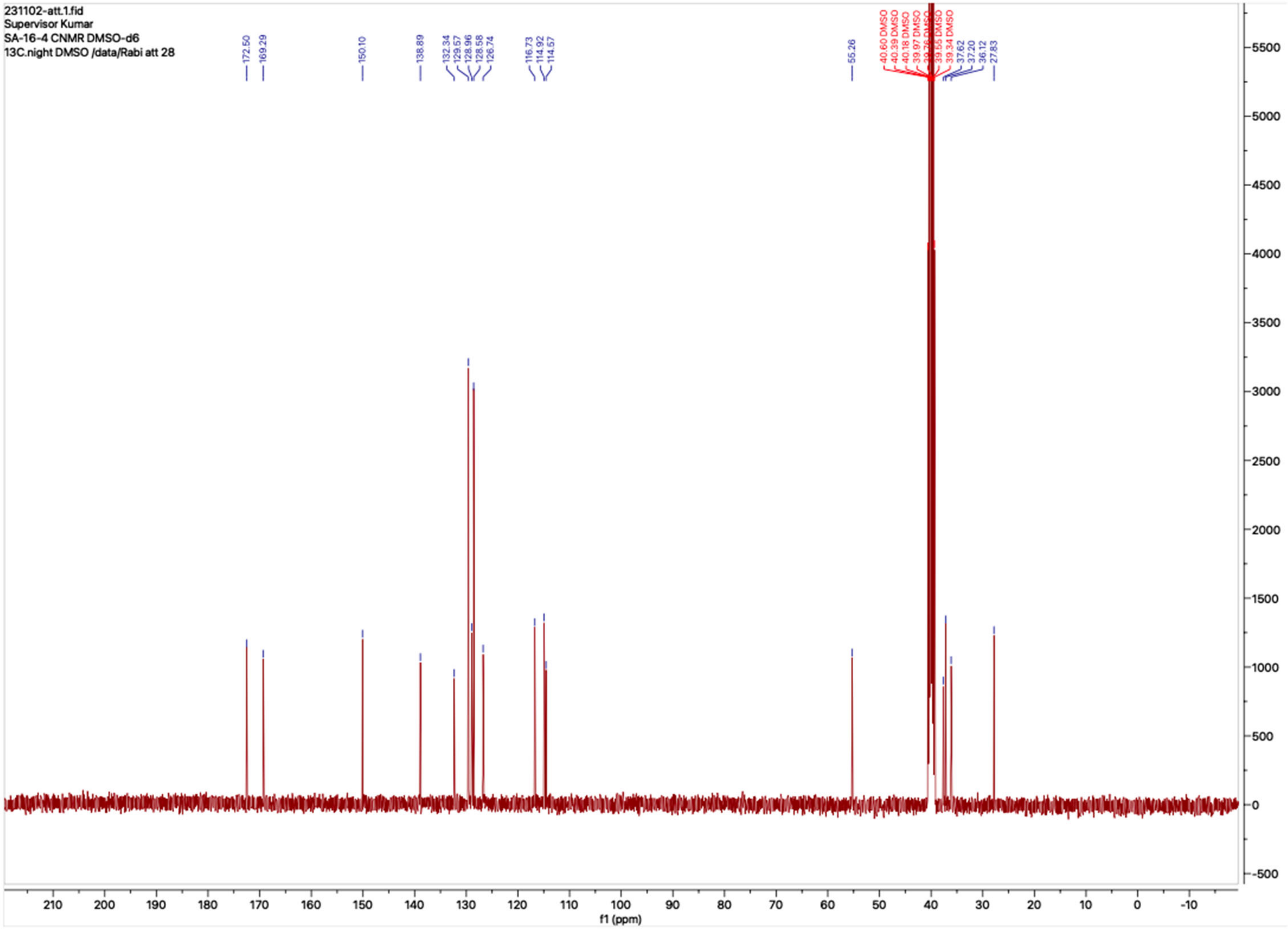

$^{1}$H NMR of (*S*)-2-((1-((3-ammoniopropyl)amino)-1-oxo-3-phenylpropan-2-yl)carbamoyl)-4-bromobenzenaminium (10)

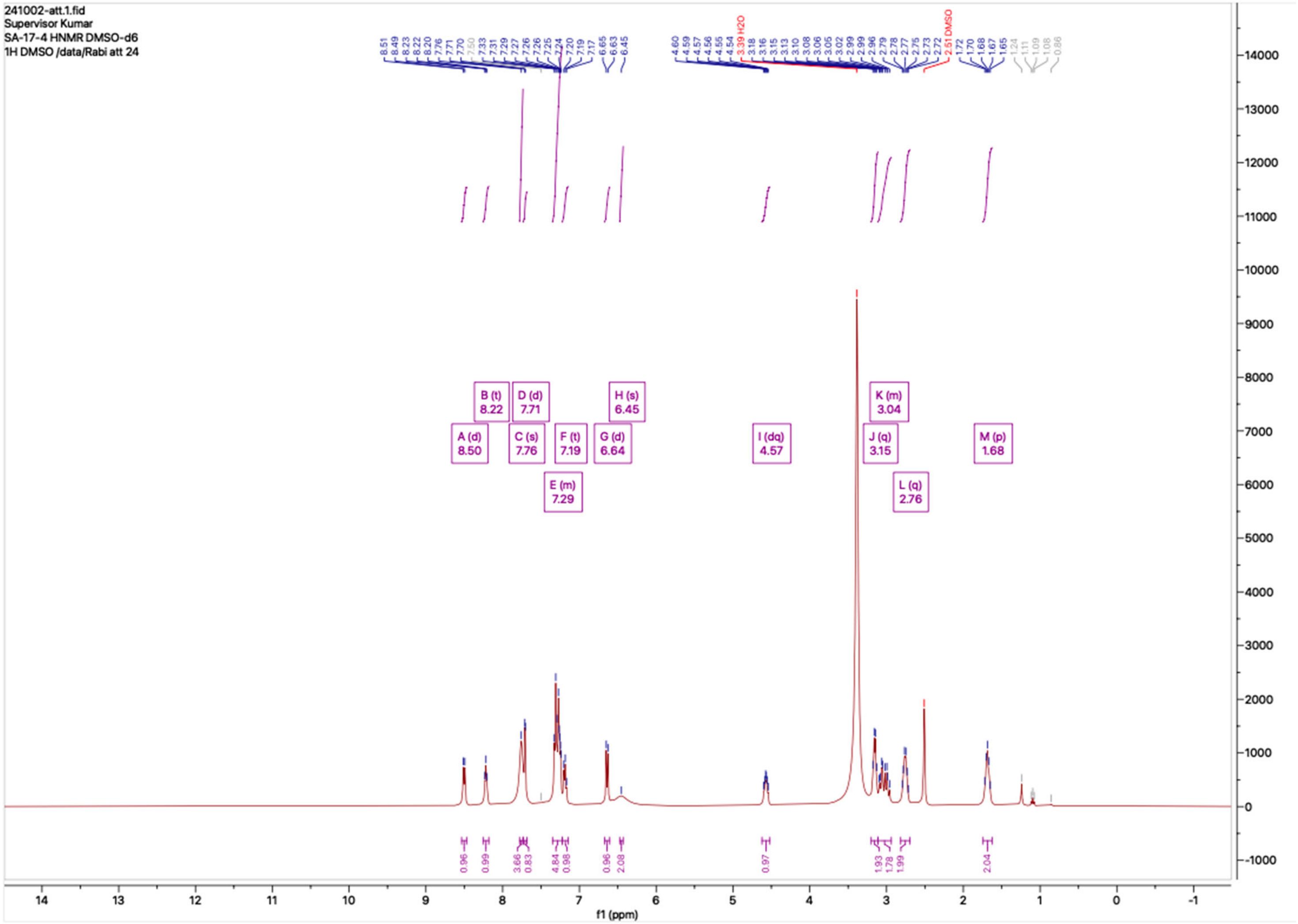

$^{13}$C NMR of (*S*)-2-((1-((3-ammoniopropyl)amino)-1-oxo-3-phenylpropan-2-yl)carbamoyl)-4-bromobenzenaminium (10)

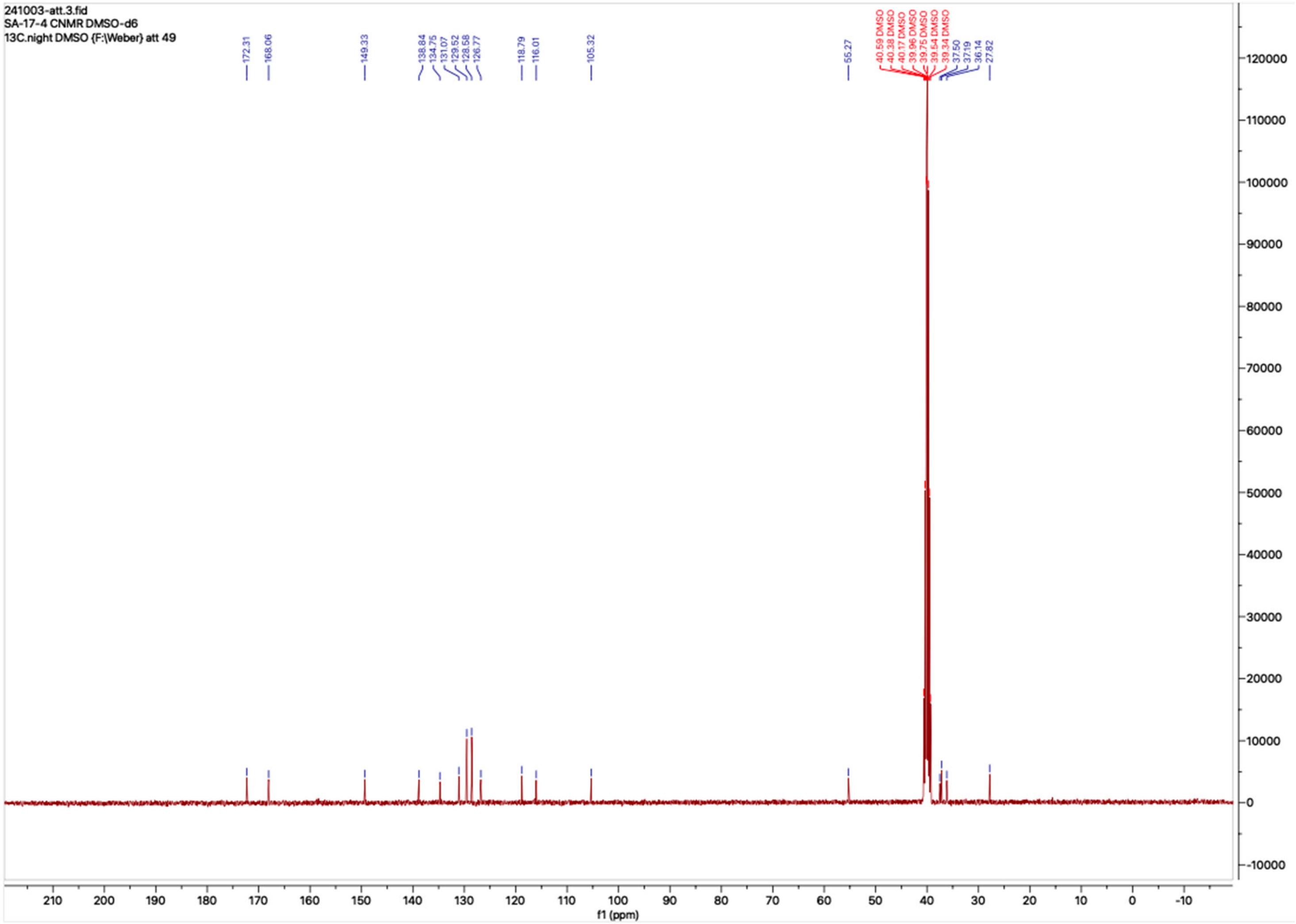

$^{1}$H NMR of di-*tert*-butyl ((*S*)-6-((3-((*S*)-2-(2-([1,1'-biphenyl]-3-carboxamido)benzamido)-3-phenylpropanamido)propyl)amino)-6-oxohexane-1,5-diyl)dicarbamate (23)

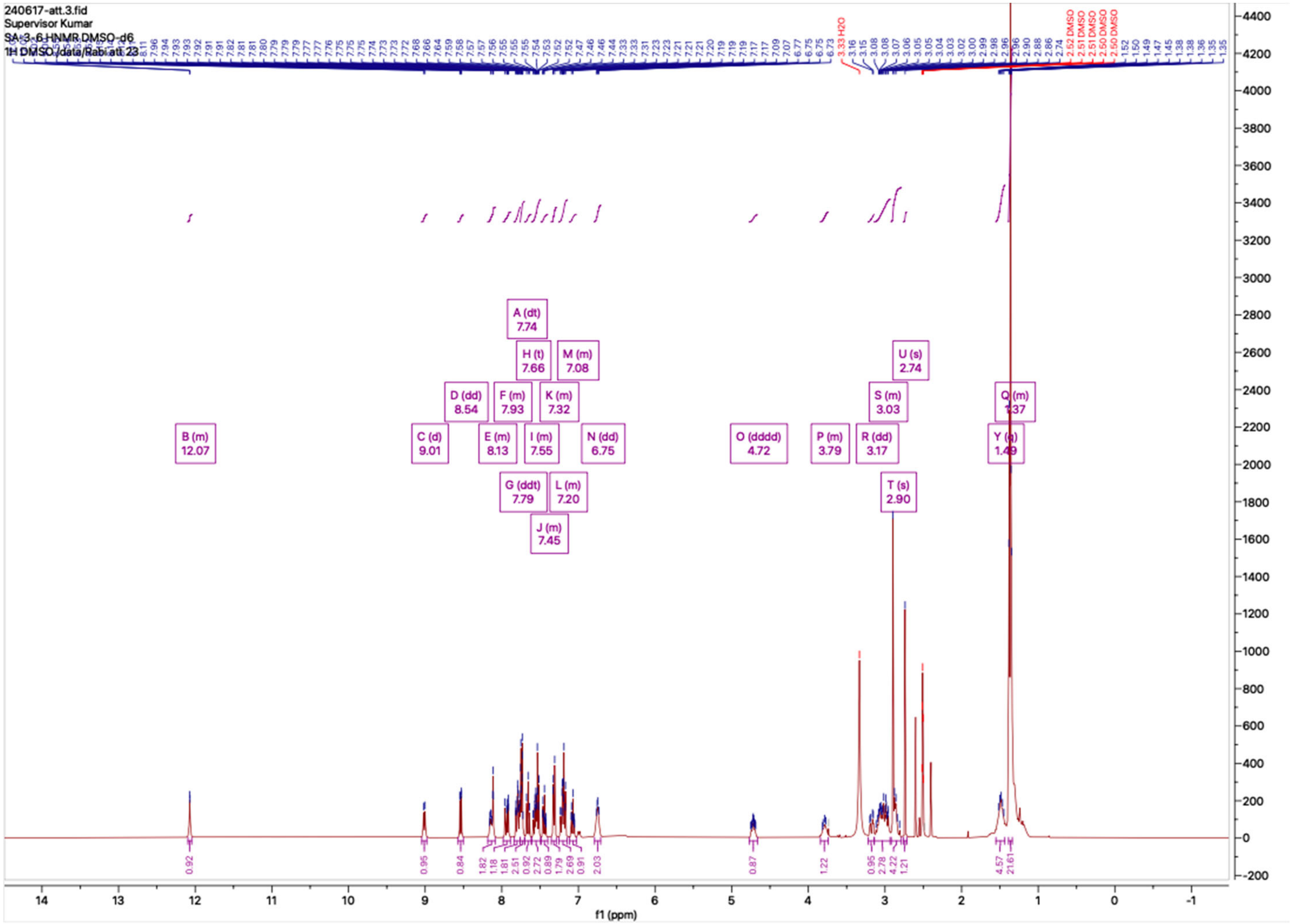

$^{13}$C NMR of di-*tert*-butyl ((*S*)-6-((3-((*S*)-2-(2-([1,1'-biphenyl]-3-carboxamido)benzamido)-3-phenylpropanamido)propyl)amino)-6-oxohexane-1,5-diyl)dicarbamate (23)

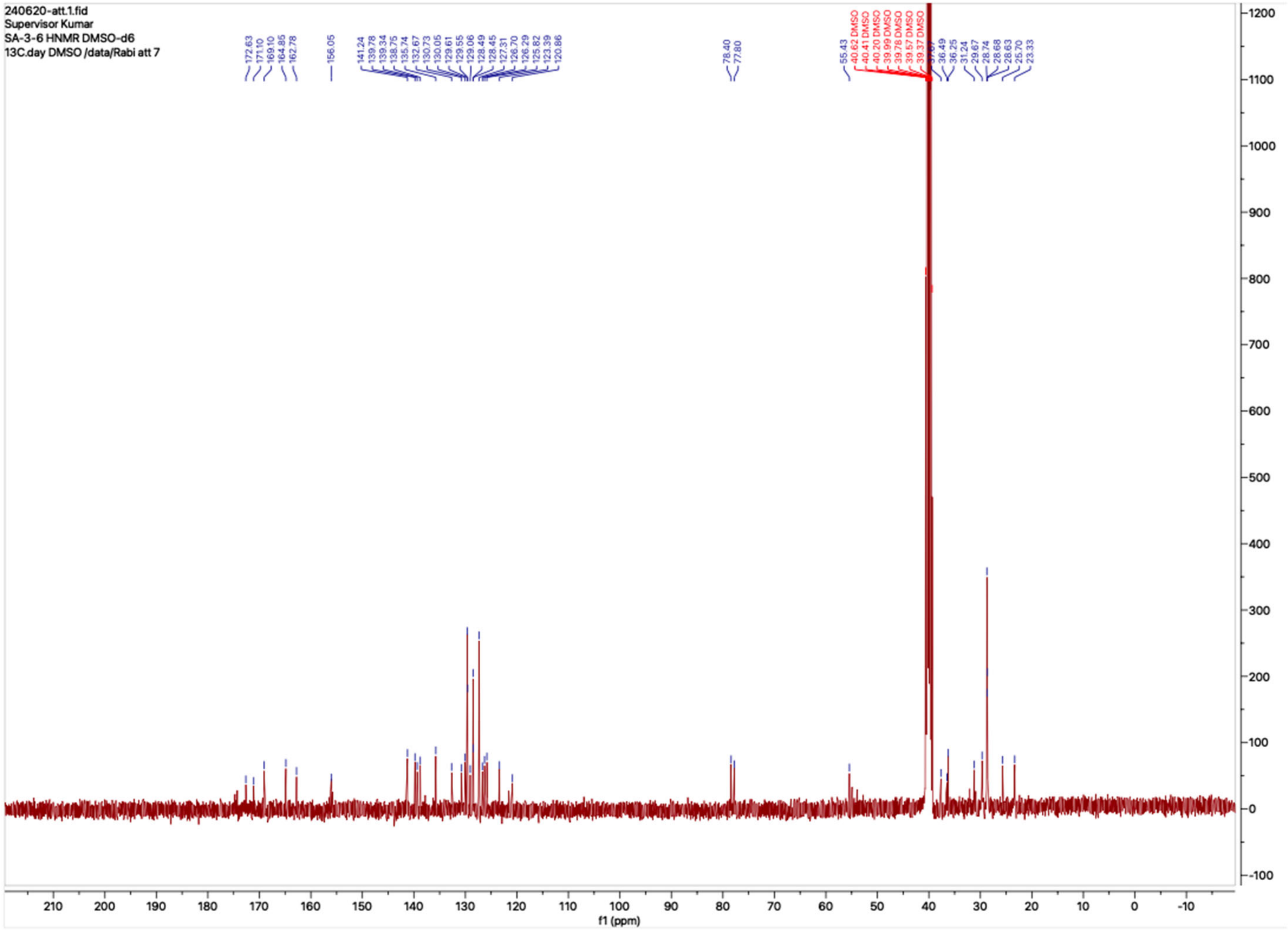

$^{1}$H NMR of (*S*)-6-((3-((*S*)-2-(2-([1,1'-biphenyl]-3-carboxamido)benzamido)-3-phenylpropanamido)propyl)amino)-6-oxohexane-1,5-diaminium (11)

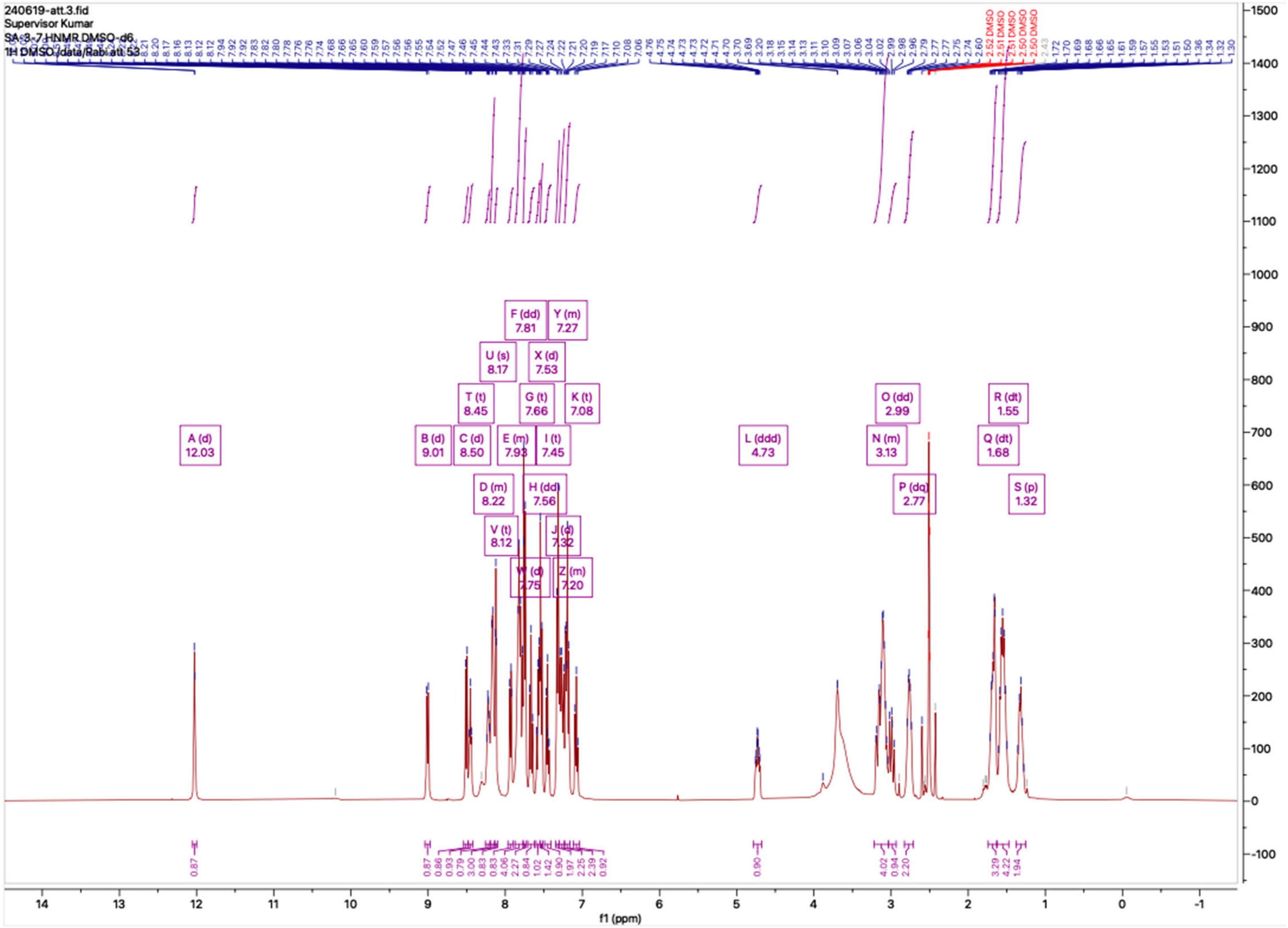

$^{13}C$ NMR of (*S*)-6-((3-((*S*)-2-(2-([1,1'-biphenyl]-3-carboxamido)benzamido)-3-phenylpropanamido)propyl)amino)-6-oxohexane-1,5-diaminium (11)

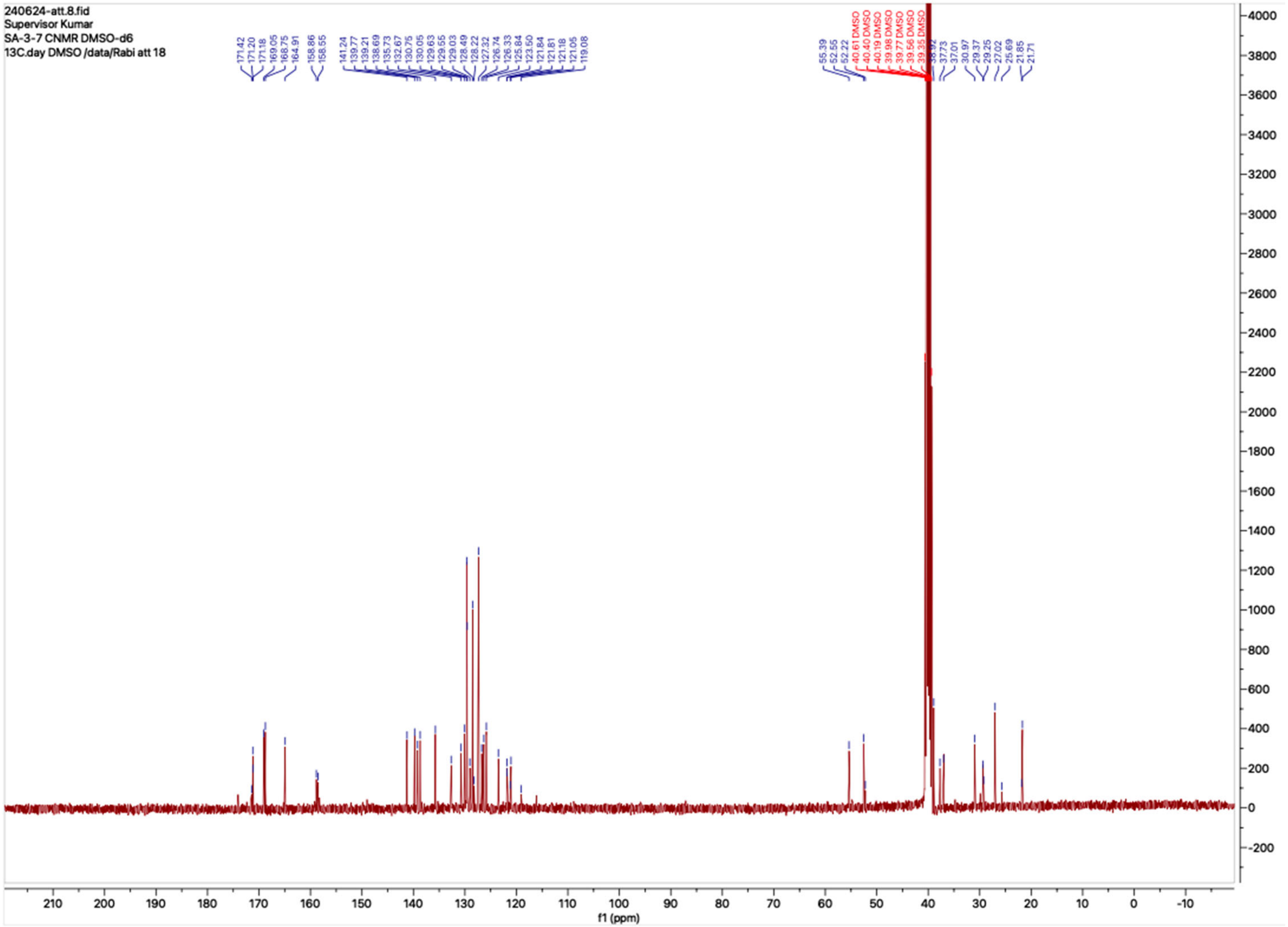

$^{1}$H NMR of (*S*)-*N*-(2-((1-((3-di-*tert*-butyl-guanidinopropyl)amino)-1-oxo-3-phenylpropan-2-yl)carbamoyl)phenyl)-[1,1'-biphenyl]-3-carboxamide (24)

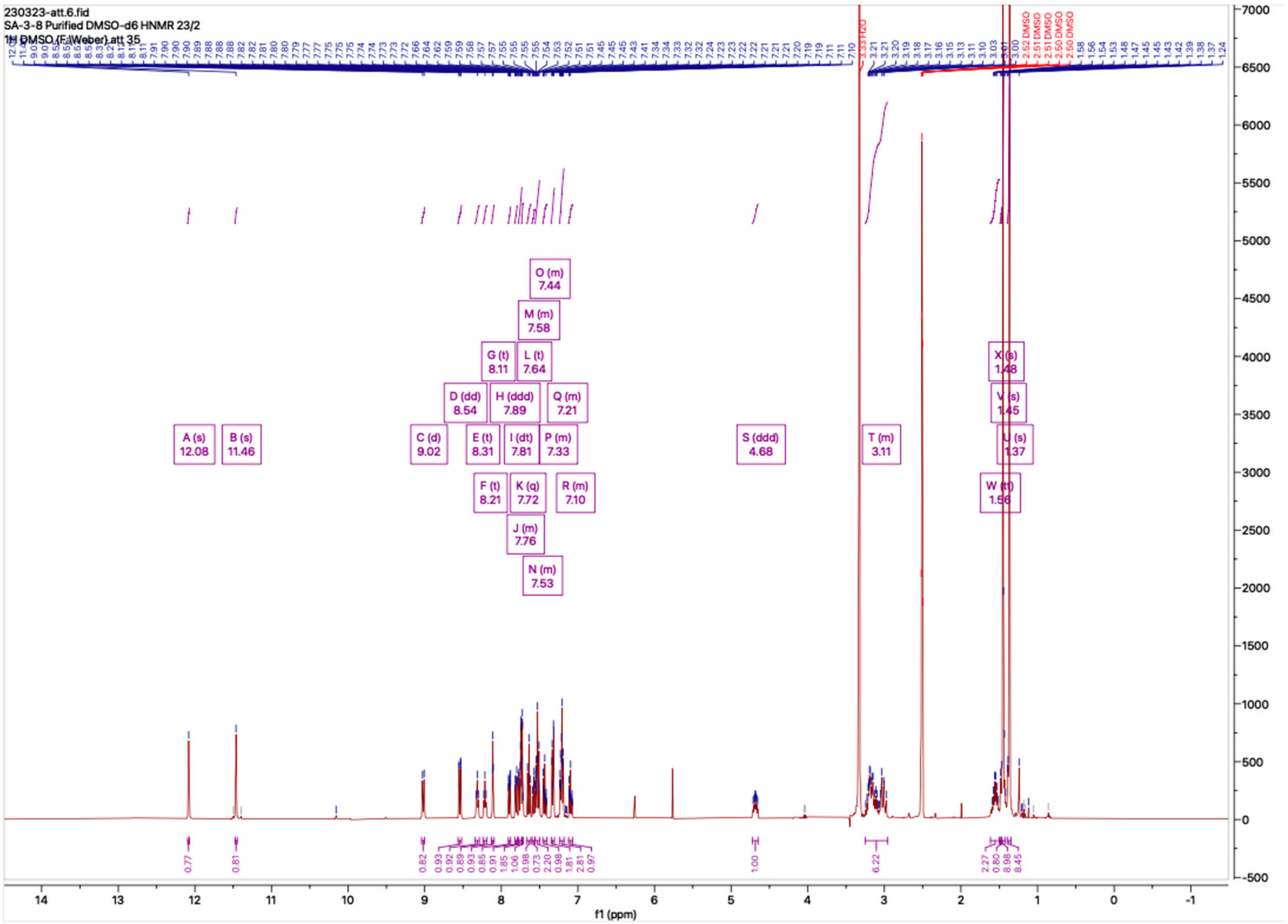

$^{13}$C NMR of (*S*)-*N*-(2-((1-((3-di-*tert*-butyl-guanidinopropyl)amino)-1-oxo-3-phenylpropan-2-yl)carbamoyl)phenyl)-[1,1'-biphenyl]-3-carboxamide (24)

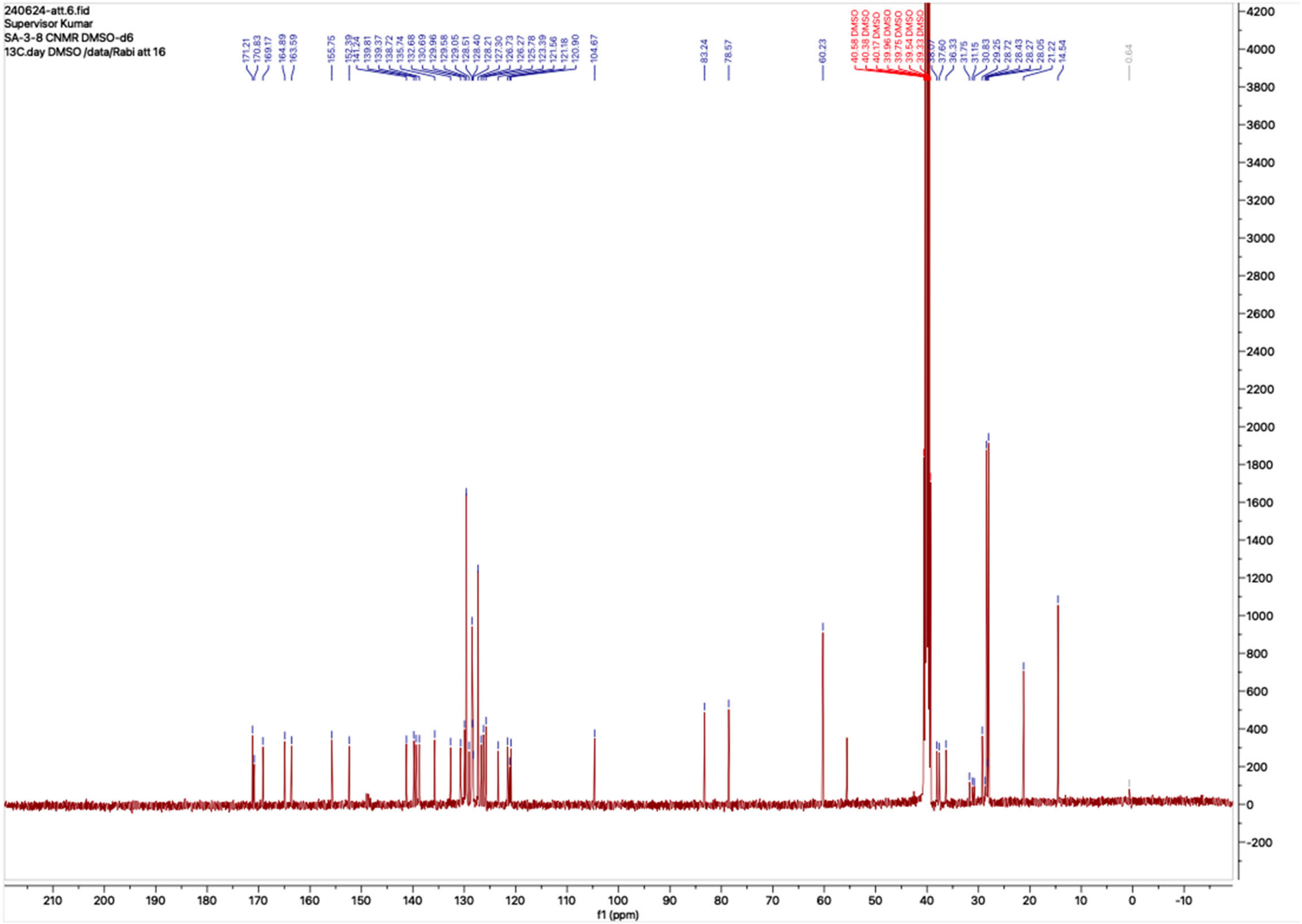

$^1$H NMR of (*S*)-1-(3-(2-(2-([1,1'-biphenyl]-3-carboxamido)benzamido)-3-phenylpropanamido)propyl)guanidinium (12)

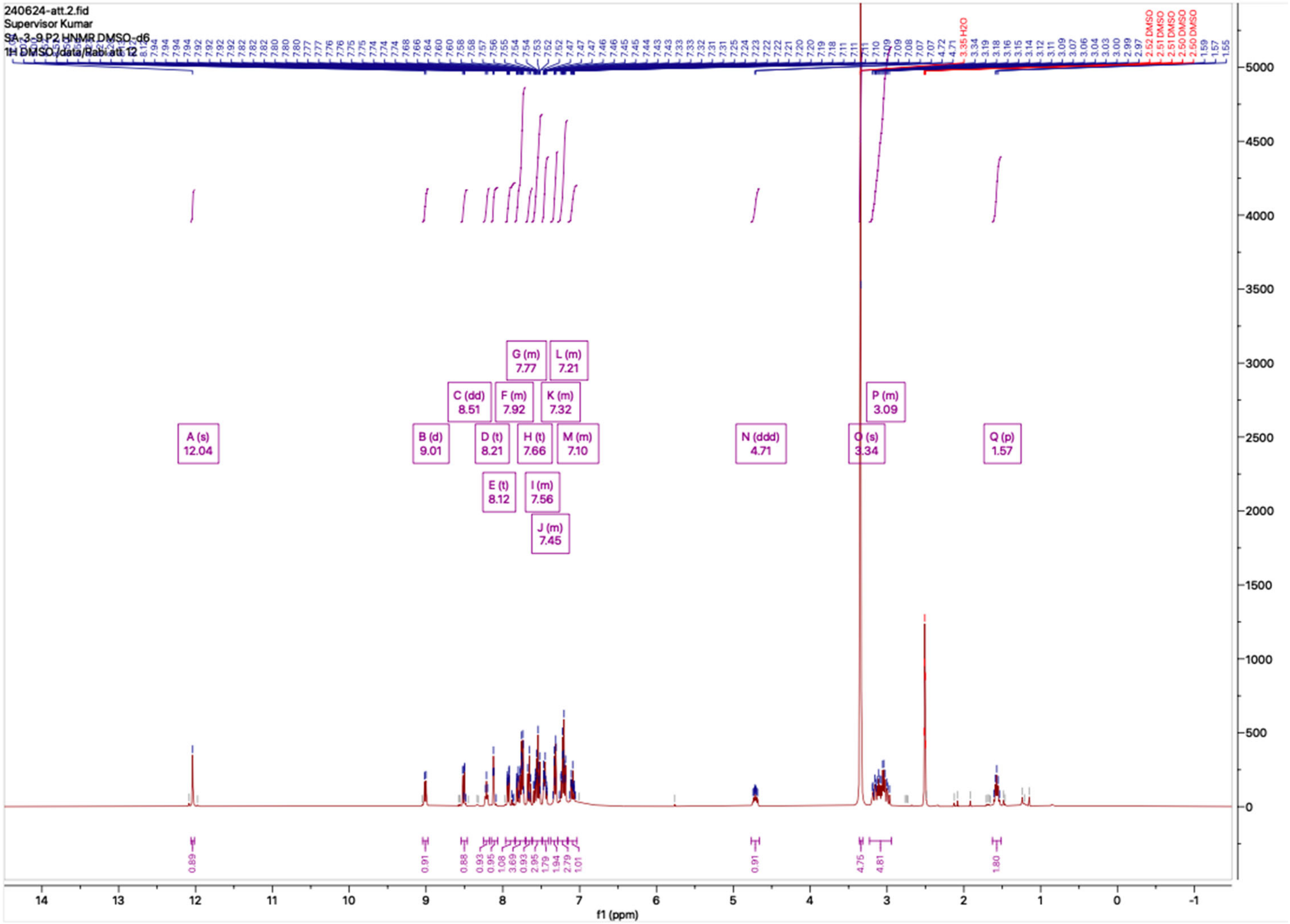

$^{13}C$ NMR of (*S*)-1-(3-(2-(2-([1,1'-biphenyl]-3-carboxamido)benzamido)-3-phenylpropanamido)propyl)guanidinium (12)

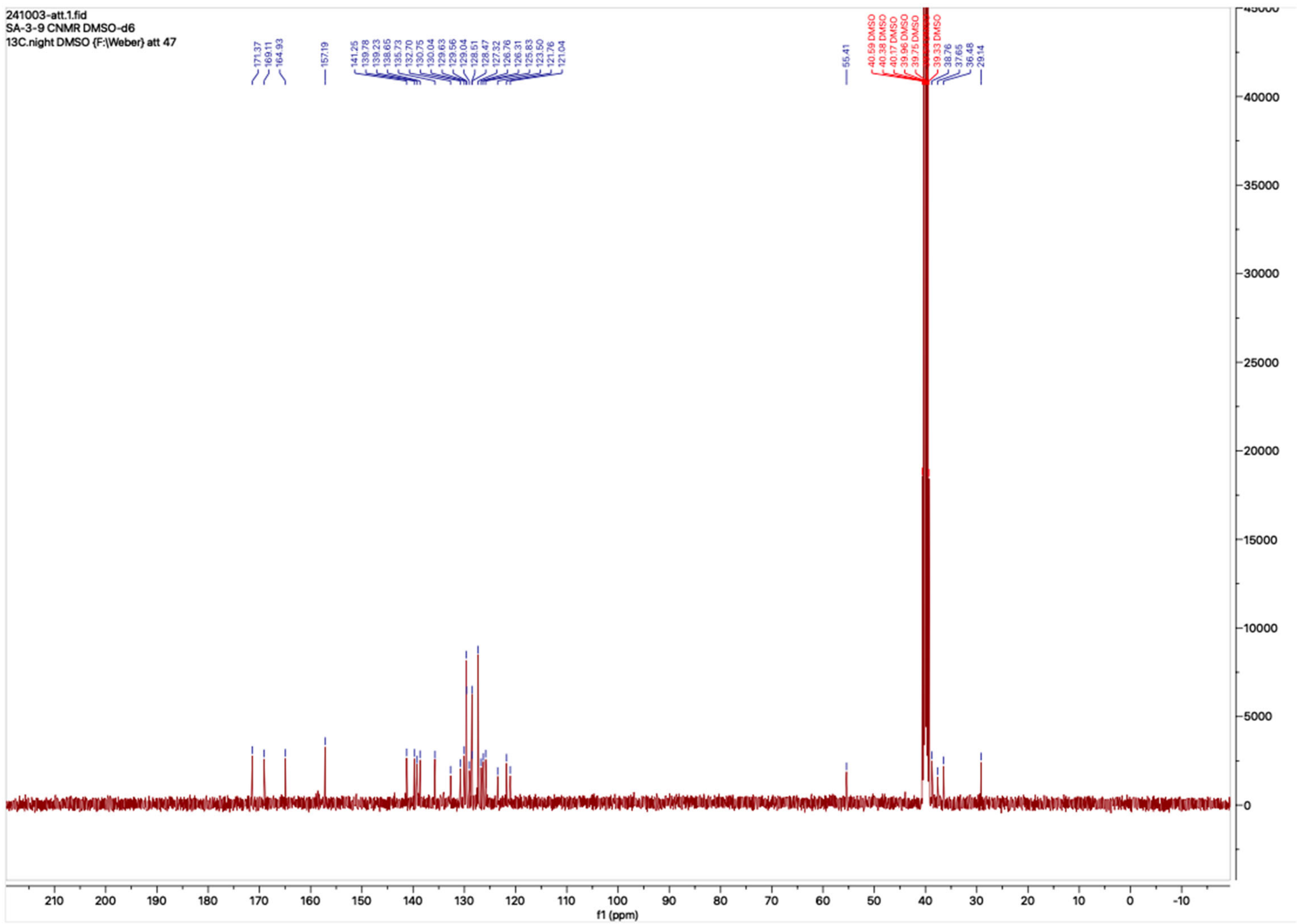

Supplement: Supplementary file 1 [file antibiotics-14-01118-s001.zip › antibiotics-3968841-supplementary.pdf]
